# Identifying hotspots of *S. haematobium* infection following praziquantel treatment during multiple annual mass drug administration campaigns in Zimbabwe

**DOI:** 10.1371/journal.pntd.0013546

**Published:** 2025-09-24

**Authors:** Takafira Mduluza, Grace Zdesenko, Paradzayi Tagwireyi, Caitlin M. Jones, Francisca Mutapi

**Affiliations:** 1 Department of Biochemistry, University of Zimbabwe, Mount Pleasant, Harare, Zimbabwe; 2 Tackling Infections to Benefit Africa (TIBA) Partnership, Ashworth Laboratories, King’s Buildings, University of Edinburgh, Edinburgh, United Kingdom; 3 Institute of Immunology & Infection Research, Ashworth Laboratories, King’s Buildings, University of Edinburgh, Edinburgh, United Kingdom; 4 Department of Geography and Environmental Science, Geo-information and Earth Observation Centre, University of Zimbabwe, Mount Pleasant, Harare, Zimbabwe; 5 Zhejiang University-University of Edinburgh Institute, Zhejiang University School of Medicine, Haining, China; 6 Infection Medicine, Deanery of Biomedical Sciences, Edinburgh Medical School, College of Medicine and Veterinary Medicine, University of Edinburgh, Edinburgh, United Kingdom; IRCCS Sacro Cuore Don Calabria Hospital, ITALY

## Abstract

Urogenital schistosomiasis is contracted from the *Schistosoma haematobium* parasite and is treated with the drug praziquantel (PZQ). Despite MDA interventions, persistent hotspots (PHS) of *S. haematobium* infection have been identified in multiple schistosome endemic African countries but have yet to be characterised in Zimbabwe. This study assessed long-term infection persistence and variability in praziquantel (PZQ) efficacy among school-aged children (6–15 years) in 29 districts of Zimbabwe, using data from MDAs conducted between 2012 and 2017. Metrics included infection prevalence, mean egg count, and treatment efficacy indicators. Two hotspot definitions were applied: (i) prevalence-based persistent hotspots (PPHS), identified by limited reduction or rebound in prevalence; and (ii) efficacy-based persistent hotspots (EPHS), defined by cure rates below 70%. Statistical comparisons between hotspot and non-hotspot (“responder”) districts used regression models, Fisher’s exact test and Mann-Whitney U tests. Analyses revealed four PPHS and six EPHS. PPHS districts exhibited significantly higher baseline prevalence and infection intensity compared with responders (P = 0.043), a pattern not observed for EPHS. Greater distance from freshwater sources was associated with EPHS occurrence (P = 0.016), although this appeared to be an indirect effect of initially high infection intensities. Lower treatment frequency correlated with increased hotspot occurrence, but the relationship was not statistically significant for either hotspot category. Other investigated factors including treatment coverage, timing of drug administration and ecological suitability for intermediate host snails showed no significant association with hotspot status. The elevated initial prevalence and infection intensity in PPHS suggest these indicators could be used for early hotspot identification, enabling targeted adjustments in intervention strategies. The findings underscore the limitations of relying solely on preventive chemotherapy in high-transmission settings. Integrating complementary measures such as water, sanitation and hygiene (WASH) interventions and snail control may improve outcomes, particularly in hotspot areas. In conclusion, the persistence of *S. haematobium* hotspots in Zimbabwe highlights the need for adaptive, integrated control approaches aligned with the WHO’s 2030 roadmap. Monitoring baseline epidemiological indicators could facilitate earlier detection of persistent transmission foci, guiding more effective and sustainable schistosomiasis control.

## Authors summary

Urogenital schistosomiasis is a debilitating parasitic disease, commonly found in sub-Saharan Africa. Zimbabwe regularly administers praziquantel (PZQ), the primary treatment for this disease. Despite a heavy reliance on PZQ, persistent hotspots (PHS) of *S. haematobium* infection have been identified in other countries in Africa. Yet, no study has assessed the emergence of PHS in Zimbabwe or the factors causing them. We investigated PHS using measures of infection prevalence and PZQ efficacy using data collected from school-aged children in Zimbabwe during MDAs administered between 2012 and 2017. We identified four PHS of infection prevalence, all of which were found to have significantly higher baseline prevalence and infection intensity than non-hotspot areas. We also detected six PHS of decreasing PZQ efficacy in which increased distance to a freshwater source was significantly associated with hotspot occurrence. Additionally, although the decreased frequency of PZQ treatment was associated with more hotspot occurrences, it was not significant. Additional risk factors of persistent infections including coverage/season of treatment and favourable host snail habitats were evaluated for their contribution to these hotspots using multiple statistical tests, but no further significant associations were detected. These findings contribute toward improving schistosome control in Zimbabwe and further informing on PHS of this disease. We highlight the importance of integrated control interventions, such as water, sanitation and hygiene (WASH) and snail control, to complement preventive chemotherapy, particularly in hotspot settings. Overall, our findings call for an integrated control approach to meet the targets set by the new WHO roadmap.

## Introduction

Schistosomiasis is Africa’s second most prevalent parasitic disease, infecting over 236 million people annually [1]. One of the most common presentations is urogenital schistosomiasis [2], contracted from the species *Schistosoma haematobium* via an intermediate host of freshwater *Bulinus globosus* snails [3]. Human transmission occurs through contact with contaminated freshwater sources via routine day-to-day activities like washing, working, and playing [4]. Globally, schistosome infections are treated with the drug praziquantel (PZQ), with widespread usage across Africa to prevent persistent infections and reduce the associated morbidity of this disease [5]. Chronic clinical manifestations of *S. haematobium* include dysuria, hematuria, injury of the genital tract increasing susceptibility to human immunodeficiency virus (HIV), and even bladder cancer [6].

Schistosomiasis control programs rely heavily on the mass drug administration (MDA) of PZQ to treat exposed individuals and retain control throughout successive treatments [7]. Like several other African countries, Zimbabwe has been administering PZQ as part of a national helminth control programme for nearly a decade. A comprehensive study of Zimbabwe’s 2012–2017 control program concluded there was a significant reduction in the national prevalence of *S. haematobium* (31.7% to 0%) after six rounds of MDAs, highlighting the effectiveness of a successful MDA strategy [8]. Unfortunately, due to the COVID-19 pandemic, there has been a halt on multiple MDA programs across Africa, as is the case for Zimbabwe. Currently, there is uncertainty about the future implications of these disruptions in eliminating schistosomiasis [9,10]. However, as MDA programs are generally well accepted among children, parents, teachers, health workers and members of the community [11] their implementation remains a technically feasible and cost-effective intervention [12], and targeted re-introduction of these programs would be favorable to regain control of schistosome infections in Zimbabwe. Consequently, there is an urgent need for data and tools to identify and predict potential schistosomiasis hotspots caused by exacerbated transmission factors or differential PZQ efficacy to aid future control programs.

Despite MDA interventions, there have been hotspots of persistent *S. haematobium* infection identified in multiple schistosome endemic countries, with the differences between hotspot areas (persistent infections) and responder areas (clears infections) evaluated. For instance, Pennance *et al.* detected hotspots of increased *S. haematobium* prevalence in regions of Zanzibar during MDAs and a study by Sang *et al.,* in western Kenya detailed the detection of infection hotspots during the implementation of a control program [13,14]. Both studies discussed and evaluated the various transmission factors contributing toward a hotspot of infection, and the importance of disease mapping in identifying hotspots. Presently, no study has assessed the possibility of hotspots in Zimbabwe during the control efforts. The usual way to monitor and evaluate success of MDA is through pooled prevalence of infection at district or province level. However, this can miss the heterogeneity in MDA success which can occur at a village or community level. Due to lack of information, there is a need to monitor infection levels and the efficacy of PZQ in treating schistosomiasis; a point also highlighted by multiple hotspot evaluations. For example in Senegal, low drug sensitivity of parasites in the population was observed over numerous treatments during a study by Danso-Appiah *et al.,* [15]. Furthermore, the authors emphasised the necessity of evaluating persistent infection prevalence and the population’s reaction to PZQ, regardless of whether this is due to natural variation in parasite drug sensitivity or the development of drug resistance.

Given the numerous reported definitions of a ‘hotspot’ in infectious disease epidemiology, identifying persistent hotspots (PHS) of schistosome infection will play an important role in the research and policy of planning, allocation, and implementation of resources to eliminate schistosomiasis [16]. In this study, we aimed to identify hotspots of *S. haematobium* infection in Zimbabwe using six years of annual MDA data. We evaluated these hotspots using two definitions, with the terms separated by the cause of the hotspot: persistent hotspots of *S. haematobium* prevalence (PPHS) and persistent hotspots of decreased PZQ efficacy (EPHS). To determine PPHS on a district/village level, we used infection intensity and prevalence measures to evaluate if there were any patterns in transmission or contributing risks that lead to these hotspots. To determine EPHS we assessed the efficacy of PZQ using the cure rate (CR) and egg reduction rate (ERR). We then determined what factors affect the success of a PZQ treatment in the Zimbabwean study population.

## Methods

### Ethical approval and consent

Ethical approval was obtained from the Medical Research Council of Zimbabwe (MRCZ/A/1710) and permission to conduct the study in each district was obtained from the Ministry of Health & Child Care in Zimbabwe. Participants were recruited into the study voluntarily and were free to withdraw at any time with no further obligation. Written consent was provided by parents/guardians of the participants upon discussion of the purpose of the study in the local language (Shona).

### Study design and inclusion criteria

**Fig 1:**
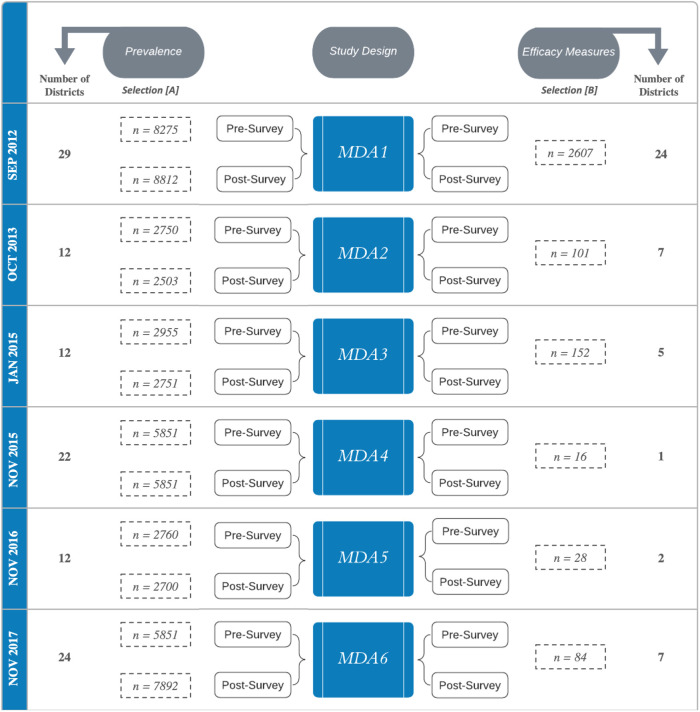
Study design depicting the two-pronged approach of this analysis, containing the number of included children (*n*) from each mass drug administration (MDA) survey. Inclusion criteria for each prong of this study is as follows: [A] to determine the prevalence, theselection was determined by those children who provided urine samples for parasitological quantification at either the pre- or post-MDA survey, and [B] to assess efficacy measures those included were treated children (as confirmed by the school MDA registers) who were positive for schistosome infection and were followed at both pre- and post- MDA. The number of districts included in each survey is labelled, along with the date of each MDA.

The inception and execution of the schistosomiasis control program in Zimbabwe are described by Mduluza *et al.,* [8]. Briefly, the prospective study design followed a cohort of primary school-aged children (SAC), aged 6–15 years old, with the MDAs performed annually from 2012 to 2017 between September and November (Fig 1). One exception was the third MDA, which was delayed due to logistical problems and was completed in January 2015. Throughout this study, the nomenclature of each mass drug administration was “MDA”, followed by the number representing the treatment time, e.g., MDA1. SAC from 35 schools across 29 districts of Zimbabwe were recruited for this study, following the WHO guidelines detailing the pre-MDA sampling of schools. The sentinel sites were purposely selected to assess the *S. haematobium* egg clearance patterns of the MDA program in Zimbabwe across three endemicities of schistosomiasis infection prevalence as described by WHO [7]; low (*x* ≤ 10%), moderate (50% ≥ *x* ≥ 10%) and high (*x* ≥ 50%). For each MDA, the SAC could be included in one or both arms of this study: [A] assessment of persistent hotspots of *S. haematobium* prevalence (PPHS) or [B] persistent hotspots of decreased *S. haematobium* egg clearance patterns (EPHS). The inclusion criteria for these assessments are as follows: [A] to determine the prevalence, the selection of samples was determined by those who provided three urine samples for parasitological quantification at either the pre- or post-MDA survey, or [B] treated children (as confirmed by the school MDA registers), who provided three urine samples, were positive for schistosome infection at baseline and were followed at both pre- and post- MDA. Missing data for either of these arms of the study was predominantly due to children completing primary school and progressing to secondary school, in addition to those who transferred to other distant schools. The overall SAC sample sizes and the number of districts included in each aspect of the study are displayed in Fig 1.

### Antihelminthic treatment and coverage of the mass drug administrations (MDAs)

The standard WHO treatment with PZQ for schistosomiasis in Zimbabwe is a 40mg/kg dose for every child [17]. The nurses and school health coordinators administered the treatment and health checks in the national helminth control team, as is the standard Ministry of Health practice in Zimbabwe during the annual program. Each treatment is tailored to the child using a PZQ dose pole, regardless of infection status. To ensure successful administration, the children ate bread and juice to enhance absorption of the drug and each child was checked to confirm they had swallowed the tablet. To ensure accurate recording of each MDA and to distinguish non-compliance from treatment failure, the number and brand of tablets administered were recorded in the MDA register. All children followed up in this study were confirmed to have received PZQ treatment when administered at each MDA using these records. The coverage of each MDA was available at district level for the country’s annual control program from the ESPEN database (http://espen.afro.who.int/) for the duration of the study.

### Sample collection and parasitology

The *S*. *haematobium* parasitology data was generated using the collection procedure previously described in detail by Mduluza *et al.* [8], and the samples were processed using the standard method of urine filtration and microscopy methods of Mott *et al.,* [18]. Urine was collected from participants on 3 consecutive days between 1000hrs and 1400 hrs, which is the time range where a high number of parasite eggs is excreted [19]. A whole faecal sample was also collected on one occasion. *S. haematobium* infection was detected using the urine filtration technique in which 10 ml urine sample was filtered through a nitrile filter with a pore size of 12 µm. The intensity of *S. haematobium* infection was categorised as light (<50 eggs/10mL) or heavy (≥50 eggs/10mL).

### Approach and identification of hotspots of *S*. *haematobium*

To identify hotspots of *S*. *haematobium* infection, we separated the hotspot assessments into PPHS and EPHS. To describe PPHS of *S*. *haematobium* infection two approaches were selected. Approach A, was defined as a change in WHO prevalence risk category (high, ≥ 50%; moderate, 10–49%; and low < 10% prevalence) which describes a PPHS as a meaningful decline in a prevalence risk category, an approach which has been used to assess hotspots in studies in Kenya and Tanzania [19]. For instance, a decrease from 55% in pre-MDA1–42% in pre-MDA4/5/6 would be a meaningful decline in WHO risk category, but from 42% to 11% would be designated as a PPHS. Villages/ districts that did not meet the definition of a PPHS were classified as having meaningful declines in prevalence and were discussed in this context.

Approach B was defined as a change in a site with a prevalence ≥ 10% pre-MDA1 that remained ≥ 5% post-MDA4/5/6. This method was described by a study in Zanzibar which determined hotspots of *S. haematobium* [20]. Villages/ districts that did not meet the definition for a PPHS were classified as having meaningful declines in prevalence and were discussed in this context. Only data from sites treated more than twice within four years were assessed, as analysed by Kittur *et al.,* [21,22]. This was combined with additional modelling studies by Kittur *et al.,* [20], which indicated that prediction of PPHS before even the third year of an MDA is possible as some sites had been treated at least twice by this timepoint. Therefore, we included all data from pre-MDA4 to post-MDA6, allowing the analysis of MDA4, MDA5 and MDA6.

To describe an EPHS, we used Approach C. It classified a ‘lower than expected’ *S. haematobium* egg clearance (EC) of <70% after treatment with PZQ as an EPHS. This was determined to be the threshold at which reduction in *S. haematobium* egg clearance required investigation based on a study in Senegal [23] and allowed discussion on potential contributing factors to decreased *S. haematobium* egg clearance patterns.

### Identification of *S*. *haematobium* transmission sites

Data from a study assessing the spatial distribution of the freshwater snail *Bulinus globosus*, the intermediate host involved in the transmission of *S. haematobium* by Pedersen *et al.* [24,25], was extrapolated. The data was overlaid with the sample sites used in this study and transmission scores for both 1988 and 2012 were then assigned to these sites (S1 Table). The resultant datasets extrapolated provided the suitability for snail habitats in Zimbabwe in 1988 and 2012 for risks and for increased transmission. The transmission scores based on habitat suitability (from 1988 and 2012) were selected as a predictor for human infection. The greater the suitability of the snail habitat the more likely for potential transmission from a *Bulinus globosus* vector. The contribution of the distance to the nearest freshwater source as a transmission site was also evaluated. The distance from the sample site to the nearest large waterway was identified as the snail vectors are found in freshwater, and transmission occurs when there is contact with contaminated water. The detection of the closest waterway was obtained using a shapefile containing location data on all the larger bodies of water in Zimbabwe, including the rivers, streams, dams, and lakes, along with the coordinate data of each sample site. These distances were calculated using the ‘Distance to the nearest hub’ feature in QGIS Version [3.22.2], with the results exported to Microsoft Excel and the shortest distance for each site to the watercourse identified for further analysis.

### Statistical analysis

The raw data obtained by Mduluza *et al.,* [8] was recorded on hard-copy field sheets and entered into a Microsoft Excel spreadsheet. The spreadsheet was separately reproduced for this study and double proof-read to include all children treated from pre-MDA1, post-MDA1 to post-MDA6. The infection prevalence and mean egg counts of *S. haematobium*, including the 95% confidence intervals (CI), were calculated for each survey for both village and district. The arithmetic mean egg counts of infection, including counts from both positive and negative children, were used in the analysis. To determine the treatment efficacy in clearing *S. haematobium* infections, the egg reduction rate (ERR), a measure of the change in parasite egg burden upon treatment, a measure of those cured of infection upon treatment, were calculated for each time point for each village and district [26,27]. The ERR and CR calculations used only data from treated children who were positive for schistosome infection and were followed at both pre- and post-MDA, as described above. Choropleth maps of the infection prevalence, CR’s and ERR’s were generated using a shapefile of Zimbabwe, with the GPS coordinates of the sentinel sites obtained during the collection of the primary data using QGIS, Version [3.22.2].

Significant changes between the prevalence and mean egg counts were analysed using Minitab Statistical Software 20 and SPSS statistical software (IBM, version 23), respectively. Due to the non-parametric nature of the data, differences in *S. haematobium* prevalence between pre- and post-MDA time points were analysed using a paired McNemar’s test, and differences between annual MDAs were tested for significance using a Fisher’s exact test [28]. The differences in *S. haematobium* mean egg counts between pre- and post-MDA time-points were analysed using a paired two-way Student’s t-test, and the difference between annual MDAs were tested for significance using an unpaired Student’s t-test. Additionally, a χ^2^ test was used to investigate significant association between individuals with a CR < 100% and their baseline infection intensity, post-MDA intensity, post-MDA status (cleared or not cleared infection). The association between frequency of treatment or the month of MDA and a hotspot was assessed using Fisher’s Exact tests, whereas the association between hotspot and the baseline prevalence was tested using the Mann-Whitney U-test. For all analyses in this study, a significant threshold of P < 0.05 was established.

Linear and logistic regressions were performed to identify potential spatial drivers of *S. haematobium* infection using the combined MDA survey dataset. Five separate stepwise linear regression models were used to evaluate the dependent variables (prevalence, mean egg counts, ERRs, CR), as described in S2A Table, with different combinations of predictors assessed. A binary logistic regression model (S2B Table) was also used, where 1 represented positive schistosome egg count, and 0 representing no schistosome infection detected, to test the influence of two independent variables (distance and transmission scores) on the detection of schistosome infection. An ordinal logistic regression model (S2C Table) was also performed to test the influence of distance and transmission scores on the WHO risk category (Table 2). The ordinal dependent variable was coded as 0 = no schistosome infection, 1 = low prevalence (<10%), 2 = moderate prevalence (10–49%), 3 = high prevalence (≥50%).

**Table 2:** Results of different approaches to determine persistent hotspots of *S. haematobium* prevalence (PPHS).

	Approach A	Approach B			
*MDA*	*1–6*	*1–4*	*1–5*	*1–6*	
District (Village)	Chipinge (Chitepo)	Mount Darwin (Bemberi)	Chiredzi (Mareya)	Chipinge (Chitepo)	Buhera (Masocha)
**Number of MDAs received by this point**	2	3	5	**2**	2

## Results

### Prevalence and mean egg counts of *S. haematobium* infection

Twenty-nine districts were surveyed for *S. haematobium* infection, with the prevalence, arithmetic mean egg count, and proportion of heavy to light infections calculated first for each village and then for each district (S3 Table). To easily observe the progression of *S. haematobium* infection during the MDAs, S1A & S1B Fig shows the prevalence of each district and the location and infection intensity of each village as a choropleth map.

***Changes in prevalence and egg counts pre and post MDA1****:* Of the twenty-nine districts investigated in this study, only twenty-five (86.2%) were positive for *S. haematobium* at the initial survey in 2012. The baseline prevalence across the country was shown to be 33.5% (CI: 25-42.2%), with the prevalence of each district ranging from 0% to 73.6%. Pre-MDA1, the mean egg count across the twenty-nine districts was 24.9 eggs/10mL (CI: 16.9-32.9), with values ranging between 0 to 88.9 egg/10mL. Only the Muzarabani district (prevalence of 73.6%) had a mean egg count of heavy intensity, having the largest initial parasite burden at any of the surveyed populations at 88.9 eggs/10mL (CI: 75.33-102.53). Of the twenty-five districts positive at pre-MDA1, twenty-four districts significantly reduced (P < 0.05) in mean egg count, and twenty-three districts significantly reduced (P < 0.0001) in prevalence after PZQ treatment (**Table 1**). There were two exceptions of note. Mount Darwin had an initial prevalence of 66% and failed to significantly decrease prevalence (P = 0.266) from pre-MDA1, only reducing to 24.3% post-MDA1. However, 44% of the Mount Darwin population in the pre-MDA1 survey carried heavy infections. This was fewer heavy infections than in the district of Muzarabani, whose percentage of the population carrying heavy infections was the highest of all surveyed in pre-MDA1 with 66%. Yet, all those treated in MDA1 in Muzarabani had fully cleared infection after treatment. Regardless, Mount Darwin significantly reduced the mean egg count, from 49.7 eggs/10mL (CI: 39.1-60.3) to 4.96 eggs/10mL (CI: 3.2-6.7) at post-MDA1. The second exception to prevalence reduction was the district of Nkayi, which was categorised as having low endemicity at 6.2%. Still, it underwent a significant increase (P < 0.0001) in prevalence in the post-MDA1 survey to 7%. Furthermore, the mean egg count for Nkayi was also not found to change significantly (P = 0.265) in comparison to the other twenty-four districts post-MDA1, with a slight reduction from 1.14 eggs/10mL (CI: 0.19-2.1) to 0.77 eggs/10mL (CI: 0.33-1.2). Overall, post-MDA1, five (17.2%) districts in Zimbabwe still carried infection, including Mount Darwin, Nkayi, Rushinga, Shamva, and Mwenezi.

**Table 1:** Measures of praziquantel efficacy (PZQ) against *Schistosoma haematobium* after the treatment during a mass drug administration (MDA).

*District (Village)*	*Mt Darwin (Bemberi)*		*Rushinga (Mazowe Bridge)*		*Nkayi (Gonye)*	*Chiredzi (Mareya)*
**MDA**	**1**	**3**	**1**	**2**	**1**	**5**
**Sample Size**	126	9	110	23	5	27
**Cure Rate (%)**	68.25	22.22	99.09	43.48	0	96.29
**Egg Reduction Rate (%)**	91.72	28.57	99.99	58.91	61	99.06
**Pre-Mean Egg Count (95% CI)**	80.13 (65-95.27)	25.93 (7.16-44.69)	43.89 (30.78-57)	3.74 (2.22-5.26)	37.27 (-10.08-84.61)	1.31 (1.03-1.59)
**Post-Mean Egg Count (95% CI)**	6.64 (4-9.27) ^[A]ii^	18.52 (1.61-35.43) ^[A]i^	0.005 (0-0.01)	1.54 (0.3-2.77)	14.53 (4.16-24.91)	0.01 (-0.01-0.04)
**Light: Heavy Infections (%)**	56:44	78:22	81:19	100:0	60:40	100:0
***District (Village)***	***UMP (Kafura)***		***Shamva (Total)***		***Shamva (Gono)***	***Shamva (Chihuri)***
**MDA**	**2**	**5**	**1**	**Village** **analysis**	**1**	**1**
**Sample Size**	9	1	194		149	45
**Cure Rate (%)**	66.67	0	97.42		96.64	100
**Egg Reduction Rate (%)**	82.41	4.17	99.63		99.57	100
**Pre-Mean Egg Count (95% CI)**	4 (1.45-6.55)	8 (8–8) ^[B]^	76.76 (58.21-95.31)		85.58 (62.7-108.45)	47.56 (22.49-72.63)
**Post-Mean Egg Count (95% CI)**	0.7 (0.11-1.3)	7.67 (7.67-7.67) ^[B]^	0.28 (-0.11-0.68)		0.37 (-0.15-0.88)	0 (0-0)
**Light: Heavy Infections (%)**	100:0	100:0	66:34		64:36	76:24

***Changes in prevalence and egg counts pre and post MDA2****:* Twelve districts were surveyed at baseline, with seven (58.3%) districts positive for *S. haematobium.* Only five districts were found to significantly decrease in prevalence (P < 0.0001), and four districts significantly reduced in mean egg count (P < 0.05) by post-MDA2. Two districts failed to reach significant prevalence reduction. Muzarabani, which had the highest prevalence pre-MDA1, significantly increased (P < 0.0001) in prevalence by 6.1% in post-MDA2 compared to pre-MDA2 and post-MDA1. All were light infections, with a significant increase in mean egg count (P < 0.01) of 0.06 eggs/10mL (CI: 0.02-0.1). Secondly, in the UMP district there was a significant increase (P < 0.0001) in prevalence from 6.8% to 11.2% following the second round of PZQ treatment, which increased from a low to moderate risk category. Additionally, there was no significant decrease in the mean egg count (0.17 to 0.15 eggs/10mL, P = 0.725). Though, there was a significant decrease in mean egg count at pre-MDA2 than pre-MDA1, with 0.17 eggs/10mL (CI: 0.05-0.29) compared to 48.37 eggs/10mL (CI: 36.07-60.66) at the initiation of this study. One district, Rushinga, did not appear to have any significant change in mean egg count (P = 0.067) post-MDA2, with a slight but insignificant increase during MDA2 from 0.75 eggs/10mL (CI: 0.49-1) to 0.98 eggs/10mL (CI: 0.62-1.35). Additionally, although there was a significant decrease in prevalence (P < 0.0001), the decrease from 21.6% to 17.6% resulted in Rushinga remaining moderately endemic at the end of MDA2, with all infections classified as light. The district of Nkayi increased in both prevalence (by 1.2%) and mean egg count (by 1.19 eggs/10mL) from pre-MDA1 to pre-MDA2, with heavy infections increasing by 9%, despite one round of MDA. Both increases were insignificant (P = 0.719, P = 0.14), and this district had cleared all infections post-MDA2. After two rounds of MDAs, only three (17.2%) districts in Zimbabwe still carried infection, including Muzarabani, Rushinga, and UMP.

***Changes in prevalence and egg counts pre and post MDA3****:* Twelve districts were surveyed pre-MDA3, and seven (58.3%) were positive for *S. haematobium.* Three districts had a resurgence in infections compared to pre-MDA2. Mount Darwin and Muzarabani were negative for infection pre-MDA2 but significantly increased in prevalence (P < 0.0001) at pre-MDA3 to 11.2% and 10.4%, respectively. This classified the districts as moderately endemic, even after two MDAs. The other district, Murewha, slightly increased from 0% prevalence in MDA2–2%, thus remaining in low endemicity. Similarly, the Rushinga and Mberengwa districts slowly declined in prevalence during the first two MDAs, but significantly increased (P < 0.05) in prevalence and mean egg count compared to pre-MDA2. Post-MDA3, most districts significantly decreased in prevalence (P < 0.0001) and mean egg count (P < 0.05), the exceptions being three districts: Chiredzi, Mutoko, and Mount Darwin. In Chiredzi, there was a significant increase in prevalence (P < 0.0001) from 0% to 0.46%, but there was no significant increase in mean egg count (P = 0.318) with all infections categorised as light. Conversely, Mutoko had no significant reduction in prevalence (P = 0.509) only decreasing from 1.8% to 0.9%. However, there was a significant reduction in mean egg count (P < 0.05). In Mount Darwin, there was no significant change in mean egg count (2.27 to 2.33 eggs/10mL, P = 0.729) during MDA3, in addition to an increase of 2% (from 17% to 19%) of individuals in this population carrying heavy infections compared to the baseline survey for this MDA. The Mwenezi district significantly decreased in prevalence (P < 0.0001) but did not significantly decrease mean egg count (P = 0.894) after treatment, although the intensity was very low at 0.002 eggs/10mL. Overall, after three rounds of MDA, there were five (17.2%) districts in Zimbabwe still carrying infection, including Mount Darwin, Nkayi, Chiredzi, Mutoko, and Mwenezi, although all had a mean egg count <2.5 eggs/10mL.

***Changes in prevalence and egg counts pre and post MDA4****:* Twenty-two districts were surveyed at pre-MDA4, and only the Mount Darwin district (4.6%) was positive for *S. haematobium*. There was a significant decrease in prevalence and mean egg count compared to the pre-MDA1 survey, yet this district’s prevalence at pre-MDA4 was not significantly different from pre-MDA3, with an infection prevalence of 7.8% (P = 0.312). Post-MDA4, both prevalence (P < 0.0001) and mean egg count (P < 0.01) significantly reduced, with no active infections. Interestingly, despite the intervention program, both the Mutoko and UMP districts significantly increased prevalence (P < 0.0001) post-MDA4. Both districts increased by approximately 0.4% after PZQ treatment. However, neither significantly increased mean egg count and all were light infections.

***Changes in prevalence and egg counts pre and post MDA5****:* Pre-MDA5, only two of the twelve districts surveyed (16.7%) were positive for *S. haematobium*, and neither cleared infection post-MDA5. Chiredzi, a district with zero or low infection prevalence for the past two surveys, returned to pre-MDA2 moderate endemicity (~12.3%). Post-MDA5, Chiredzi had significantly reduced prevalence (P < 0.0001) to 0.9%, and in mean egg count (P < 0.0001) to 0.003 eggs/10mL (CI: 0-0.01). The second district positive for schistosome infection was UMP, with a low prevalence of 0.4%. After treatment, the prevalence did not change and remained at 0.4%, with no significant differences in mean egg count either (P = 0.318). Although the Mutoko district was not positive for *S. haematobium* pre-MDA5, the prevalence of infection significantly rose to 0.9% post-MDA5. However, the mean egg count did not significantly increase (P = 0.158), with an average of 0.003 eggs/10mL (CI: 0-0.01). All of the infections detected both pre- and post-MDA5 were designated as light infections, yet at the post-MDA5 survey the three districts described above were positive for infection.

**Fig 2:**
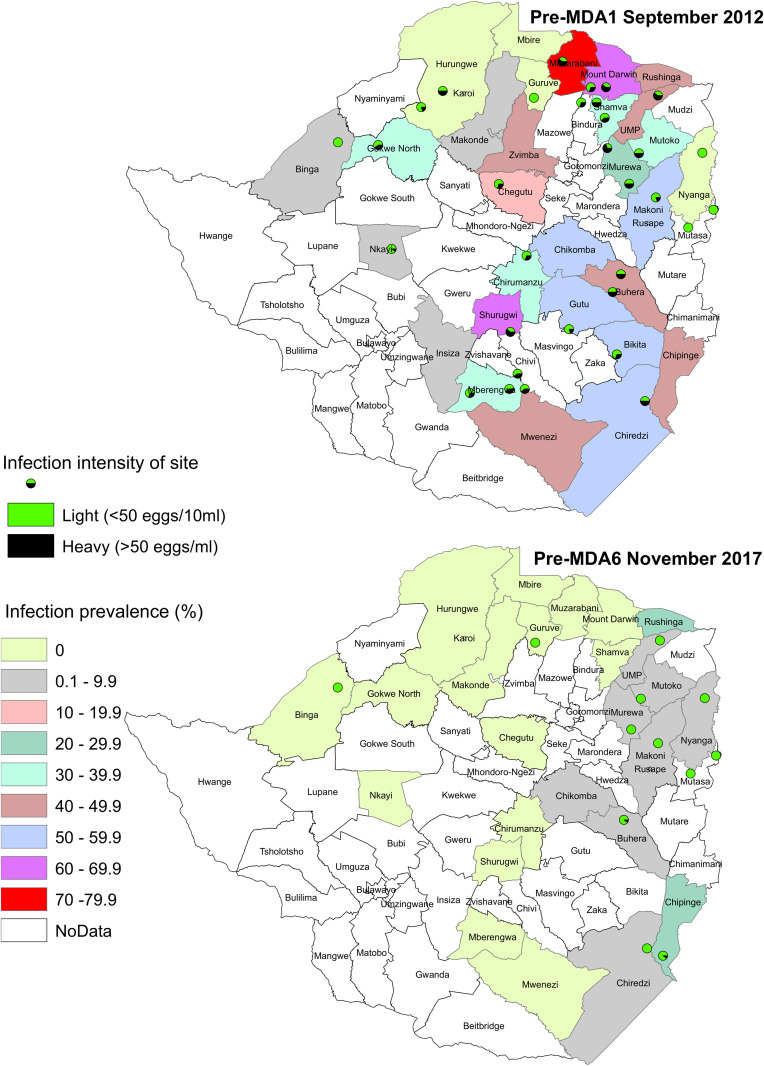
Choropleth maps depicting the geographical distribution of the prevalence of *S. haematobium* at the pre-MDA1 and pre-MDA6 surveys. The prevalence of each district is coloured to represent the prevalence (%) at that timepoint, with the sentinel site locations highlighted by a circular data point. These data points also represent the proportion of infection intensities at the survey, with the circular pie chart indicating what percentage of infections were light (light blue) or heavy (dark blue). The date at which the MDA was carried out is depicted on the right-hand side of the choropleth maps. Both maps were generated using QGIS, Version [3.22.2]. Zimbabwe National Statistics Agency (2022 ) Administrative boundaries shapefiles (district level), Zimbabwe National Statistics Agency (ZIMSTAT). Retrieved from https://data.humdata.org/dataset/cod-ab-zwe/resource/5ed39dd6-5124-4282-81cc-5295a3a976be (accessed 25 January 2025).

***Changes in prevalence and egg counts pre and post MDA6****:* As depicted in Fig 2, compared to the initial survey performed pre-MDA1, the overall prevalence of the country at pre-MDA6 was significantly decreased (P < 0.0001) at 1.47% (CI: 0.26-2.67%) and the mean egg count was also significantly reduced (P < 0.0001) to 1.39 eggs/10mL (CI: 0-3.9). Nine (37.5%) of the twenty-four districts investigated pre-MDA6 were positive for *S. haematobium*, with prevalence ranging from 0.2% to 11.2% across the infected districts. Only the Chipinge district had a prevalence >10%, classifying it as moderately endemic, and by post-MDA6 all districts had cleared infection. All districts had significantly reduced in prevalence (P < 0.0001) post-MDA6, and the only districts not significantly reduced in mean egg count were those with a low initial egg count (<0.3 eggs/10mL) at pre-MDA6. Overall, the national *S. haematobium* prevalence in Zimbabwe reduced the prevalence across all districts to 0% upon the last survey taken post-MDA6 in 2017.

### Measures of praziquantel efficacy in treating *S. haematobium* infection

The data on PZQ efficacy per district is collated in S3 Table and includes 49 measurements of PZQ efficacy. The CR’s and ERR’s are visualised on a map of Zimbabwe in S2A & S2B Fig, respectively, displaying the intensity of infection at every sentinel site. Those surveys which displayed reduced efficacy of PZQ (<100% CR/ERR) are displayed in Table 1.

Of those districts with reduced efficacy measures, 44.4% occurred in MDA1, 22.2% in MDA2, 11.1% in MDA3, and 22.2% in MDA5. As the efficacy measures assessed only paired samples that were positive for *S. haematobium*, further χ^2^ tests were conducted to compare each of the following variables for multiple associations: pre-MDA intensity, post-MDA intensity, post-MDA status (cleared or not cleared infection) for every village site and district. There was no significant association between the intensity of infection pre-MDA with infection intensity or post-MDA status. The variations in CR ranged from 0% to 99.1%, and ERR ranged from 4.2% to 99.9%. Three districts in that region show reduced CR’s and ERR’s: Mount Darwin, Rushinga and Shamva. The district of Mount Darwin (*n* = 126) had the highest district pre-mean egg count 80.13 eggs/10mL (CI: 65-95.27) and the largest percentage of heavy infections (44%) of any of the surveys in this analysis of reduced efficacy, with a CR of 68.3% and ERR of 91.7% in MDA1. An additional survey in Mount Darwin during MDA3 had low measures of PZQ efficacy, with a 22.2% CR and 28.6% ERR observed. At pre-MDA3, this district was 22% heavy infections, even after two rounds of MDAs, and was the only survey to remain above a mean egg count >15 egg/10mL at the follow-up survey.

Of the nine surveys with decreased efficacy, there was only one exception in which more than one village contributed to the efficacy survey: the Shamva district. This district consisted of two sites, where one village, Chihuri, had 100% CR/ERR and had an average light mean egg count infection of 47.56 eggs/10mL (CI: 22.49-72.63). The other village, Gono, had a CR/ERR of 96.6% and a heavy mean egg count infection at 85.58 eggs/10mL (CI: 62.7-108.45). The district had an average CR of 97.4% and ERR of 99.6%, with the initial intensity of infection remaining in the heavy category at 76.76 eggs/10mL (CI: 58.21-95.31). All other districts evaluated in this efficacy assessment significantly reduced in mean egg count upon PZQ treatment.

### *S. haematobium* hotspots in Zimbabwe

The impact of each round of MDA and the change in their prevalence after MDA4, 5 and 6 is presented in S4 Table. The analysis of potential PPHS was also conducted on a village level, as seen in S5 Table, to determine whether analysing the data by village would reveal any more precise mapping of hotspots. There were no differences in selecting hotspots between the village and district assessment. Approach A: Based on the lack of change in WHO prevalence category as described by the Kenyan and Tanzanian studies [20,21], Approach B: Based on prevalence ≥10% pre-MDA1 and remained ≥5% pre-MDA4/5/6, as described by a study in Zanzibar [22,23].

**Fig 3:**
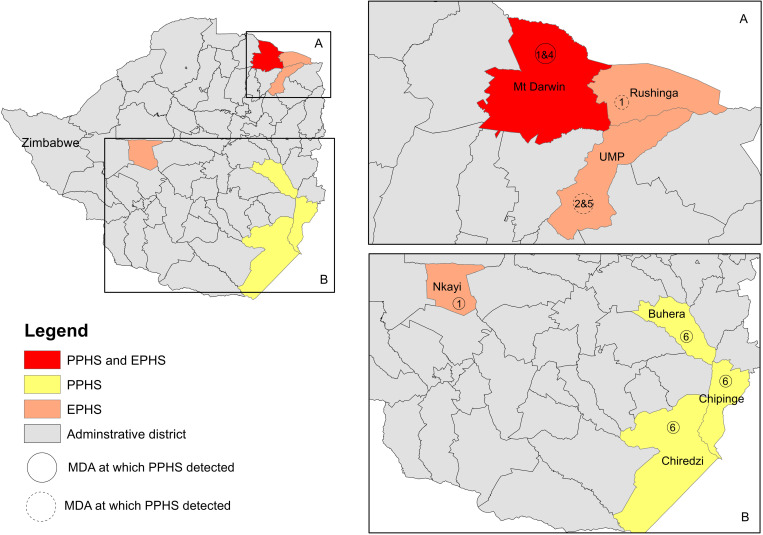
Map of Zimbabwe highlighting the districts detected as persistent hotspots of *S. haematobium* prevalence (PPHS), persistent hotspots of decreased praziquantel efficacy (EPHS), and districts of both PPHS and EPHS. The sentinel sites at which the surveys were conducted are labelled with a circle and the number in which the hotspot was detected. Zimbabwe National Statistics Agency (2022) Administrative boundaries shapefiles (district level), Zimbabwe National Statistics Agency (ZIMSTAT). Retrieved from https://data.humdata.org/dataset/cod-ab-zwe/resource/5ed39dd6-5124-4282-81cc-5295a3a976be (accessed 25 January 2025).

Based on Approach A, one PPHS was detected. No PPHS were detected at MDA4 on a district level. At MDA5, after four years of the control program, two districts had only declined one WHO category but were no designated as a PPHS. However, after five years of annual MDAs, Approach A concluded there was one PPHS of infection in the Chipinge district. With an initial prevalence of 48.8% it was classified as a moderately endemic district, and it remained moderately endemic at 11.2% at pre-MDA6, even after a significant reduction in prevalence since pre-MDA1. Furthermore, the district of Nyanga, although not fulfilling Approach A’s definition of a PPHS, it increased one WHO category with an increase of prevalence of 4.5% in MDA6, despite having 0% in all previous surveys. Approach B obtained additional PPHS, with four PPHS detected. Unlike Approach A (Table 2), it detected Mount Darwin and Chiredzi districts as potential PPHS at MDA4 and 5, respectively. Additionally, two districts were determined to be potential PPHS in MDA6. Buhera was identified in addition to Chipinge, with a pre-MDA6 prevalence of 7%. This approach also highlighted that Nyanga had increased one WHO category between pre-MDA1 and pre-MDA6, but as the prevalence was 4.5% it also did not fulfil the criteria to be designated as a PPHS using this approach either. Overall, by combining the approaches, we detected four PPHS during the six rounds of MDAs in Zimbabwe, with the PPHS presented in Fig 3.

### Persistent hotspots of decreased praziquantel efficacy (EPHS)

To determine if hotspots of persistent *S. haematobium* infection due to decreasing efficacy of PZQ occurred during the surveys we used Approach C, which focused primarily on CR < 70%. There were six surveys within four districts with a CR < 70%, with these studies taking place in MDA1, 2, 3 and 5 (see Fig 3). These were Nkayi (MDA1), UMP (MDA2/5), Rushinga (MDA2), and Mount Darwin (MDA1/3). The only survey found to have a CR < 70% and a heavy intensity of infection was Mount Darwin (MDA1). Notably, apart from Mount Darwin in MDA1, all the EPHS detected had a sample size *n* < 25 individuals.

### Potential hotspot, increased transmission and risk factors

**Fig 4:**
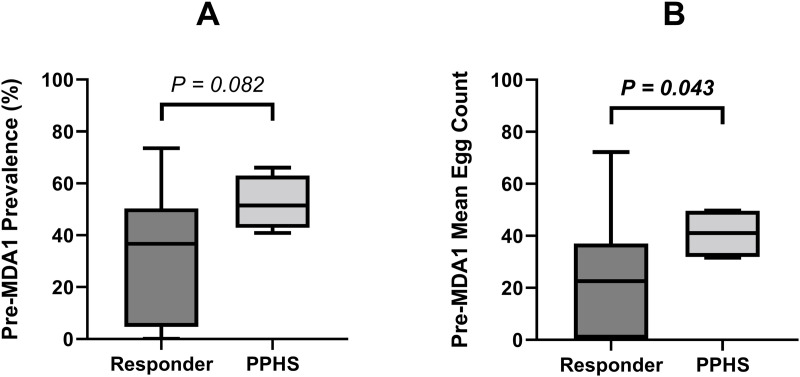
Box plots showing baseline prevalence and mean egg count at pre-MDA1 in districts that were responders compared to persistent hotspots of *S. haematobium* prevalence (PPHS). (A) Compares baseline prevalence, (B) Compares the baseline mean egg count.

To determine if there was a detectable difference between a district that responded to an MDA and a hotspot district, we assessed predictors of hotspot occurrence. This included increased transmission and risk factors (baseline prevalence, infection intensity, snail habitat, freshwater sources), and regime issues such as treatment coverage or frequency of PZQ treatment. This was to determine if we could predict a responder site versus a hotspot site based on relevant metadata. Beginning with PPHS risk factors, we analysed the pre-MDA1 data to determine if there was a detectable difference between a responder district and a hotspot district. Firstly, we investigated whether initial prevalence and mean egg count were related to the likelihood an area would respond well to the MDA or become a PPHS. Fig 4 shows the starting prevalence and mean egg counts of responder districts compared to those labelled as PPHS during this study using any PPHS approach.

The PPHS were identified using the approaches of classification described in detail in this study. The Mann–Whitney U-test P-values were indicated between the two groups. The median prevalence for PPHS was higher than in responder districts, as seen in Fig 4A, however no significant relationship was detected (P = 0.082). Conversely, as seen in Fig 4B, the median mean egg count of designated PPHS compared to those classed as responders was significantly higher (P = 0.043). Secondly, an analysis of the relationship between the known transmission factors for each dependent variable listed in S2A Table was conducted using separate stepwise linear regression models. There were no significant predictors for prevalence (Model 1 [R^2^ = 0.005, P = 0.744], Model 2 [R^2^ = 0.009, P = 0.581]) or mean egg count (Model 1 [R^2^ = 0.001, P = 0.948], Model 2 [R^2^ = 0.004, P = 0.778]) when using the following predictors: distance to the nearest waterway and the snail transmission scores from 1988 and 2012 (S2C Table). Additionally, neither the binary or ordinal logistic regression models displayed a significant relationship between the predictors and schistosomiasis status (Model 1 [NR^2^ = 0.008, P = 0.684], Model 2 [NR^2^ = 0.022, P = 0.363]) or WHO risk category (Model 1 [NR^2^ = 0.012, P = 0.528], Model 2 [NR^2^ = 0.22, P = 0.289]) to distinguish a responder compared to a PPHS (S2B Table).

**Fig 5:**
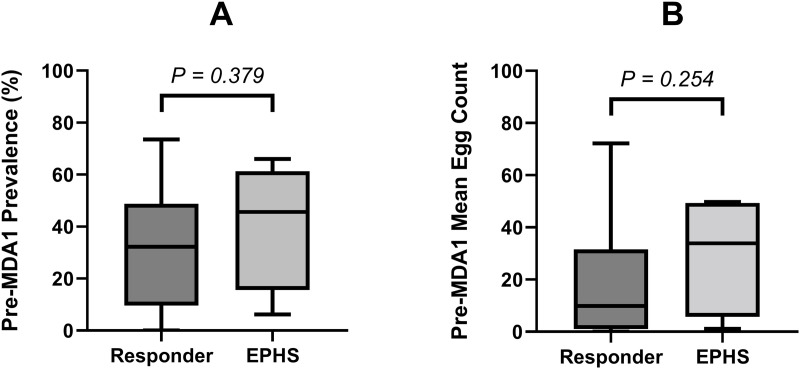
Box plots showing baseline prevalence and mean egg count at pre-MDA1 in districts that were responders compared to those that were persistent hotspots of decreasing praziquantel efficacy (EPHS). (A) Compares baseline prevalence, (B) Compares the baseline mean egg count. The EPHS were identified using the approaches of classification described in detail in this study. The Mann–Whitney U-test P-values are indicated between the two groups.

**Fig 6:**
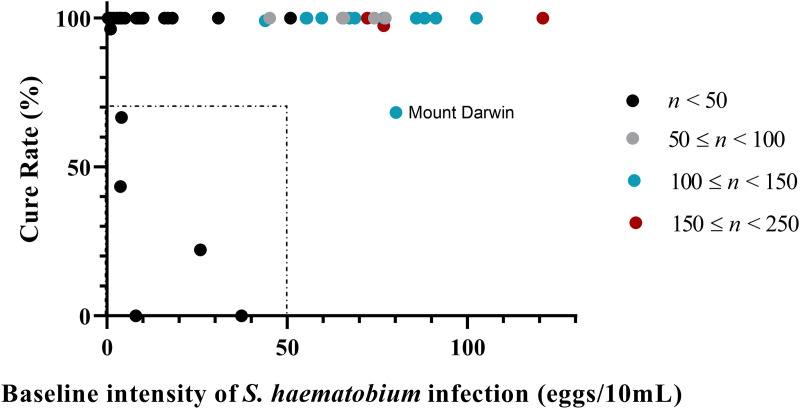
The relationship between pre-treatment intensity of *Schistosoma haematobium* infection and cure rate (CR) after praziquantel (PZQ) treatment during six years of annual mass drug administrations (MDAs) in Zimbabwe.

Moving on to the factors that may have a relationship with the efficacy of PZQ and may contribute towards EPHS, we first assessed pre-MDA1 prevalence and mean egg count. Fig 5 shows the starting prevalence and mean egg counts of responder districts compared to those categorised as an EPHS. The median prevalence and mean egg count for EPHS were slightly higher than responder districts, as seen in Fig 5A and 5B, however no significant relationship was detected (P = 0.379, P = 0.254). We then assessed whether pre-treatment intensity of infection was the cause of these low CRs, thus the mean egg count pre-MDA was plotted against the resultant CR in Fig 6.

The colour of the dots represents the different sample sizes (*n*) of the surveys: black, *n *< 50; grey, between 50–99; blue, between 100–149; red, between 150–249. The dotted area in which studies had a CR < 70%, and only light infections in the assessed population (<50 eggs/10mL). As shown in Fig 6, most surveys had 100% CR or very close to this value regardless of baseline infection intensity. χ2 tests found no significant association between intensity of infection in the pre-MDA survey with infection intensity or status (cleared or not cleared infection) at the post-MDA survey. The only EPHS found to have a CR < 70% and a heavy intensity of infection was Mount Darwin (MDA1). Although the lack of PZQ efficacy in Mount Darwin was expected to be because of these heavy infections, there was no significant association between infection intensity and status (P = 0.172). Five surveys had a CR < 70% that also carried light infections (Fig 6, dotted area), although all these surveys had a sample size *n *< 50. The EPHS were found in the districts of Nkayi (MDA1), UMP (MDA2/5), Rushinga (MDA2), and Mount Darwin (MDA3).

**Fig 7:**
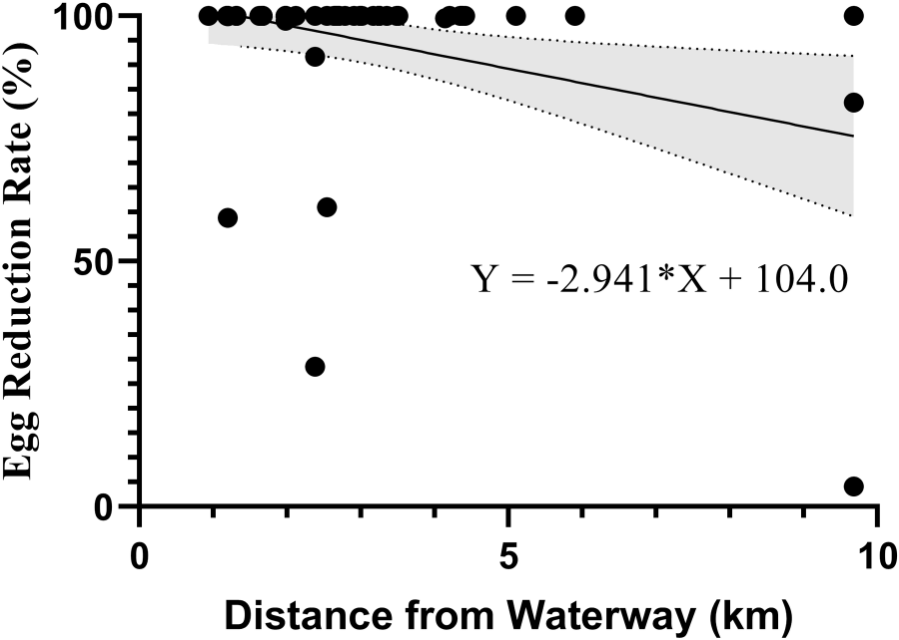
Linear regression of the relationship between the egg reduction rate and distance to the nearest waterway. This model was found to be significant (R^2^ = 0.106, P = 0.016). The shaded area represents the 95% confidence interval (CI).

To further analyse the relationship between known transmission and risk factors and EPHS detected in Zimbabwe, we used linear regression models (S2A Table) to assess predictors of CR and ERR. Models 3 and 4 indicated no significant relationship between the predictors and CR (Model 3 [R^2^ = 0.113, P = 0.2], Model 4 [R^2^ = 0.109, P = 0.219]) or ERR (Model 3 [R^2^ = 0.151, P = 0.085], Model 4 [R^2^ = 0.152, P = 0.084]) (S2C Table). Despite the models’ insignificance, the distance to the nearest waterway was a significant predictor. Consequently, Model 5 (S2C Table) was performed using only distance to the nearest waterway as a predictor for both CR and ERR, with no significant relationship with CR (Model 5 [R^2^ = 0.072, P = 0.0504]) detected. However, there was a significant relationship between ERR and distance to the nearest waterway of the sample sites (Model 5 [R^2^ = 0.106, P = 0.016]), as can be seen in Fig 7. Based on Model 5’s prediction, the further the sampling site from a potential transmission waterway, the lower the ERR.

### Coverage

**Fig 8:**
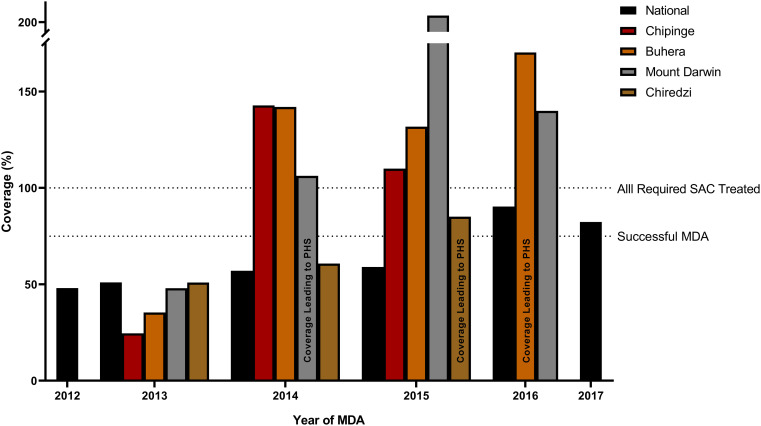
Coverage of the mass drug administration programs at the national and district level, for each detected persistent hotspots of *S. haematobium* prevalence (PPHS).

As determined by previous hotspot analyses (SCORE studies), sufficient coverage was defined at 50% of required SAC for the first round of MDA, then 75% for the remainder of the MDAs [29]. Therefore, we assessed the national and district coverage (when available) of each MDA where a PPHS of transmission was detected using Fig 8. A PPHS was identified in MDA4 in the district of Mount Darwin. At MDA3 preceding this identification, the national coverage was 57%, which is below the required threshold for a successful MDA. Yet, upon further investigation the district coverage was above 100%, meaning all eligible at-risk SAC received treatment. Furthermore, regarding the PPHS detected in MDA5 in Chiredzi, the national coverage in MDA4 was 59%. However, the district coverage was over 80%. Lastly, the national MDA coverage was 90.3% for both PPHS detected in MDA6; above the required ‘success’ thresholds.

### Seasonal treatments

To determine whether this change in treatment regimen was impacted by the month of administration, Table 3 monitored the impact of each MDA on the WHO risk category of the twelve districts followed in every MDA. We expected prevalence to gradually decrease upon every new round of PZQ treatment. However, compared to MDA2, four districts that increased in the WHO risk category, with two of those increasing by two WHO risk categories by MDA3. Furthermore, it had the highest number of increased WHO categories, with no districts exhibiting a decline in the WHO risk category (S6 Table). MDA3 also had the lowest number of districts with ‘no infections’, tying with MDA1.

**Table 3:** Comparison of the changes in WHO risk category compared to the baseline in the previous MDAs baseline category.

	Change in WHO Risk Category compared to baseline in the previous MDA						FE to MDA2–3
Month of Treatment	Declined 1	Declined 2	Increased 1	Increased 2	No Change	No Infection	P-Value
*October (MDA1–2)*	4	1	0	0	2	5	0.097
***January (MDA2–3)***	**0**	**0**	**2**	**2**	**3**	**5**	–
*November (MDA3–4)*	1	0	0	0	0	11	0.005
*November (MDA4–5)*	0	0	1	1	0	10	0.186
*November (MDA5–6)*	1	0	3	0	0	8	0.126

Significance was based on Fishers xact Test (FE) based on a 2x6 contingency table, assessing the change in MDAX-Y compared to MDA2–3. WHO: World health organization, MDA: Mass drug administration.

## Discussion

This study analysed the effectiveness of the national schistosomiasis control program in Zimbabwe, focusing on identifying persistent hotspots (PHS) and understanding factors influencing praziquantel (PZQ) efficacy across multiple rounds of MDA between 2012 and 2017. While the program substantially reduced *S. haematobium* prevalence nationally [8], variations in treatment response at village and district levels revealed areas of concern. Despite identifying several potential predictors such as proximity to water bodies, snail habitats, and infection intensity, only baseline (pre-MDA1) mean egg counts showed a significant association with PPHS classification. Although prevalence also appeared higher in PPHS areas, it did not reach statistical significance. These findings suggest that high baseline infection intensity may be an early indicator of PPHS, though the lack of a standardized definition for hotspots and variability across studies complicates classification and comparison [29]. The analysis also found that although reduced treatment frequency may be linked to persistent infection, the relationship was not statistically significant, possibly due to limited data and incomplete MDA coverage across the country. The study concludes that while coverage remains important, increasing treatment frequency and improving data completeness may be more critical for long-term schistosomiasis control in persistent hotspot regions [30].

While increasing the frequency of praziquantel (PZQ) treatment has shown potential to reduce the development of persistent schistosomiasis hotspots (PPHS), practical implementation in Zimbabwe presents significant challenges. These include logistical limitations such as transportation shortages, staff burden, drug availability, and population access constraints due to extreme weather [31]. Despite these barriers, the success of annual or biannual mass drug administration (MDA) programs for other neglected tropical diseases in similar settings suggests that such an approach could be feasible [17,32–34]. In this study, biennial treatment in Chipinge and Buhera was linked to PPHS emergence, supporting the argument for more frequent PZQ distribution in high-endemicity regions, regardless of baseline prevalence levels. From a public health perspective, increasing treatment frequency may offer significant benefits beyond reducing infection prevalence. PZQ has a good safety profile, making repeated treatments low risk for individuals [27]. Reducing schistosomiasis prevalence can lead to improved health-related quality of life (HRQoL), particularly in school-aged children (SAC), who are vulnerable to cognitive and educational impairments linked to infection [35]. These impairments can affect long-term socioeconomic outcomes, especially in resource-limited settings. Furthermore, timing of MDA implementation plays a crucial role; for example, delayed treatment during extended transmission seasons may reduce program effectiveness. Including preschool-aged children (PSAC) in future campaigns is also essential, particularly now that a paediatric PZQ formulation has received WHO prequalification [36,37].

Beyond pharmaceutical interventions, integrated strategies involving snail control and WASH are critical to breaking the parasite transmission cycle. Although this study did not find a statistically significant association between snail habitats and PPHS development, the lack of direct field data may have influenced this result. Snail control remains a recognized strategy in schistosomiasis elimination efforts. Equally important is the promotion of WASH interventions, which can reduce environmental contamination with schistosome eggs [38–42]. However, behavioral practices such as open defecation persist despite infrastructure availability, underlining the need for health education and community engagement. Although WASH data was not collected in this study, its integration with MDA and snail control could significantly enhance schistosomiasis prevention, especially in vulnerable PPHS regions [43]. This study identified several instances of decreased praziquantel (PZQ) efficacy, termed efficacy-persistent hotspots (EPHS), across multiple districts in Zimbabwe. In this study, suboptimal coverage may have limited the observed impact of MDA on egg clearance and reinfection rates, particularly in high-transmission settings. While PZQ may demonstrate good individual-level efficacy, the persistent transmission pressure due to untreated individuals can lead to rapid reinfection, thus as in our explanation, masking the drug efficacy at the population level. Future interventions should consider additional strategies to assess and improve both coverage and compliance, including follow-up verification and behavioral studies to understand reasons for non-compliance [44,45].

While some EPHS overlapped with prevalence-persistent hotspots (PPHS) [46], many did not, suggesting distinct underlying mechanisms. For example, districts like Buhera and Chipinge were PPHS without signs of reduced PZQ efficacy, implying environmental or regimen-based contributors rather than drug or host factors [47,48]. In our study, we observed increases in prevalence and/or egg intensity in some settings following each MDA. While we acknowledge the possibility of persistent low-level shedding in a few individuals, the pattern observed across multiple rounds and in larger cohorts suggests that this trend may reflect a combination of factors, including, incomplete parasite clearance (persistence) possibly due to suboptimal dosing or reduced drug efficacy [49–52]. Early reinfection in high-transmission areas with continued exposure to infested water, and variability in diagnostic sensitivity and daily egg output, which can influence observed prevalence and intensity measures, particularly in low-intensity settings. The timing of MDA also emerged as a possible factor, as treatments administered outside the peak transmission season may allow infections to persist and intensify. Additionally, the presence of heavy pre-treatment infections and delays in treatment, such as in Mount Darwin, the MDA3, may contribute to lower efficacy outcomes.

Further exploration revealed that while distance from freshwater sources had a weak negative correlation with ERR, this was likely due to infection intensity rather than true geographic isolation from transmission sites. Notably, no significant association was found between initial infection intensity and continued *S. haematobium* egg clearance, suggesting host-specific pharmacogenetic factors may affect PZQ metabolism and treatment outcomes [49–53]. The presence of juvenile schistosomes at the time of treatment could also contribute to apparent drug inefficacy, as PZQ is less effective against immature parasites [54,55]. Concerns about emerging resistance remain, though definitive field evidence is lacking [56,57]. Nevertheless, this underscores the importance of ongoing monitoring, further pharmacogenetic research, and consideration of future treatment alternatives or complementary control strategies [58]. The study faced limitations, including small sample sizes, missing data from many districts, and an inconsistent survey schedule, reducing the ability to draw broad conclusions about PPHS and EPHS. The absence of a standardized definition for PPHS further complicates comparisons across studies. Districts like Rushinga, which would qualify as PPHS under WHO criteria [59], were not classified as such using this study methods, highlighting the need for unified guidelines. Despite these constraints, pre-MDA1 infection intensity and prevalence emerged as possible predictors of PPHS. The study also emphasized the inadequacy of PZQ-alone strategies for long-term schistosomiasis control. To achieve the WHO goal of eliminating schistosomiasis as a public health problem by 2030 [60,61], MDA programs must be supplemented by interventions such as snail control, WASH improvements, and health education, additional strategies that address reinfection risks and improve long-term outcomes.

## Conclusion

Overall, this analysis evaluated the effectiveness of Zimbabwe’s national Mass Drug Administration (MDA) program for schistosomiasis over a six-year period (2012–2017), with a focus on identifying persistent and emerging hotspots (PPHS and EPHS) and assessing potential indicators of declining praziquantel (PZQ) efficacy. Through the monitoring of infection prevalence and treatment outcomes, we identified four prevalence-persistent hotspots that had received at least two annual MDAs. Importantly, we found that the method used to classify hotspots significantly influenced the number of PPHS identified, underscoring the need for a standardized definition to guide future surveillance and intervention planning. Moreover, nine instances of reduced PZQ efficacy were identified, with six meeting the threshold for classification as efficacy-persistent hotspots (EPHS), defined by cure rates below 70% across four districts. Contributing factors to both PPHS and EPHS were explored, including MDA coverage and frequency, timing of treatment, proximity to transmission sites, and host demographic and epidemiological characteristics. The findings emphasize the urgent need for a harmonized, evidence-based approach to hotspot classification, which will enhance consistency across schistosomiasis research and facilitate targeted, intensified interventions where needed. Ultimately, the data from this study provide critical insights to inform national control strategies. The information will support the Zimbabwe Ministry of Health and Child Care and its partners in developing cost-effective, evidence-based interventions that prioritize high-risk areas and work toward sustained reductions in disease burden and improvements in health-related quality of life.

## Supporting information

S1 Fig (A) The above choropleth maps depict the geographical distribution of the prevalence of *S. haematobium* at the pre- and post- surveys during rounds one, two and three of an annual mass drug administration (MDA) in Zimbabwe. Mapping units are district level boundaries [Zimbabwe National Statistics Agency (2022)]. Retrieved from https://data.humdata.org/dataset/cod-ab-zwe/resource/5ed39dd6-5124-4282-81cc-5295a3a976be (accessed 25 January 2025). (B) The above choropleth maps depict the geographical distribution of the prevalence of *S. haematobium* at the pre- and post- surveys during rounds four, five and six of an annual mass drug administration (MDA) in Zimbabwe. Mapping units are district level boundaries [Zimbabwe National Statistics Agency (2022)]. Retrieved from https://data.humdata.org/dataset/cod-ab-zwe/resource/5ed39dd6-5124-4282-81cc-5295a3a976be (accessed 25 January 2025).
(TIFF)

S2 Fig The above choropleth maps depict the geographical distribution of the cure rate (CR) of *S. haematobium* during six rounds of annual mass drug administrations (MDAs). Mapping units are district level boundaries [Zimbabwe National Statistics Agency (2022)]. Retrieved from https://data.humdata.org/dataset/cod-ab-zwe/resource/5ed39dd6-5124-4282-81cc-5295a3a976be (accessed 25 January 2025). (B)The above choropleth maps depict the geographical distribution of the egg reduction rate (ERR) of *S. haematobium* during six rounds of annual mass drug administrations (MDAs). Mapping units are district level boundaries [Zimbabwe National Statistics Agency (2022)]. Retrieved from https://data.humdata.org/dataset/cod-ab-zwe/resource/5ed39dd6-5124-4282-81cc-5295a3a976be (accessed 25 January 2025).
(TIFF)

S1 Table The transmission scores for both 1988 and 2012 of the suitability of the sample sites used in this study in Zimbabwe as a host snail habitat. Snail habitats were obtained from overlaying the spatial maps from Pedersen *et al.* [1,2] with the sample sites used in this study.
(DOCX)

S2 Table Model summary and predictors of each regression model. (DOCX)

S3 Table Prevalence, mean egg counts and proportion of light (< 50 eggs/10mL) to heavy (≥ 50 eggs/10mL) intensity of S. haematobium infection during six mass drug administrations (MDAs) during the years 2012–2017 in twenty-nine districts in Zimbabwe. (DOCX)

S4 Table Sample size, cure rate (CR), egg reduction rate (ERR), pre- and post-mean egg counts, and the proportion of light (< 50 eggs/10mL) to heavy (≥ 50 eggs/10mL) intensity of *S. haematobium* infection during six mass drug administrations (MDAs) during the years 2012–2017 in twenty-nine districts in Zimbabwe. (DOCX)

S5 Table The classification of districts based on their prevalence between multiple mass drug administration (MDA) time periods after the third MDA to determine whether persistent hotspots (PHS) could be detected in Zimbabwe using data from six years of annual treatment with PZQ. (DOCX)

S6 Table The classification of village sentinel sites based on their prevalence between multiple mass drug administration (MDA) time periods to determine whether persistent hotspots (PHS) could be detected in Zimbabwe using data from six years of annual treatment with PZQ. (DOCX)

S1 Text References. (DOCX)

S1 Data Hotspot excel datasets. (ZIP)
