# Supplementary material for: Identifying hotspots of S. haematobium infection following praziquantel treatment during multiple annual mass drug administration campaigns in Zimbabwe
Source: PLoS Negl Trop Dis. 2025 Sep 24;19(9):e0013546. doi: 10.1371/journal.pntd.0013546 (PMC12520393; doi:10.1371/journal.pntd.0013546)
Supplement: S1 Table — Snail habitats were obtained from overlaying the spatial maps from Pedersen et al. [1,2] with the sample sites used in this study. (DOCX) [file pntd.0013546.s003.docx]

| **District** | **Village** | **Transmission Suitability Score 1988** | **Transmission Suitability Score 2012** |
| --- | --- | --- | --- |
| UMP | Kafura | 0.54 | 0.77 |
| Bikita | Chitenderano | 0.54 | 0.38 |
| Gokwe North | Chireya | 0.31 | 0 |
| Hurungwe | Kajekache | 0.31 | 0.15 |
| Shurugwi | Newgato | 0.62 | 0.69 |
| Mazowe | Mbebi | 0.69 | 0.38 |
| Buhera | Masocha | 0.54 | 0.85 |
| Shamva | Gono | 0.54 | 0.77 |
| Shamva | Chihuri | 0.62 | 0.77 |
| Mberengwa | Zverenje | 0.69 | 0.77 |
| Nyanga | Chipataronga | 0.77 | 0.77 |
| Insiza | Insiza | 0.54 | 0.46 |
| Murehwa | Chingono | 0.69 | 0.85 |
| Mberengwa | Mukwakwe | 0.54 | 0.69 |
| Mutoko | Chimukopa | 0.69 | 0.77 |
| Zvimba | Chomutamba | 0.69 | 0.77 |
| Binga | Siabuwa | 0.38 | 0 |
| Murehwa | Chiguri | 0.69 | 0.69 |
| Chirumanzu | Gambiza | 0.77 | 0.62 |
| Makoni | Bandanyenje | 0.69 | 0.85 |
| Chikomba | Nhidza | 0.77 | 0.62 |
| Chegutu | Gadzema | 0.54 | 0.54 |
| Nkayi | Gonye | 0.46 | 0.31 |
| Mt Darwin | Bemberi | 0.69 | 0.85 |
| Makonde | Kanyaga | 0.46 | 0.31 |
| Chiredzi | Mareya | 0.46 | 0.54 |
| Mwenezi | Ruzongwe | 0.54 | 0.69 |
| Gutu | Mutendeure | 0.77 | 0.62 |
| Mutoko | Kushinga | 0.69 | 0.85 |
| Chipinge | Chitepo | 0.38 | 0.77 |
| Rushinga | Mazowe Bridge | 0.54 | 0.62 |
| Muzarabani | Muzarabani | 0.31 | 0.38 |
| Mberengwa | Nhenga | 0.46 | 0.54 |
| Guruve | Nyanhunzi | 0.69 | 0.69 |
| Hurungwe | Dandawa | 0.31 | 0.15 |
