# Supplementary material for: Identifying hotspots of S. haematobium infection following praziquantel treatment during multiple annual mass drug administration campaigns in Zimbabwe
Source: PLoS Negl Trop Dis. 2025 Sep 24;19(9):e0013546. doi: 10.1371/journal.pntd.0013546 (PMC12520393; doi:10.1371/journal.pntd.0013546)
Supplement: S2 Table — (DOCX) [file pntd.0013546.s004.docx]

S2A

| **Regression Model** | ***Predictors*** | | | | |
| --- | --- | --- | --- | --- | --- |
|  |  |  |  |  |  |
|  | **Distance to Body of Water** | **Transmission Score** | | **Baseline** | |
|  |  | **1988** | **2012** | **Prevalence** | **Mean Egg Count** |
| 1 | x | x |  |  |  |
| 2 | x |  | x |  |  |
| 3 | x | x |  | x | x |
| 4 | x |  | x | x | x |
| 5 | x |  |  |  |  |

S2B

| **Logistic Regression Model** | **Method** | **Dependent Variable** | **Predictors** | | | **Model Parameters** | | |
| --- | --- | --- | --- | --- | --- | --- | --- | --- |
|  |  |  | **Transmission Score** | | **Distance to Body of Water** |  |  |  |
|  |  |  | **1988** | **2012** |  | -2 log likelihood | Nagelkerke R^2^ | P |
| 1 | Binary | Schistosomiasis Status | B=0.497, Wald=0.132, p=0.717 |  | B=-0.77, Wald=0.65, p=0.42 | 166.76 | 0.008 | 0.684 |
|  | Ordinal | WHO Risk Category | Estimate=0.393, Wald=0.091, p=0.76 |  | Estimate=-0.101, Wald=1.13, p=0.288 | 167.27 | 0.012 | 0.528 |
| 2 | Binary | Schistosomiasis Status |  | B=1.019, Wald=1.357, p=0.244 | B=-0.81, Wald=0.721, p=0.396 | 165.49 | 0.022 | 0.363 |
|  | Ordinal | WHO Risk Category |  | Estimate=0.93, Wald=1.219, p=0.27 | Estimate=-0.108, Wald=1.295, p=0.255 | 167.27 | 0.22 | 0.289 |

S2C

| **Linear Regression Model** | **Dependent Variable** | ***Predictors*** | | | | | **Model Parameters** | | |
| --- | --- | --- | --- | --- | --- | --- | --- | --- | --- |
|  |  | **Transmission Score** | | **Distance to Body of Water** | **Baseline Prevalence** | **Baseline Mean Egg Count** |  |  |  |
|  |  | **1988** | **2012** |  |  |  | R^2^ | **F** | **Sig** |
| 1 | Prevalence | B=1.97, p=0.88 |  | B=-0.676, p=0.446 |  |  | 0.005 | 0.297 | 0.744 |
|  | Mean Egg Count | B=-2.351, p=0.829 |  | B=-1.68, p=0.819 |  |  | 0.001 | 0.054 | 0.948 |
| 2 | Prevalence |  | B=5.763, p=0.473 | B=-0.697, p=0.43 |  |  | 0.009 | 0.545 | 0.581 |
|  | Mean Egg Count |  | B=4.427, p=0.508 | B=-0.205, p=0.781 |  |  | 0.004 | 0.251 | 0.778 |
| 3 | Cure Rate | B=12.651, p=0.617 |  | B=-3.343, p=0.044 | B=-0.33, p=0.901 | B=0.249, p=0.385 | 0.113 | 1.561 | 0.2 |
|  | Egg Reduction Rate | B=6.063, p=0.748 |  | B=-3.035, p=0.015 | B=-0.004, p=0.985 | B=0.183, p=0.392 | 0.151 | 2.179 | 0.085 |
| 4 | Cure Rate |  | B=1.613, p=0.920 | B=-3.2743, p=0.05 | B=-0.21, p=0.935 | B=0.233, p=0.415 | 0.109 | 1.493 | 0.219 |
|  | Egg Reduction Rate |  | B=-4.365, p=0.714 | B=-2.9233, p=0.02 | B=0, p=0.99 | B=0.178, p=0.403 | 0.152 | 2.188 | 0.084 |
| 5 | Cure Rate |  |  | B=-3.1711, p=0.05 |  |  | 0.072 | 4.011 | 0.0504 |
|  | **Egg Reduction Rate** |  |  | **B=-2.941, p=0.016** |  |  | **0.106** | **6.154** | **0.016** |
