# Supplementary material for: Identifying hotspots of S. haematobium infection following praziquantel treatment during multiple annual mass drug administration campaigns in Zimbabwe
Source: PLoS Negl Trop Dis. 2025 Sep 24;19(9):e0013546. doi: 10.1371/journal.pntd.0013546 (PMC12520393; doi:10.1371/journal.pntd.0013546)
Supplement: S3 Table — (DOCX) [file pntd.0013546.s005.docx]

|  | | **Province** | **Manicaland** | | | | | |  |  |
| --- | --- | --- | --- | --- | --- | --- | --- | --- | --- | --- |
|  |  | **District** | **Bikita** | **Buhera** | **Chipinge** | | **Makoni** | **Nyanga** |  |  |
| **MDA1** | **Pre-** | **Prevalence (n)** | 58.46 (195) ^[A]^ | 40.91 (242) ^[A], [D]iv, vi^ | 48.8 (250) ^[A], [D]iv, vi^ | | 52.79 (233) ^[A], [D]ii, iii, iv, v, vi^ | 0 (397) ^[D]vi^ |  |  |
|  |  | **Mean Egg Count (95% CI)** | 34.31 (24.56-44.06) ^[A]^ | 31.57 (21.8-41.35) ^[A], [D]vi^ | 34.31 (24.56-44.06) ^[A], [D]vi^ | | 29.27 (20.06-38.48) ^[A]^ | 0 (0-0) |  |  |
|  |  | **Light:Heavy Infections (%)** | 71:29 | 58:42 | 62:38 | | 73:27 | 0:0 |  |  |
|  | **Post-** | **Prevalence (n)** | 0 (195) | 0 (242) | 0 (250) | | 0 (233) | 0 (398) |  |  |
|  |  | **Mean Egg Count (95% CI)** | 0 (0-0) ^[A]^ | 0 (0-0) ^[A]^ | 0 (0-0) ^[A]^ | | 0 (0-0) ^[A]^ | 0 (0-0) |  |  |
|  |  | **Light:Heavy Infections (%)** | 0:0 | 0:0 | 0:0 | | 0:0 | 0:0 |  |  |
| **MDA2** | **Pre-** | **Prevalence (n)** | - | - | - | | 0 (233) ^[D]i, vi^ | - |  |  |
|  |  | **Mean Egg Count (95% CI)** | - | - | - | | 0 (0-0) | - |  |  |
|  |  | **Light:Heavy Infections (%)** | - | - | - | | 0:0 | - |  |  |
|  | **Post-** | **Prevalence (n)** | - | - | - | | 0 (191) | - |  |  |
|  |  | **Mean Egg Count (95% CI)** | - | - | - | | 0 (0-0) | - |  |  |
|  |  | **Light:Heavy Infections (%)** | - | - | - | | 0:0 | - |  |  |
| **MDA3** | **Pre-** | **Prevalence (n)** | - | - | - | | 0 (233) ^[D]i, vi^ | - |  |  |
|  |  | **Mean Egg Count (95% CI)** | - | - | - | | 0 (0-0) | - |  |  |
|  |  | **Light:Heavy Infections (%)** | - | - | - | | 0:0 | - |  |  |
|  | **Post-** | **Prevalence (n)** | - | - | - | | 0 (233) | - |  |  |
|  |  | **Mean Egg Count (95% CI)** | - | - | - | | 0 (0-0) | - |  |  |
|  |  | **Light:Heavy Infections (%)** | - | - | - | | 0:0 | - |  |  |
| **MDA4** | **Pre-** | **Prevalence (n)** | - | 0 (242) ^[D]i, vi^ | 0 (250) ^[D]i, vi^ | | 0 (233) ^[D]i, vi^ | 0 (398) |  |  |
|  |  | **Mean Egg Count (95% CI)** | - | 0 (0-0) | 0 (0-0) | | 0 (0-0) | 0 (0-0) |  |  |
|  |  | **Light:Heavy Infections (%)** | - | 0:0 | 0:0 | | 0:0 | 0:0 |  |  |
|  | **Post-** | **Prevalence (n)** | - | 0 (242) | 0 (250) | | 0 (233) | 0 (398) |  |  |
|  |  | **Mean Egg Count (95% CI)** | - | 0 (0-0) | 0 (0-0) | | 0 (0-0) | 0 (0-0) |  |  |
|  |  | **Light:Heavy Infections (%)** | - | 0:0 | 0:0 | | 0:0 | 0:0 |  |  |
| **MDA5** | **Pre-** | **Prevalence (n)** | - | - | - | | 0 (233) ^[D]i, vi^ | - |  |  |
|  |  | **Mean Egg Count (95% CI)** | - | - | - | | 0 (0-0) | - |  |  |
|  |  | **Light:Heavy Infections (%)** | - | - | - | | 0:0 | - |  |  |
|  | **Post-** | **Prevalence (n)** | - | - | - | | 0 (192) | - |  |  |
|  |  | **Mean Egg Count (95% CI)** | - | - | - | | 0 (0-0) | - |  |  |
|  |  | **Light:Heavy Infections (%)** | - | - | - | | 0:0 | - |  |  |
| **MDA6** | **Pre-** | **Prevalence (n)** | - | 7.03 (242) ^[A], [D]iv^ | 11.2 (250) ^[A], [D]i, iv^ | | 3.86 (233) ^[A], [D]i, ii, iii, vi, v^ | 4.52 (398) ^[A], [D]i^ |  |  |
|  |  | **Mean Egg Count (95% CI)** | - | 1.19 (0.08-2.3) ^[A], [D]i^ | 0.91 (0.37-1.45) ^[A], [D]i^ | | 0.62 (0.05-1.18) ^[A]^ | 0.43 (0.14-0.72) ^[A]^ |  |  |
|  |  | **Light:Heavy Infections (%)** | - | 88:12 | 100:0 | | 100:0 | 100:0 |  |  |
|  | **Post-** | **Prevalence (n)** | 0 (195) | 0 (242) ^[A]^ | 0 (250) ^[A]^ | | 0 (233) ^[A]^ | 0 (398) ^[A]^ |  |  |
|  |  | **Mean Egg Count (95% CI)** | 0 (0-0) | 0 (0-0) ^[A]^ | 0 (0-0) ^[A]^ | | 0 (0-0) ^[A]^ | 0 (0-0) ^[A]^ |  |  |
|  |  | **Light:Heavy Infections (%)** | 0:0 | 0:0 | 0:0 | | 0:0 | 0:0 |  |  |
|  | | | | | | | | |  |  |
|  | | **Province** | **Mash West** | | | | | **Mashonaland Central** |  |  |
|  |  | **District** | **Chegutu** | **Hurungwe** | **Makonde** | | **Zvimba** | **Guruve** |  |  |
| **MDA1** | **Pre-** | **Prevalence (n)** | 15.73 (267) [A], [D]iv, vi | 0 (439) | 9.6 (241) [A], [D]iv, vi | | 46.18 (248) [A] | 0 (179) |  |  |
|  |  | **Mean Egg Count (95% CI)** | 8.01 (3.9-12.11) [A] | 0 (0-0) | 1 (0.33-1.68) [A] | | 39.6 (27.33-51.86) [A] | 0 (0-0) |  |  |
|  |  | **Light:Heavy Infections (%)** | 71:29 x | 0:0 | 96:4 x | | 59:41 x | 0:0 |  |  |
|  | **Post-** | **Prevalence (n)** | 0 (267) | 0 (439) | 0 (250) [A] | | 0 (248) [A] | 0 (179) |  |  |
|  |  | **Mean Egg Count (95% CI)** | 0 (0-0) [A] | 0 (0-0) [A] | 0 (0-0) [A] | | 0 (0-0) [A] | 0 (0-0) |  |  |
|  |  | **Light:Heavy Infections (%)** | 0:0 | 0:0 | 0:0 | | 0:0 | 0:0 |  |  |
| **MDA2** | **Pre-** | **Prevalence (n)** | - | - | - | | - | - |  |  |
|  |  | **Mean Egg Count (95% CI)** | - | - | - | | - | - |  |  |
|  |  | **Light:Heavy Infections (%)** | - | - | - | | - | - |  |  |
|  | **Post-** | **Prevalence (n)** | - | - | - | | - | - |  |  |
|  |  | **Mean Egg Count (95% CI)** | - | - | - | | - | - |  |  |
|  |  | **Light:Heavy Infections (%)** | - | - | - | | - | - |  |  |
| **MDA3** | **Pre-** | **Prevalence (n)** | - | - | - | | - | - |  |  |
|  |  | **Mean Egg Count (95% CI)** | - | - | - | | - | - |  |  |
|  |  | **Light:Heavy Infections (%)** | - | - | - | | - | - |  |  |
|  | **Post-** | **Prevalence (n)** | - | - | - | | - | - |  |  |
|  |  | **Mean Egg Count (95% CI)** | - | - | - | | - | - |  |  |
|  |  | **Light:Heavy Infections (%)** | - | - | - | | - | - |  |  |
| **MDA4** | **Pre-** | **Prevalence (n)** | 0 (267) ^[D]i^ | 0 (439) | 0 (249) ^[D]i^ | | - | 0 (179) |  |  |
|  |  | **Mean Egg Count (95% CI)** | 0 (0-0) | 0 (0-0) | 0 (0-0) | | - | 0 (0-0) |  |  |
|  |  | **Light:Heavy Infections (%)** | 0:0 | 0:0 | 0:0 | | - | 0:0 |  |  |
|  | **Post-** | **Prevalence (n)** | 0 (267) | 0 (439) | 0 (249) | | - | 0 (179) |  |  |
|  |  | **Mean Egg Count (95% CI)** | 0 (0-0) | 0 (0-0) | 0 (0-0) | | - | 0 (0-0) |  |  |
|  |  | **Light:Heavy Infections (%)** | 0:0 | 0:0 | 0:0 | | - | 0:0 |  |  |
| **MDA5** | **Pre-** | **Prevalence (n)** | - | - | - | | - | - |  |  |
|  |  | **Mean Egg Count (95% CI)** | - | - | - | | - | - |  |  |
|  |  | **Light:Heavy Infections (%)** | - | - | - | | - | - |  |  |
|  | **Post-** | **Prevalence (n)** | - | - | - | | - | - |  |  |
|  |  | **Mean Egg Count (95% CI)** | - | - | - | | - | - |  |  |
|  |  | **Light:Heavy Infections (%)** | - | - | - | | - | - |  |  |
| **MDA6** | **Pre-** | **Prevalence (n)** | 0 (267) ^[D]i^ | 0 (439) | 0 (249) ^[D]vi^ | | - | 0 (179) |  |  |
|  |  | **Mean Egg Count (95% CI)** | 0 (0-0) | 0 (0-0) | 0 (0-0) | | - | 0 (0-0) |  |  |
|  |  | **Light:Heavy Infections (%)** | 0:0 | 0:0 | 0:0 | | - | 0:0 |  |  |
|  | **Post-** | **Prevalence (n)** | 0 (267) | 0 (439) | 0 (250) | | 0 (248) | 0 (179) |  |  |
|  |  | **Mean Egg Count (95% CI)** | 0 (0-0) | 0 (0-0) | 0 (0-0) | | 0 (0-0) | 0 (0-0) |  |  |
|  |  | **Light:Heavy Infections (%)** | 0:0 | 0:0 | 0:0 | | 0:0 | 0:0 |  |  |
|  | |  |  | | | | | |  |  |
|  | | **Province** | **Mashonaland Central** | | | | | |  |  |
|  |  | **District** | **Mazowe** | **Mt Darwin** | **Muzarabani** | | **Rushinga** | **Shamva** |  |  |
| **MDA1** | **Pre-** | **Prevalence (n)** | 0 (186) | 66.02 (206) ^[C], [D]ii, iii, iv, v, vi^ | 73.58 (299) ^[A], [D]ii, iii, iv, v, vi^ | | 44 (250) ^[A], [D]ii, iii, iv, v, vi^ | 32.19 (612) ^[A], [D]iv, vi^ |  |  |
|  |  | **Mean Egg Count (95% CI)** | 0 (0-0) | 49.69 (39.08-60.31) ^[A], [D]iii,[D]iv^ | 88.93 (75.33-102.53) ^[A], [D]iii^ | | 19.31 (12.98-25.64) ^[A], [D]ii, [D]iii, [D]vi^ | 24.36 (17.87-30.86) ^[A]^ |  |  |
|  |  | **Light:Heavy Infections (%)** | 0:0 | 56:44 | 34:66 | | 81:19 | 67:33 |  |  |
|  | **Post-** | **Prevalence (n)** | 0 (186) | 24.27 (206) ^[C], [E]iii, iv, v, vi^ | 0 (320) ^[A], [E]ii^ | | 1.2 (250) ^[A], [E]ii, [F]iii, iv, v, vi^ | 0.89 (1118) ^[A], [E]vi, [G]iv^ |  |  |
|  |  | **Mean Egg Count (95% CI)** | 0 (0-0) | 4.96 (3.21-6.71) ^[A], [E]iii^ | 0 (0-0) ^[A]^ | | 0.01 (0-0.01) ^[A], [E]ii, [G]iv^ | 0.35 (0.05-0.65) ^[A]^ |  |  |
|  |  | **Light:Heavy Infections (%)** | 0:0 | 95:5 | 0:0 | | 100:0 | 90:10 |  |  |
| **MDA2** | **Pre-** | **Prevalence (n)** | - | 0 (206) ^[D]I, iii, iv^ | 0 (297) ^[B], [D]i, iii^ | | 21.6 (250) ^[A], [D]I, iii, iv, v, vi^ | - |  |  |
|  |  | **Mean Egg Count (95% CI)** | - | 0 (0-0) | 0 (0-0) ^[B]^ | | 0.75 (0.49-1) ^[C], [D]i, [D]iii, [D]vi^ | - |  |  |
|  |  | **Light:Heavy Infections (%)** | - | 0:0 | 0:0 | | 100:0 | - |  |  |
|  | **Post-** | **Prevalence (n)** | - | - | 6.06 (298) ^[B], [E]iii, iv, v, vi^ | | 17.6 (250) ^[A], [E]i, iii, iv, v, vi^ | - |  |  |
|  |  | **Mean Egg Count (95% CI)** | - | - | 0.06 (0.02-0.1) ^[B] [F]iii^ | | 0.98 (0.62-1.35) ^[C], [E]i, [E]iv^ | - |  |  |
|  |  | **Light:Heavy Infections (%)** | - | - | 100:0 | | 100:0 | - |  |  |
| **MDA3** | **Pre-** | **Prevalence (n)** | - | 11.17 (206) [A], [D]ii, v, vi, [F]iv | 10.37 (299) ^[A], [D]i, ii, iv, v, vi^ | | 32 (250) [A], [D]i, ii, iv, v, vi | - |  |  |
|  |  | **Mean Egg Count (95% CI)** | - | 2.27 (0.89-3.64) [C], [D]i, [F]iv | 0.05 (0.03-0.07) [A], [D]I, [F]ii | | 2.99 (2.02-3.97) [A],[D]i, [D]ii, [D]vi | - |  |  |
|  |  | **Light:Heavy Infections (%)** | - | 83:17 | 100:0 | | 99:1 | - |  |  |
|  | **Post-** | **Prevalence (n)** | - | 0.49 (206) ^[A], [E]i, iv, v, vi^ | 0 (298) ^[E]ii^ | | 0 (250) ^[A], [F]i, ii, iv^ | - |  |  |
|  |  | **Mean Egg Count (95% CI)** | - | 2.33 (0.93-3.73) ^[C], [E]i^ | 0 (0-0) | | 0 (0-0) ^[A], [E]ii, [G]i^ | - |  |  |
|  |  | **Light:Heavy Infections (%)** | - | 81:19 | 0:0 | | 0:0 | - |  |  |
| **MDA4** | **Pre-** | **Prevalence (n)** | 0 (186) | 7.77 (206) ^[A], [D]I, ii, v, vi, [F]iii^ | 0 (298) ^[D]i, iii^ | | 0 (250) ^[B], [D]I, ii, iii, [F]vi^ | 0 (218) ^[D]i^ |  |  |
|  |  | **Mean Egg Count (95% CI)** | 0 (0-0) | 1.41 (0.47-2.35) ^[A], [D]i^ | 0 (0-0) | | 0 (0-0) ^[C]^ | 0 (0-0) |  |  |
|  |  | **Light:Heavy Infections (%)** | 0:0 | 88:13 | 0:0 | | 0:0 | 0:0 |  |  |
|  | **Post-** | **Prevalence (n)** | 0 (186) | 0 (206) ^[A], [E]i, iii^ | 0 (298) ^[E]ii^ | | 1.2 (250) ^[B], [E]ii, [F]I, iii, v, vi^ | 0 (218) ^[G]i^ |  |  |
|  |  | **Mean Egg Count (95% CI)** | 0 (0-0) | 0 (0-0) ^[A]^ | 0 (0-0) | | 0.004 (0-0.01) ^[C], [E]ii, [G]i^ | 0 (0-0) |  |  |
|  |  | **Light:Heavy Infections (%)** | 0:0 | 0:0 | 0:0 | | 100:0 | 0:0 |  |  |
| **MDA5** | **Pre-** | **Prevalence (n)** | - | 0 (216) ^[D]iii, iv^ | 0 (298) ^[D]i, iii^ | | 0 (250) ^[D]i, ii, iii, [F]vi^ | - |  |  |
|  |  | **Mean Egg Count (95% CI)** | - | 0 (0-0) | 0 (0-0) | | 0 (0-0) | - |  |  |
|  |  | **Light:Heavy Infections (%)** | - | 0:0 | 0:0 | | 0:0 | - |  |  |
|  | **Post-** | **Prevalence (n)** | - | 0 (196) ^[E]i, iii^ | 0 (298) ^[E]ii^ | | 0 (250) ^[E]ii, [F]I, iv^ | - |  |  |
|  |  | **Mean Egg Count (95% CI)** | - | 0 (0-0) | 0 (0-0) | | 0 (0-0) | - |  |  |
|  |  | **Light:Heavy Infections (%)** | - | 0:0 | 0:0 | | 0:0 | - |  |  |
| **MDA6** | **Pre-** | **Prevalence (n)** | 0 (186) | 0 (206) ^[D]iii, iv^ | 0 (298) ^[D]i, iii^ | | 0.4 (250) ^[A], [D]ii, iii, vi, [F]iv, v^ | 0 (218) ^[D]i^ |  |  |
|  |  | **Mean Egg Count (95% CI)** | 0 (0-0) | 0 (0-0) | 0 (0-0) | | 0.001 (0-0.33) ^[C], [D]i, [D]ii, [D]iii^ | 0 (0-0) |  |  |
|  |  | **Light:Heavy Infections (%)** | 0:0 | 0:0 | 0:0 | | 100:0 | 0:0 |  |  |
|  | **Post-** | **Prevalence (n)** | 0 (186) | 0 (206) ^[E]i, iii^ | 0 (298) ^[E]ii^ | | 0 (250) ^[A], [E]ii, [F]i, iv^ | 0 (468) ^[E]i^ |  |  |
|  |  | **Mean Egg Count (95% CI)** | 0 (0-0) | 0 (0-0) | 0 (0-0) | | 0 (0-0) ^[C]^ | 0 (0-0) |  |  |
|  |  | **Light:Heavy Infections (%)** | 0:0 | 0:0 | 0:0 | | 0:0 | 0:0 |  |  |
|  | | | | | | | | |  |  |
|  | | **Province** | **Mashonaland East** | | | | | **Masvingo** |  |  |
|  |  | **District** | **Chikomba** | **Murehwa** | **Mutoko** | | **UMP** | **Chiredzi** |  |  |
| **MDA1** | **Pre-** | **Prevalence (n)** | 50.4 (250) ^[A], [D]iv, vi^ | 23.46 (422) ^[A], [D]ii, iii, iv, v, vi^ | 30.57 (422) ^[A], [D]ii, iii, iv, v, vi^ | | 47.2 (250) ^[A], [D]ii, iii, iv, v, vi^ | 54.13 (218) ^[A], [D]ii, iii, iv, v, vi^ |  |  |
|  |  | **Mean Egg Count (95% CI)** | 27.82 (20.27-35.36) ^[A], [D]vi^ | 17.4 (11.83-22.96) ^[A], [D]iii, [D]vi,^ | 21 (15.31-26.69) ^[A], [D]ii, [D]iii, [D]vi^ | | 48.37 (36.07-60.66) ^[A], [D]ii, [D]v^ | 49.37 (36.02-62.73) ^[A], [D]ii, [D]v, [D]vi^ |  |  |
|  |  | **Light:Heavy Infections (%)** | 69:31 | 60:40 | 64:36 | | 43:57 | 48:52 |  |  |
|  | **Post-** | **Prevalence (n)** | 0 (250) ^[A]^ | 0 (422) ^[A]^ | 0 (422) ^[A], [G]iii, iv, v^ | | 0 (250) ^[A], [E]ii, [G]iv, v^ | 0 (218) ^[A], [F]iii, v^ |  |  |
|  |  | **Mean Egg Count (95% CI)** | 0 (0-0) ^[A]^ | 0 (0-0) ^[A]^ | 0 (0-0) ^[A]^ | | 0 (0-0) ^[A]^ | 0 (0-0) ^[A]^ |  |  |
|  |  | **Light:Heavy Infections (%)** | 0:0 | 0:0 | 0:0 | | 0:0 | 0:0 |  |  |
| **MDA2** | **Pre-** | **Prevalence (n)** | - | 0 (198) ^[D]i, vi, [F]iii^ | 4.02 (224) ^[A], [D]i, iv, v, vi, [F]iii^ | | 6.8 (250) ^[B], [D]i, iii, iv, v, vi^ | 12.39 (218) ^[A], [D]i, iii, iv, v, vi^ |  |  |
|  |  | **Mean Egg Count (95% CI)** | - | 0 (0-0) | 0.04 (0.01-0.07) ^[A], [D]i, [F]iii, [F]vi^ | | 0.17 (0.05-0.29) ^[C], [D]i, [D]v^ | 0.43 (0.2-0.66) ^[A], [D]i, [D]v, [D]vi^ |  |  |
|  |  | **Light:Heavy Infections (%)** | - | 0:0 | 100:0 | | 100:0 | 100:0 |  |  |
|  | **Post-** | **Prevalence (n)** | - | 0 (198) | 0 (224) ^[A], [G]iii, iv, v^ | | 11.2 (250) ^[B], [E]iii, iv, v, vi, [G]i^ | 0 (218) ^[A]^ |  |  |
|  |  | **Mean Egg Count (95% CI)** | - | 0 (0-0) | 0 (0-0) [A] | | 0.15 (0.08-0.22) ^[C], [E]iv, [E]v^ | 0 (0-0) ^[A]^ |  |  |
|  |  | **Light:Heavy Infections (%)** | - | 0:0 | 0:0 | | 100:0 | 0:0 |  |  |
| **MDA3** | **Pre-** | **Prevalence (n)** | - | 2.02 (198) ^[C], [D]i, iv, [F]ii, v, vi^ | 1.79 (448) ^[C], [D]i, ii, vi, [F]ii, v^ | | 0 (250) ^[D]i, ii, [F]v^ | 0 (218) ^[B], [D]i, ii, v, [F]vi^ |  |  |
|  |  | **Mean Egg Count (95% CI)** | - | 0.1 (-0.01-0.2) ^[C], [D]i, [F]vi^ | 0.11 (0.01-0.21) ^[A], [D]ii, [F]i, [F]vi^ | | 0 (0-0) | 0 (0-0) ^[C]^ |  |  |
|  |  | **Light:Heavy Infections (%)** | - | 100:0 | 100:0 | | 0:0 | 0:0 |  |  |
|  | **Post-** | **Prevalence (n)** | - | 0 (198) ^[C]^ | 0.89 (224) ^[C], [G]i, ii, iv, v, vi^ | | 0 (250) ^[E]ii, [G]iv, v^ | 0.46 (218) ^[B], [F]i, iv, v, vi^ |  |  |
|  |  | **Mean Egg Count (95% CI)** | - | 0 (0-0) ^[C]^ | 0.002 (0-0.01) ^[A], [E]iv, [E]v^ | | 0 (0-0) | 0.01 (-0.01-0.02) ^[C], [G]v^ |  |  |
|  |  | **Light:Heavy Infections (%)** | - | 0:0 | 100:0 | | 0:0 | 100:0 |  |  |
| **MDA4** | **Pre-** | **Prevalence (n)** | 0 (250) ^[D]i, vi^ | 0 (422) ^[D]i, iii, vi^ | 0 (422) ^[B], [D]i, ii, iii, [F]vi^ | | 0 (250) ^[B], [D]i, ii, [F]v^ | 0 (218) ^[D]i, ii, v, [F]vi^ |  |  |
|  |  | **Mean Egg Count (95% CI)** | 0 (0-0) | 0 (0-0) | 0 (0-0) ^[C]^ | | 0 (0-0) [C] | 0 (0-0) |  |  |
|  |  | **Light:Heavy Infections (%)** | 0:0 | 0:0 | 0:0 | | 0:0 | 0:0 |  |  |
|  | **Post-** | **Prevalence (n)** | 0 (250) | 0 (422) | 0.47 (422) ^[B], [G]i, ii, iii, v, vi^ | | 0.4 (250) ^[B], [E]i, ii, [G]iii, v, vi^ | 0 (218) ^[F]iii, v^ |  |  |
|  |  | **Mean Egg Count (95% CI)** | 0 (0-0) | 0 (0-0) | 0.002 (0-0) ^[C], [E]c, [G]v^ | | 0.02 (-0.02-0.07) ^[C], [E]b, [G]v^ | 0 (0-0) |  |  |
|  |  | **Light:Heavy Infections (%)** | 0:0 | 0:0 | 100:0 | | 100:0 | 0:0 |  |  |
| **MDA5** | **Pre-** | **Prevalence (n)** | - | 0 (198) ^[D]i, vi, [F]iii^ | 0 (224) ^[B], [D]i, ii, [F]iii, vi^ | | 0.4 (250) ^[C], [D]i, ii, [F]iii, iv, v^ | 12.29 (218) ^[A], [D]i, ii, iii, iv, vi^ |  |  |
|  |  | **Mean Egg Count (95% CI)** | - | 0 (0-0) | 0 (0-0) ^[C]^ | | 0.03 (-0.03-0.1) ^[C], [D]i, [D]ii^ | 0.16 (0.1-0.23) ^[A], [D]i, [D]ii, [D]vi^ |  |  |
|  |  | **Light:Heavy Infections (%)** | - | 0:0 | 0:0 | | 100:0 | 100:0 |  |  |
|  | **Post-** | **Prevalence (n)** | - | 0 (198) | 0.89 (224) ^[B], [G]i, ii, iii, iv, vi^ | | 0.4 (250) ^[C], [E]i, ii, [G]iii, iv, vi^ | 0.92 (218) ^[A], [F]i, iii, iv, vi^ |  |  |
|  |  | **Mean Egg Count (95% CI)** | - | 0 (0-0) | 0.003 (0-0.01) ^[C], [E]c, [G]iv^ | | 0.03 (-0.03-0.09) ^[C], [E]ii, [G]iv^ | 0.003 (0-0.01) ^[A], [G]iii^ |  |  |
|  |  | **Light:Heavy Infections (%)** | - | 0:0 | 100:0 | | 100:0 | 100:0 |  |  |
| **MDA6** | **Pre-** | **Prevalence (n)** | 4 (250) ^[A], [D]i, iv^ | 3.55 (422) ^[A], [D]i, ii, iv, v, [F]iii^ | 0.24 (422) ^[A], [D]i, ii, iii, [F]iv, v^ | | 0 (250) ^[D]i, ii, [F]v^ | 0.46 (218) ^[A], [D]i, ii, v [F]iii, iv^ |  |  |
|  |  | **Mean Egg Count (95% CI)** | 0.38 (0-0.77) ^[C], [D]i^ | 0.32 (0.1-0.54) ^[A], [D]i, [F]iii^ | 0.03 (-0.03-0.08) ^[C], [D]i, [D]ii, [F]iii^ | | 0 (0-0) | 0.02 (-0.01-0.05) ^[C], [D]i, [D]ii, [D]v^ |  |  |
|  |  | **Light:Heavy Infections (%)** | 100:0 | 100:0 | 100:0 | | 0:0 | 100:0 |  |  |
|  | **Post-** | **Prevalence (n)** | 0 (250) ^[A]^ | 0 (422) ^[A]^ | 0 (422) ^[A], [G]iii, iv, v^ | | 0 (250) ^[E]ii, [G]iv, v^ | 0 (218) ^[A], [F]iii, v^ |  |  |
|  |  | **Mean Egg Count (95% CI)** | 0 (0-0) ^[C]^ | 0 (0-0) ^[A]^ | 0 (0-0) ^[C]^ | | 0 (0-0) | 0 (0-0) ^[C]^ |  |  |
|  |  | **Light:Heavy Infections (%)** | 0:0 | 0:0 | 0:0 | | 0:0 | 0:0 |  |  |
|  | | | | | | | | |  |  |
|  | | **Province** | **Masvingo** | | | **Mat North** | | **Mat South** |  |  |
|  |  | **District** | **Gutu** | **Mwenezi** | **Binga** | | **Nkayi** | **Insiza** |  |  |
| **MDA1** | **Pre-** | **Prevalence (n)** | 51.04 (199) ^[A]^ | 48.69 (267) ^[A], [D]ii, iii, iv, v, vi^ | 4.72 (253) ^[A]^ | | 6.19 (242) ^[B], [D]iii, iv, v, vi, [F]ii^ | 5.13 (248) ^[A]^ |  |  |
|  |  | **Mean Egg Count (95% CI)** | 23.01 (15.04-30.98) ^[A]^ | 42.87 (32.68-53.07) ^[A], [D]ii, [D]iii^ | 0.47 (0.03-0.91) ^[A]^ | | 1.14 (0.19-2.1) ^[C], [F]ii^ | 0.43 (0.03-0.83) ^[A]^ |  |  |
|  |  | **Light:Heavy Infections (%)** | 72:28 | 42:58 | 100:0 | | 87:13 | 100:0 |  |  |
|  | **Post-** | **Prevalence (n)** | 0 (199) ^[A]^ | 0.38 (267) ^[A], [G]ii, iii, iv, v, vi^ | 0 (253) ^[A]^ | | 7.03 (242) ^[B], [E]ii, iv^ | 0 (248) ^[A]^ |  |  |
|  |  | **Mean Egg Count (95% CI)** | 0 (0-0) ^[A]^ | 0 (0-0) ^[A], [G]iii^ | 0 (0-0) ^[A]^ | | 0.77 (0.33-1.2) ^[C]^ | 0 (0-0) ^[A]^ |  |  |
|  |  | **Light:Heavy Infections (%)** | 0:0 | 100:0 | 0:0 | | 100:0 | 0:0 |  |  |
| **MDA2** | **Pre-** | **Prevalence (n)** | - | 3.49 (267) ^[A], [D]i, iv , v, vi, [F]iii^ | - | | 7.44 (242) ^[A], [D]iii, iv, v, vi, [F]i^ | - |  |  |
|  |  | **Mean Egg Count (95% CI)** | - | 0.09 (0.02-0.16) ^[A], [D]i, [F]iii^ | - | | 2.29 (1.09-3.5) ^[A], [F]i^ | - |  |  |
|  |  | **Light:Heavy Infections (%)** | - | 100:0 | - | | 78:22 | - |  |  |
|  | **Post-** | **Prevalence (n)** | - | 0 (267) ^[A], [G]i, iii^ | - | | 0 (242) ^[A], [E]i^ | - |  |  |
|  |  | **Mean Egg Count (95% CI)** | - | 0 (0-0) | - | | 0 (0-0) ^[A]^ | - |  |  |
|  |  | **Light:Heavy Infections (%)** | - | 0:0 | - | | 0:0 | - |  |  |
| **MDA3** | **Pre-** | **Prevalence (n)** | - | 1.49 (267) ^[A], [D]i, [F]ii, iv, v^ | - | | 0 (240) ^[D]i, ii, [F]iv^ | - |  |  |
|  |  | **Mean Egg Count (95% CI)** | - | 0.03 (-0.01-0.06) ^[C], [D]i, [F]ii^ | - | | 0 (0-0) | - |  |  |
|  |  | **Light:Heavy Infections (%)** | - | 100:0 | - | | 0:0 | - |  |  |
|  | **Post-** | **Prevalence (n)** | - | 0.75 (267) ^[A], [G]i, ii, iv, v, vi^ | - | | 0.83 (242) | - |  |  |
|  |  | **Mean Egg Count (95% CI)** | - | 0.002 (0-0.01) ^[C], [G]i^ | - | | 0.45 (0-56.67) | - |  |  |
|  |  | **Light:Heavy Infections (%)** | - | 100:0 | - | | 0:100 | - |  |  |
| **MDA4** | **Pre-** | **Prevalence (n)** | - | 0 (267) ^[D]i, ii, [F]iii^ | - | | 0 (242) ^[D]i, ii, [F]iii^ | - |  |  |
|  |  | **Mean Egg Count (95% CI)** | - | 0 (0-0) | - | | 0 (0-0) | - |  |  |
|  |  | **Light:Heavy Infections (%)** | - | 0:0 | - | | 0:0 | - |  |  |
|  | **Post-** | **Prevalence (n)** | - | 0 (267) ^[G]i, iii^ | - | | 0 (242) ^[E]i^ | - |  |  |
|  |  | **Mean Egg Count (95% CI)** | - | 0 (0-0) | - | | 0 (0-0) | - |  |  |
|  |  | **Light:Heavy Infections (%)** | - | 0:0 | - | | 0:0 | - |  |  |
| **MDA5** | **Pre-** | **Prevalence (n)** | - | 0 (267) ^[D]i, ii, [F]iii^ | - | | 0 (242) ^[D]i, ii, [F]iii^ | - |  |  |
|  |  | **Mean Egg Count (95% CI)** | - | 0 (0-0) | - | | 0 (0-0) | - |  |  |
|  |  | **Light:Heavy Infections (%)** | - | 0:0 | - | | 0:0 | - |  |  |
|  | **Post-** | **Prevalence (n)** | - | 0 (267) ^[G]i, iii^ | - | | 0 (242) ^[E]i^ | - |  |  |
|  |  | **Mean Egg Count (95% CI)** | - | 0 (0-0) | - | | 0 (0-0) | - |  |  |
|  |  | **Light:Heavy Infections (%)** | - | 0:0 | - | | 0:0 | - |  |  |
| **MDA6** | **Pre-** | **Prevalence (n)** | - | 0 (267) ^[D]i, ii, [F]iii^ | - | | 0 (242) ^[D]i, vi, [F]iii^ | - |  |  |
|  |  | **Mean Egg Count (95% CI)** | - | 0 (0-0) | - | | 0 (0-0) | - |  |  |
|  |  | **Light:Heavy Infections (%)** | - | 0:0 | - | | 0:0 | - |  |  |
|  | **Post-** | **Prevalence (n)** | 0 (199) | 0 (267) ^[G]i, iii^ | 0 (253) | | 0 (242) ^[E]i^ | - |  |  |
|  |  | **Mean Egg Count (95% CI)** | 0 (0-0) | 0 (0-0) | 0 (0-0) | | 0 (0-0) | - |  |  |
|  |  | **Light:Heavy Infections (%)** | 0:0 | 0:0 | 0:0 | | 0:0 | - |  |  |
|  | | | | | | | | |  |  |
|  | | **Province** | **Midlands** | | | | |  |  |  |
|  |  | **District** | **Chirumanzu** | **Gokwe North** | **Mberengwa** | | **Shurugwi** |  |  |  |
| **MDA1** | **Pre-** | **Prevalence (n)** | 30.26 (195) ^[A]^ | 36.68 (199) ^[A]^ | 34.71 (680) ^[A], [D]ii, iii, iv, v, vi^ | | 62.9 (186) ^[A], [D]ii, iii^ |  |  |  |
|  |  | **Mean Egg Count (95% CI)** | 31.5 (18.45-44.55) ^[A]^ | 24.8 (16.86-32.74) ^[A]^ | 25.89 (20.01-31.78) ^[A], [D]ii, [D]iii^ | | 48.33 (35.28-61.38) ^[A]^ |  |  |  |
|  |  | **Light:Heavy Infections (%)** | 71:29 | 56:44 | 64:36 | | 58:42 |  |  |  |
|  | **Post-** | **Prevalence (n)** | 0 (195) ^[A]^ | 0 (199) ^[A]^ | 0 (680) ^[A]^ | | 0 (186) ^[A]^ |  |  |  |
|  |  | **Mean Egg Count (95% CI)** | 0 (0-0) ^[A]^ | 0 (0-0) ^[A]^ | 0 (0-0) ^[A]^ | | 0 (0-0) ^[A]^ |  |  |  |
|  |  | **Light:Heavy Infections (%)** | 0:0 | 0:0 | 0:0 | | 0:0 |  |  |  |
| **MDA2** | **Pre-** | **Prevalence (n)** | - | - | 3.35 (179) ^[A], [D]i, iii, iv, v, vi^ | | 0 (186) ^[D]i^ |  |  |  |
|  |  | **Mean Egg Count (95% CI)** | - | - | 0.12 (0.01-0.23) ^[A], [D]i, [D]iii^ | | 0 (0-0) |  |  |  |
|  |  | **Light:Heavy Infections (%)** | - | - | 100:0 | | 0:0 |  |  |  |
|  | **Post-** | **Prevalence (n)** | - | - | 0 (179) ^[A]^ | | 0 (186) |  |  |  |
|  |  | **Mean Egg Count (95% CI)** | - | - | 0 (0-0) | | 0 (0-0) |  |  |  |
|  |  | **Light:Heavy Infections (%)** | - | - | 0:0 | | 0:0 |  |  |  |
| **MDA3** | **Pre-** | **Prevalence (n)** | - | - | 11.25 (160) ^[A], [D]i, ii, iv, v^ | | 0 (186) ^[D]i^ |  |  |  |
|  |  | **Mean Egg Count (95% CI)** | - | - | 0.44 (0.21-0.66) ^[A], [D]ii, [D]iii^ | | 0 (0-0) |  |  |  |
|  |  | **Light:Heavy Infections (%)** | - | - | 100:0 | | 0:0 |  |  |  |
|  | **Post-** | **Prevalence (n)** | - | - | 0 (179) ^[A]^ | | 0 (186) |  |  |  |
|  |  | **Mean Egg Count (95% CI)** | - | - | 0 (0-0) | | 0 (0-0) |  |  |  |
|  |  | **Light:Heavy Infections (%)** | - | - | 0:0 | | 0:0 |  |  |  |
| **MDA4** | **Pre-** | **Prevalence (n)** | - | - | 0 (179) ^[D]i, ii, iii^ | | 0 (186) |  |  |  |
|  |  | **Mean Egg Count (95% CI)** | - | - | 0 (0-0) | | 0 (0-0) |  |  |  |
|  |  | **Light:Heavy Infections (%)** | - | - | 0:0 | | 0:0 |  |  |  |
|  | **Post-** | **Prevalence (n)** | - | - | 0 (179) | | 0 (186) |  |  |  |
|  |  | **Mean Egg Count (95% CI)** | - | - | 0 (0-0) | | 0 (0-0) |  |  |  |
|  |  | **Light:Heavy Infections (%)** | - | - | 0:0 | | 0:0 |  |  |  |
| **MDA5** | **Pre-** | **Prevalence (n)** | - | - | 0 (179) ^[D]i, ii, iii^ | | 0 (186) |  |  |  |
|  |  | **Mean Egg Count (95% CI)** | - | - | 0 (0-0) | | 0 (0-0) |  |  |  |
|  |  | **Light:Heavy Infections (%)** | - | - | 0:0 | | 0:0 |  |  |  |
|  | **Post-** | **Prevalence (n)** | - | - | 0 (179) | | 0 (186) |  |  |  |
|  |  | **Mean Egg Count (95% CI)** | - | - | 0 (0-0) | | 0 (0-0) |  |  |  |
|  |  | **Light:Heavy Infections (%)** | - | - | 0:0 | | 0:0 |  |  |  |
| **MDA6** | **Pre-** | **Prevalence (n)** | - | - | 0 (179) ^[D]i, ii, iii^ | | 0 (186) |  |  |  |
|  |  | **Mean Egg Count (95% CI)** | - | - | 0 (0-0) | | 0 (0-0) |  |  |  |
|  |  | **Light:Heavy Infections (%)** | - | - | 0:0 | | 0:0 |  |  |  |
|  | **Post-** | **Prevalence (n)** | 0 (195) | 0 (199) | 0 (680) | | 0 (186) |  |  |  |
|  |  | **Mean Egg Count (95% CI)** | 0 (0-0) | 0 (0-0) | 0 (0-0) | | 0 (0-0) |  |  |  |
|  |  | **Light:Heavy Infections (%)** | 0:0 | 0:0 | 0:0 | | 0:0 |  |  |  |

*The mean egg count is calculated per 10mL of urine for the baseline and follow-up surveys. Mean egg counts expressed 95% confidence interval (CI). Descriptions of [X]x are as follows; [X] represents: [A] Significant decrease in paired analysis based on pre- to post-MDA, [B] Significant increase in paired test based on pre- to post-MDA, [C] No significant difference in paired analysis based on pre- to post-MDA, [D] Significant difference in pre to pre in unpaired test between MDAs, [E] Significant difference in post to post in unpaired test between MDAs, [F] No significant difference in pre to pre in unpaired test between MDAs, [G] No significant difference in post to post in unpaired test between MDAs. x) represents: i) compared to MDA1, ii) compared to MDA2, iii) compared against MDA3, iv) compared against MDA4, v) compared against MDA5, vi) compared against MDA6.*
