# Supplementary material for: Identifying hotspots of S. haematobium infection following praziquantel treatment during multiple annual mass drug administration campaigns in Zimbabwe
Source: PLoS Negl Trop Dis. 2025 Sep 24;19(9):e0013546. doi: 10.1371/journal.pntd.0013546 (PMC12520393; doi:10.1371/journal.pntd.0013546)
Supplement: S4 Table — (DOCX) [file pntd.0013546.s006.docx]

|  | | **Province** | **Manicaland** | | | | | |  |  |
| --- | --- | --- | --- | --- | --- | --- | --- | --- | --- | --- |
|  |  | **District** | **Bikita** | **Buhera** | **Chipinge** | | **Makoni** | **Nyanga** |  |  |
| **MDA1** | **Pre-** | **Sample Size** | 102 | 99 | 122 | | 123 | - |  |  |
|  |  | **Cure Rate (%)** | 100 | 100 | 100 | | 100 | - |  |  |
|  |  | **Egg Reduction Rate (%)** | 100 | 100.0 | 100 | | 100 | - |  |  |
|  | **Post-** | **Pre-Mean Egg Count (95% CI)** | 59.54 (44.3-74.78) ^[A]^ | 77.18 (56.17-98.2) ^[A], [D]vi^ | 67.17 (51.39-82.96) ^[A], [D]vi^ | | 55.44 (39.26-71.63) ^[A], [D]vi^ | - |  |  |
|  |  | **Post-Mean Egg Count (95% CI)** | 0 (0-0) ^[A]^ | 0 (0-0) ^[A]^ | 0 (0-0) ^[A]^ | | 0 (0-0) ^[A]^ | - |  |  |
|  |  | **Light:Heavy Infections (%)** | 70:30 | 58:42 | 62:38 | | 73:27 | - |  |  |
| **MDA2** | **Pre-** | **Sample Size** | - | - | - | | - | - |  |  |
|  |  | **Cure Rate (%)** | - | - | - | | - | - |  |  |
|  |  | **Egg Reduction Rate (%)** | - | - | - | | - | - |  |  |
|  | **Post-** | **Pre-Mean Egg Count (95% CI)** | - | - | - | | - | - |  |  |
|  |  | **Post-Mean Egg Count (95% CI)** | - | - | - | | - | - |  |  |
|  |  | **Light:Heavy Infections (%)** | - | - | - | | - | - |  |  |
| **MDA3** | **Pre-** | **Sample Size** | - | - | - | | - | - |  |  |
|  |  | **Cure Rate (%)** | - | - | - | | - | - |  |  |
|  |  | **Egg Reduction Rate (%)** | - | - | - | | - | - |  |  |
|  | **Post-** | **Pre-Mean Egg Count (95% CI)** | - | - | - | | - | - |  |  |
|  |  | **Post-Mean Egg Count (95% CI)** | - | - | - | | - | - |  |  |
|  |  | **Light:Heavy Infections (%)** | - | - | - | | - | - |  |  |
| **MDA4** | **Pre-** | **Sample Size** | - | - | - | | - | - |  |  |
|  |  | **Cure Rate (%)** | - | - | - | | - | - |  |  |
|  |  | **Egg Reduction Rate (%)** | - | - | - | | - | - |  |  |
|  | **Post-** | **Pre-Mean Egg Count (95% CI)** | - | - | - | | - | - |  |  |
|  |  | **Post-Mean Egg Count (95% CI)** | - | - | - | | - | - |  |  |
|  |  | **Light:Heavy Infections (%)** | - | - | - | | - | - |  |  |
| **MDA5** | **Pre-** | **Sample Size** | - | - | - | | - | - |  |  |
|  |  | **Cure Rate (%)** | - | - | - | | - | - |  |  |
|  |  | **Egg Reduction Rate (%)** | - | - | - | | - | - |  |  |
|  | **Post-** | **Pre-Mean Egg Count (95% CI)** | - | - | - | | - | - |  |  |
|  |  | **Post-Mean Egg Count (95% CI)** | - | - | - | | - | - |  |  |
|  |  | **Light:Heavy Infections (%)** | - | - | - | | - | - |  |  |
| **MDA6** | **Pre-** | **Sample Size** | - | 17 | 28 | | 9 | 18 |  |  |
|  |  | **Cure Rate (%)** | - | 100 | 100 | | 100 | 100 |  |  |
|  |  | **Egg Reduction Rate (%)** | - | 100 | 100 | | 100 | 100 |  |  |
|  | **Post-** | **Pre-Mean Egg Count (95% CI)** | - | 16.9 (1.73-32.08) ^[A], [D]i^ | 8.12 (4.03-12.2) ^[A], [D]i^ | | 15.93 (2.95-28.9) ^[A], [D]i^ | 9.52 (4.16-14.87) ^[A]^ |  |  |
|  |  | **Post-Mean Egg Count (95% CI)** | - | 0 (0-0) ^[A]^ | 0 (0-0) ^[A]^ | | 0 (0-0) ^[A]^ | 0 (0-0) ^[A]^ |  |  |
|  |  | **Light:Heavy Infections (%)** | - | 88:12 | 100:0 | | 100:0 | 100:0 |  |  |
|  | | | | | | | | |  |  |
|  | | **Province** | **Mash West** | | | | | **Mashonaland Central** |  |  |
|  |  | **District** | **Chegutu** | **Hurungwe** | **Makonde** | | **Zvimba** | **Guruve** |  |  |
| **MDA1** | **Pre-** | **Sample Size** | 42 | - | 24 | | 114 | - |  |  |
|  |  | **Cure Rate (%)** | 100 | - | 100 | | 100 | - |  |  |
|  |  | **Egg Reduction Rate (%)** | 100 | - | 100 | | 100 | - |  |  |
|  | **Post-** | **Pre-Mean Egg Count (95% CI)** | 50.9 (28.24-73.56) ^[A]^ | - | 10.08 (4.13-16.03) ^[A]^ | | 85.8 (61.71-109.89) ^[A]^ | - |  |  |
|  |  | **Post-Mean Egg Count (95% CI)** | 0 (0-0) ^[A]^ | - | 0 (0-0) ^[A]^ | | 0 (0-0) ^[A]^ | - |  |  |
|  |  | **Light:Heavy Infections (%)** | 71:29 | - | 96:4 | | 60:40 | - |  |  |
| **MDA2** | **Pre-** | **Sample Size** | - | - | - | | - | - |  |  |
|  |  | **Cure Rate (%)** | - | - | - | | - | - |  |  |
|  |  | **Egg Reduction Rate (%)** | - | - | - | | - | - |  |  |
|  | **Post-** | **Pre-Mean Egg Count (95% CI)** | - | - | - | | - | - |  |  |
|  |  | **Post-Mean Egg Count (95% CI)** | - | - | - | | - | - |  |  |
|  |  | **Light:Heavy Infections (%)** | - | - | - | | - | - |  |  |
| **MDA3** | **Pre-** | **Sample Size** | - | - | - | | - | - |  |  |
|  |  | **Cure Rate (%)** | - | - | - | | - | - |  |  |
|  |  | **Egg Reduction Rate (%)** | - | - | - | | - | - |  |  |
|  | **Post-** | **Pre-Mean Egg Count (95% CI)** | - | - | - | | - | - |  |  |
|  |  | **Post-Mean Egg Count (95% CI)** | - | - | - | | - | - |  |  |
|  |  | **Light:Heavy Infections (%)** | - | - | - | | - | - |  |  |
| **MDA4** | **Pre-** | **Sample Size** | - | - | - | | - | - |  |  |
|  |  | **Cure Rate (%)** | - | - | - | | - | - |  |  |
|  |  | **Egg Reduction Rate (%)** | - | - | - | | - | - |  |  |
|  | **Post-** | **Pre-Mean Egg Count (95% CI)** | - | - | - | | - | - |  |  |
|  |  | **Post-Mean Egg Count (95% CI)** | - | - | - | | - | - |  |  |
|  |  | **Light:Heavy Infections (%)** | - | - | - | | - | - |  |  |
| **MDA5** | **Pre-** | **Sample Size** | - | - | - | | - | - |  |  |
|  |  | **Cure Rate (%)** | - | - | - | | - | - |  |  |
|  |  | **Egg Reduction Rate (%)** | - | - | - | | - | - |  |  |
|  | **Post-** | **Pre-Mean Egg Count (95% CI)** | - | - | - | | - | - |  |  |
|  |  | **Post-Mean Egg Count (95% CI)** | - | - | - | | - | - |  |  |
|  |  | **Light:Heavy Infections (%)** | - | - | - | | - | - |  |  |
| **MDA6** | **Pre-** | **Sample Size** | - | - | - | | - | - |  |  |
|  |  | **Cure Rate (%)** | - | - | - | | - | - |  |  |
|  |  | **Egg Reduction Rate (%)** | - | - | - | | - | - |  |  |
|  | **Post-** | **Pre-Mean Egg Count (95% CI)** | - | - | - | | - | - |  |  |
|  |  | **Post-Mean Egg Count (95% CI)** | - | - | - | | - | - |  |  |
|  |  | **Light:Heavy Infections (%)** | - | - | - | | - | - |  |  |
|  | |  |  | | | | | |  |  |
|  | | **Province** | **Mashonaland Central** | | | | | |  |  |
|  |  | **District** | **Mazowe** | **Mt Darwin** | **Muzarabani** | | **Rushinga** | **Shamva** |  |  |
| **MDA1** | **Pre-** | **Sample Size** | - | 126 | 220 | | 110 | 194 |  |  |
|  |  | **Cure Rate (%)** | - | 68.25 | 100 | | 99.09 | 97.42 |  |  |
|  |  | **Egg Reduction Rate (%)** | - | 91.72 | 100 | | 99.99 | 99.63 |  |  |
|  | **Post-** | **Pre-Mean Egg Count (95% CI)** | - | 80.13 (65-95.27) ^[A], [D]iii, [D]iv^ | 120.86 (104.29-137.44) ^[A], [D]iii^ | | 43.89 (30.78-57) ^[A], [D]ii^ | 76.76 (58.21-95.31) ^[A]^ |  |  |
|  |  | **Post-Mean Egg Count (95% CI)** | - | 6.64 (4-9.27) ^[A], [G]iii^ | 0 (0-0) ^[A]^ | | 0.005 (0-0.01) ^[A], [E]ii^ | 0.28 (-0.11-0.68) ^[A]^ |  |  |
|  |  | **Light:Heavy Infections (%)** | - | 56:44 | 34:66 | | 81:19 | 66:34 |  |  |
| **MDA2** | **Pre-** | **Sample Size** | - | - | - | | 23 | - |  |  |
|  |  | **Cure Rate (%)** | - | - | - | | 43.48 | - |  |  |
|  |  | **Egg Reduction Rate (%)** | - | - | - | | 58.91 | - |  |  |
|  | **Post-** | **Pre-Mean Egg Count (95% CI)** | - | - | - | | 3.74 (2.22-5.26) ^[A], [D]iii^ | - |  |  |
|  |  | **Post-Mean Egg Count (95% CI)** | - | - | - | | 1.54 (0.3-2.77) ^[A], [E]ii^ | - |  |  |
|  |  | **Light:Heavy Infections (%)** | - | - | - | | 100:0 | - |  |  |
| **MDA3** | **Pre-** | **Sample Size** | - | 9 | 31 | | 80 | - |  |  |
|  |  | **Cure Rate (%)** | - | 22.22 | 100 | | 100 | - |  |  |
|  |  | **Egg Reduction Rate (%)** | - | 28.57 | 100 | | 100 | - |  |  |
|  | **Post-** | **Pre-Mean Egg Count (95% CI)** | - | 25.93 (7.16-44.69) ^[A], [D]i, [F]iv^ | 0.51 (0.4-0.61) ^[A], [D]i^ | | 9.35 (6.78-11.92) ^[A], [D]i^ | - |  |  |
|  |  | **Post-Mean Egg Count (95% CI)** | - | 18.52 (1.61-35.43) ^[A], [G]i^ | 0 (0-0) ^[A]^ | | 0 (0-0) ^[A]^ | - |  |  |
|  |  | **Light:Heavy Infections (%)** | - | 78:22 | 100:0 | | 99:1 | - |  |  |
| **MDA4** | **Pre-** | **Sample Size** | - | 16 | - | | - | - |  |  |
|  |  | **Cure Rate (%)** | - | 100 | - | | - | - |  |  |
|  |  | **Egg Reduction Rate (%)** | - | 100 | - | | - | - |  |  |
|  | **Post-** | **Pre-Mean Egg Count (95% CI)** | - | 18.13 (8.64-27.61) ^[A], [D]i, [F]iii^ | - | | - | - |  |  |
|  |  | **Post-Mean Egg Count (95% CI)** | - | 0 (0-0) ^[A]^ | - | | - | - |  |  |
|  |  | **Light:Heavy Infections (%)** | - | 88:13 | - | | - | - |  |  |
| **MDA5** | **Pre-** | **Sample Size** | - | - | - | | - | - |  |  |
|  |  | **Cure Rate (%)** | - | - | - | | - | - |  |  |
|  |  | **Egg Reduction Rate (%)** | - | - | - | | - | - |  |  |
|  | **Post-** | **Pre-Mean Egg Count (95% CI)** | - | - | - | | - | - |  |  |
|  |  | **Post-Mean Egg Count (95% CI)** | - | - | - | | - | - |  |  |
|  |  | **Light:Heavy Infections (%)** | - | - | - | | - | - |  |  |
| **MDA6** | **Pre-** | **Sample Size** | - | - | - | | 1 | - |  |  |
|  |  | **Cure Rate (%)** | - | - | - | | 100 | - |  |  |
|  |  | **Egg Reduction Rate (%)** | - | - | - | | 100 | - |  |  |
|  | **Post-** | **Pre-Mean Egg Count (95% CI)** | - | - | - | | 0.33 (-) | - |  |  |
|  |  | **Post-Mean Egg Count (95% CI)** | - | - | - | | 0 (0-0) | - |  |  |
|  |  | **Light:Heavy Infections (%)** | - | - | - | | 100:0 | - |  |  |
|  | | | | | | | | |  |  |
|  | | **Province** | **Mashonaland East** | | | | | **Masvingo** |  |  |
|  |  | **District** | **Chikomba** | **Murehwa** | **Mutoko** | | **UMP** | **Chiredzi** |  |  |
| **MDA1** | **Pre-** | **Sample Size** | 126 | 99 | 129 | | 118 | 118 |  |  |
|  |  | **Cure Rate (%)** | 100 | 100 | 100 | | 100 | 100 |  |  |
|  |  | **Egg Reduction Rate (%)** | 100 | 100 | 100 | | 100 | 100 |  |  |
|  | **Post-** | **Pre-Mean Egg Count (95% CI)** | 55.19 (41.77-68.61) ^[A], [D]vi^ | 74.16 (53.93-94.38) ^[A]^ | 68.69 (52.79-84.59) ^[A]^ | | 102.47 (80.04-124.91) ^[A], [D]ii^ | 91.22 (69.1-113.33) ^[A], [D]ii, [D]v^ |  |  |
|  |  | **Post-Mean Egg Count (95% CI)** | 0 (0-0) ^[A]^ | 0 (0-0) ^[A]^ | 0 (0-0) ^[A]^ | | 0 (0-0) ^[A]^ | 0 (0-0) ^[A]^ |  |  |
|  |  | **Light:Heavy Infections (%)** | 69:31 | 60:40 | 64:36 | | 43:57 | 48:52 |  |  |
| **MDA2** | **Pre-** | **Sample Size** | - | - | 9 | | 9 | 27 |  |  |
|  |  | **Cure Rate (%)** | - | - | 100 | | 66.67 | 100 |  |  |
|  |  | **Egg Reduction Rate (%)** | - | - | 100 | | 82.41 | 100 |  |  |
|  | **Post-** | **Pre-Mean Egg Count (95% CI)** | - | - | 1 (0.66-1.34) ^[A]^ | | 4 (1.45-6.55) ^[A], [D]i^ | 3.46 (2-4.92) ^[A], [D]i, [D]v^ |  |  |
|  |  | **Post-Mean Egg Count (95% CI)** | - | - | 0 (0-0) ^[A]^ | | 0.7 (0.11-1.3) | 0 (0-0) ^[A]^ |  |  |
|  |  | **Light:Heavy Infections (%)** | - | - | 100:0 | | 100:0 | 100:0 |  |  |
| **MDA3** | **Pre-** | **Sample Size** | - | - | - | | - | - |  |  |
|  |  | **Cure Rate (%)** | - | - | - | | - | - |  |  |
|  |  | **Egg Reduction Rate (%)** | - | - | - | | - | - |  |  |
|  | **Post-** | **Pre-Mean Egg Count (95% CI)** | - | - | - | | - | - |  |  |
|  |  | **Post-Mean Egg Count (95% CI)** | - | - | - | | - | - |  |  |
|  |  | **Light:Heavy Infections (%)** | - | - | - | | - | - |  |  |
| **MDA4** | **Pre-** | **Sample Size** | - | - | - | | - | - |  |  |
|  |  | **Cure Rate (%)** | - | - | - | | - | - |  |  |
|  |  | **Egg Reduction Rate (%)** | - | - | - | | - | - |  |  |
|  | **Post-** | **Pre-Mean Egg Count (95% CI)** | - | - | - | | - | - |  |  |
|  |  | **Post-Mean Egg Count (95% CI)** | - | - | - | | - | - |  |  |
|  |  | **Light:Heavy Infections (%)** | - | - | - | | - | - |  |  |
| **MDA5** | **Pre-** | **Sample Size** | - | - | - | | 1 | 27 |  |  |
|  |  | **Cure Rate (%)** | - | - | - | | 0 | 96.29 |  |  |
|  |  | **Egg Reduction Rate (%)** | - | - | - | | 4.17 | 99.06 |  |  |
|  | **Post-** | **Pre-Mean Egg Count (95% CI)** | - | - | - | | 8 (8-8) ^[C]^ | 1.31 (1.03-1.59) ^[A], [D]i, [D]ii^ |  |  |
|  |  | **Post-Mean Egg Count (95% CI)** | - | - | - | | 7.67 (7.67-7.67) ^[C]^ | 0.01 (-0.01-0.04) ^[A]^ |  |  |
|  |  | **Light:Heavy Infections (%)** | - | - | - | | 100:0 | 100:0 |  |  |
| **MDA6** | **Pre-** | **Sample Size** | 10 | - | - | | - | 1 |  |  |
|  |  | **Cure Rate (%)** | 100 | - | - | | - | 100 |  |  |
|  |  | **Egg Reduction Rate (%)** | 100 | - | - | | - | 100 |  |  |
|  | **Post-** | **Pre-Mean Egg Count (95% CI)** | 9.6 (0.33-18.87) ^[A], [D]i^ | - | - | | - | 3.33 (-) |  |  |
|  |  | **Post-Mean Egg Count (95% CI)** | 0 (0-0) ^[A]^ | - | - | | - | 0 (-) |  |  |
|  |  | **Light:Heavy Infections (%)** | 100:0 | - | - | | - | 100:0 |  |  |
|  | | | | | | | | |  |  |
|  | | **Province** | **Masvingo** | | | **Mat North** | | **Mat South** |  |  |
|  |  | **District** | **Gutu** | **Mwenezi** | **Binga** | | **Nkayi** | **Insiza** |  |  |
| **MDA1** | **Pre-** | **Sample Size** | 98 | 130 | 11 | | 5 | - |  |  |
|  |  | **Cure Rate (%)** | 100 | 100 | 100 | | 0 | - |  |  |
|  |  | **Egg Reduction Rate (%)** | 100 | 100 | 100 | | 61 | - |  |  |
|  | **Post-** | **Pre-Mean Egg Count (95% CI)** | 45.08 (30.67-59.48) ^[A]^ | 88.05 (70.06-106.04) ^[A], [D]ii, [D]iii^ | 9.98 (1.37-18.6) ^[A]^ | | 37.27 (-10.08-84.61) ^[C], [F]ii^ | - |  |  |
|  |  | **Post-Mean Egg Count (95% CI)** | 0 (0-0) ^[A]^ | 0 (0-0) ^[A]^ | 0 (0-0) ^[A]^ | | 14.53 (4.16-24.91) ^[C]^ | - |  |  |
|  |  | **Light:Heavy Infections (%)** | 72:28 | 42:58 | 100:0 | | 60:40 | - |  |  |
| **MDA2** | **Pre-** | **Sample Size** | - | 9 | - | | 18 | - |  |  |
|  |  | **Cure Rate (%)** | - | 100 | - | | 100 | - |  |  |
|  |  | **Egg Reduction Rate (%)** | - | 100 | - | | 100 | - |  |  |
|  | **Post-** | **Pre-Mean Egg Count (95% CI)** | - | 2.74 (1.56-3.92) ^[A], [D]i, [F]iii^ | - | | 30.85 (21.53-40.17) ^[A], [F]i^ | - |  |  |
|  |  | **Post-Mean Egg Count (95% CI)** | - | 0 (0-0) ^[A]^ | - | | 0 (0-0) ^[A]^ | - |  |  |
|  |  | **Light:Heavy Infections (%)** | - | 100:0 | - | | 78:22 | - |  |  |
| **MDA3** | **Pre-** | **Sample Size** | - | 4 | - | | - | - |  |  |
|  |  | **Cure Rate (%)** | - | 100 | - | | - | - |  |  |
|  |  | **Egg Reduction Rate (%)** | - | 100 | - | | - | - |  |  |
|  | **Post-** | **Pre-Mean Egg Count (95% CI)** | - | 1.75 (-0.93-4.43) ^[C], [F]ii^ | - | | - | - |  |  |
|  |  | **Post-Mean Egg Count (95% CI)** | - | 0 (0-0) ^[C]^ | - | | - | - |  |  |
|  |  | **Light:Heavy Infections (%)** | - | 100:0 | - | | - | - |  |  |
| **MDA4** | **Pre-** | **Sample Size** | - | - | - | | - | - |  |  |
|  |  | **Cure Rate (%)** | - | - | - | | - | - |  |  |
|  |  | **Egg Reduction Rate (%)** | - | - | - | | - | - |  |  |
|  | **Post-** | **Pre-Mean Egg Count (95% CI)** | - | - | - | | - | - |  |  |
|  |  | **Post-Mean Egg Count (95% CI)** | - | - | - | | - | - |  |  |
|  |  | **Light:Heavy Infections (%)** | - | - | - | | - | - |  |  |
| **MDA5** | **Pre-** | **Sample Size** | - | - | - | | - | - |  |  |
|  |  | **Cure Rate (%)** | - | - | - | | - | - |  |  |
|  |  | **Egg Reduction Rate (%)** | - | - | - | | - | - |  |  |
|  | **Post-** | **Pre-Mean Egg Count (95% CI)** | - | - | - | | - | - |  |  |
|  |  | **Post-Mean Egg Count (95% CI)** | - | - | - | | - | - |  |  |
|  |  | **Light:Heavy Infections (%)** | - | - | - | | - | - |  |  |
| **MDA6** | **Pre-** | **Sample Size** | - | - | - | | - | - |  |  |
|  |  | **Cure Rate (%)** | - | - | - | | - | - |  |  |
|  |  | **Egg Reduction Rate (%)** | - | - | - | | - | - |  |  |
|  | **Post-** | **Pre-Mean Egg Count (95% CI)** | - | - | - | | - | - |  |  |
|  |  | **Post-Mean Egg Count (95% CI)** | - | - | - | | - | - |  |  |
|  |  | **Light:Heavy Infections (%)** | - | - | - | | - | - |  |  |
|  | | | | | | | | |  |  |
|  | | **Province** | **Midlands** | | | | |  |  |  |
|  |  | **District** | **Chirumanzu** | **Gokwe North** | **Mberengwa** | | **Shurugwi** |  |  |  |
| **MDA1** | **Pre-** | **Sample Size** | 59 | 73 | 236 | | 117 |  |  |  |
|  |  | **Cure Rate (%)** | 100 | 100 | 100 | | 100 |  |  |  |
|  |  | **Egg Reduction Rate (%)** | 100 | 100 | 100 | | 100 |  |  |  |
|  | **Post-** | **Pre-Mean Egg Count (95% CI)** | 65.68 (41.01-90.34) ^[A]^ | 65.23 (47.79-82.67) ^[A]^ | 72.19 (57.48-86.9) ^[A], [D]ii, [D]iii^ | | 76.83 (57.82-95.83) ^[A]^ |  |  |  |
|  |  | **Post-Mean Egg Count (95% CI)** | 0 (0-0) ^[A]^ | 0 (0-0) ^[A]^ | 0 (0-0) ^[A]^ | | 0 (0-0) ^[A]^ |  |  |  |
|  |  | **Light:Heavy Infections (%)** | 71:29 | 56:44 | 64:36 | | 58:42 |  |  |  |
| **MDA2** | **Pre-** | **Sample Size** | - | - | 6 | | - |  |  |  |
|  |  | **Cure Rate (%)** | - | - | 100 | | - |  |  |  |
|  |  | **Egg Reduction Rate (%)** | - | - | 100 | | - |  |  |  |
|  | **Post-** | **Pre-Mean Egg Count (95% CI)** | - | - | 3.56 (1.2-5.91) ^[A], [D]i, [F]iii^ | | - |  |  |  |
|  |  | **Post-Mean Egg Count (95% CI)** | - | - | 0 (0-0) ^[A]^ | | - |  |  |  |
|  |  | **Light:Heavy Infections (%)** | - | - | 100:0 | | - |  |  |  |
| **MDA3** | **Pre-** | **Sample Size** | - | - | 18 | | - |  |  |  |
|  |  | **Cure Rate (%)** | - | - | 100 | | - |  |  |  |
|  |  | **Egg Reduction Rate (%)** | - | - | 100 | | - |  |  |  |
|  | **Post-** | **Pre-Mean Egg Count (95% CI)** | - | - | 3.87 (2.65-5.1) ^[A], [D]i, [F]ii^ | | - |  |  |  |
|  |  | **Post-Mean Egg Count (95% CI)** | - | - | 0 (0-0) ^[A]^ | | - |  |  |  |
|  |  | **Light:Heavy Infections (%)** | - | - | 100:0 | | - |  |  |  |
| **MDA4** | **Pre-** | **Sample Size** | - | - | - | | - |  |  |  |
|  |  | **Cure Rate (%)** | - | - | - | | - |  |  |  |
|  |  | **Egg Reduction Rate (%)** | - | - | - | | - |  |  |  |
|  | **Post-** | **Pre-Mean Egg Count (95% CI)** | - | - | - | | - |  |  |  |
|  |  | **Post-Mean Egg Count (95% CI)** | - | - | - | | - |  |  |  |
|  |  | **Light:Heavy Infections (%)** | - | - | - | | - |  |  |  |
| **MDA5** | **Pre-** | **Sample Size** | - | - | - | | - |  |  |  |
|  |  | **Cure Rate (%)** | - | - | - | | - |  |  |  |
|  |  | **Egg Reduction Rate (%)** | - | - | - | | - |  |  |  |
|  | **Post-** | **Pre-Mean Egg Count (95% CI)** | - | - | - | | - |  |  |  |
|  |  | **Post-Mean Egg Count (95% CI)** | - | - | - | | - |  |  |  |
|  |  | **Light:Heavy Infections (%)** | - | - | - | | - |  |  |  |
| **MDA6** | **Pre-** | **Sample Size** | - | - | - | | - |  |  |  |
|  |  | **Cure Rate (%)** | - | - | - | | - |  |  |  |
|  |  | **Egg Reduction Rate (%)** | - | - | - | | - |  |  |  |
|  | **Post-** | **Pre-Mean Egg Count (95% CI)** | - | - | - | | - |  |  |  |
|  |  | **Post-Mean Egg Count (95% CI)** | - | - | - | | - |  |  |  |
|  |  | **Light:Heavy Infections (%)** | - | - | - | | - |  |  |  |

*The mean egg count is calculated per 10mL of urine for the baseline and follow-up surveys. Mean egg counts expressed 95% confidence interval (CI). Descriptions of [X]x are as follows; [X] represents: [A] Significant decrease in paired analysis based on pre- to post-MDA, [B] Significant increase in paired test based on pre- to post-MDA, [C] No significant difference in paired analysis based on pre- to post-MDA, [D] Significant difference in pre to pre in unpaired test between MDAs, [E] Significant difference in post to post in unpaired test between MDAs, [F] No significant difference in pre to pre in unpaired test between MDAs, [G] No significant difference in post to post in unpaired test between MDAs. []x represents: i) compared to MDA1, ii) compared to MDA2, iii) compared against MDA3, iv) compared against MDA4, v) compared against MDA5, vi) compared against MDA6.*
