# Supplementary material for: Identifying hotspots of S. haematobium infection following praziquantel treatment during multiple annual mass drug administration campaigns in Zimbabwe
Source: PLoS Negl Trop Dis. 2025 Sep 24;19(9):e0013546. doi: 10.1371/journal.pntd.0013546 (PMC12520393; doi:10.1371/journal.pntd.0013546)
Supplement: S5 Table — (DOCX) [file pntd.0013546.s007.docx]

|  |  | **Classification** | | | | | | |  |
| --- | --- | --- | --- | --- | --- | --- | --- | --- | --- |
|  | **Time-period to Assess Hotspots** | **No Infection Detected** | **Declined One WHO Category** | **Declined Two WHO Categories** | **Cleared Infection** | **Increased One WHO Category** | **No Data Available** | **PHS** | **Total Number of**  **Districts** |
|  |  |  |  |  |  |  |  |  |  |
| **Approach**  **A** | MDA1 to MDA4 | 4 | 0 | 1 | 17 | 0 | 7 | 0 | 29 |
|  | MDA1 to MDA5 | 0 | 2 | 0 | 10 | 0 | 17 | 0 |  |
|  | MDA1 to MDA6 | 3 | **4** | **3** | 10 | 1 | 7 | **1** |  |
| **Approach**  **B** | MDA1 to MDA4 | 4 | 0 | 0 | 17 | 0 | 7 | **1** |  |
|  | MDA1 to MDA5 | 0 | 1 | 0 | 10 | 0 | 17 | **1** |  |
|  | MDA1 to MDA6 | 3 | **3** | **3** | 10 | 1 | 7 | **2** |  |

*Approach A: Based on difference in WHO prevalence category as described in the methodology, and as described by the Kenya and Tanzania studies [3], Approach B: Based on prevalence ≥ 10% pre-MDA and remained ≥ 5% post-MDA, as described by a study in Zanzibar [4]. WHO Categories are as follows: high, ≥ 50%; moderate, 10–49%; and low < 10% prevalence.*
