# Supplementary material for: Identifying hotspots of S. haematobium infection following praziquantel treatment during multiple annual mass drug administration campaigns in Zimbabwe
Source: PLoS Negl Trop Dis. 2025 Sep 24;19(9):e0013546. doi: 10.1371/journal.pntd.0013546 (PMC12520393; doi:10.1371/journal.pntd.0013546)
Supplement: S1 Text — (DOCX) [file pntd.0013546.s009.docx]

1. Pedersen UB, Midzi N, Mduluza T, Soko W, Stensgaard A-S, Vennervald BJ, et al. Modelling spatial distribution of snails transmitting parasitic worms with importance to human and animal health and analysis of distributional changes in relation to climate. Geospatial Health. 2014;8(2):335-43. doi: 10.4081/gh.2014.23.

2. Pedersen UB, Stendel M, Midzi N, Mduluza T, Soko W, Stensgaard A-S, et al. Modelling climate change impact on the spatial distribution of fresh water snails hosting trematodes in Zimbabwe. Parasites & Vectors. 2014;7(1):536. doi: 10.1186/s13071-014-0536-0.

3. Shen Y, Sung MH, King CH, Binder S, Kittur N, Whalen CC, et al. Modeling Approaches to Predicting Persistent Hotspots in SCORE Studies for Gaining Control of Schistosomiasis Mansoni in Kenya and Tanzania. J Infect Dis. 2020;221(5):796-803. Epub 2019/10/18. doi: 10.1093/infdis/jiz529. PubMed PMID: 31621850; PubMed Central PMCID: PMCPMC7026890.

4. Kittur N, Campbell CH, Binder S, Shen Y, Wiegand RE, Mwanga JR, et al. Discovering, Defining, and Summarizing Persistent Hotspots in SCORE Studies. The American journal of tropical medicine and hygiene. 2020;103(1_Suppl):24-9. doi: 10.4269/ajtmh.19-0815. PubMed PMID: 32400365.
