# Supplementary material for: Identifying hotspots of S. haematobium infection following praziquantel treatment during multiple annual mass drug administration campaigns in Zimbabwe
Source: PLoS Negl Trop Dis. 2025 Sep 24;19(9):e0013546. doi: 10.1371/journal.pntd.0013546 (PMC12520393; doi:10.1371/journal.pntd.0013546)
Supplement: Attachment — Submitted filename: PLOS_review.docx [file pntd.0013546.s011.docx]

**Ref.: Ms. No. PNTD-D-25-00047**

**Identifying hotspots of S. haematobium infection and decreased praziquantel efficacy during multiple mass drug administration in Zimbabwe.**

Abstract

The title is misleading- there is no data in the abstract which shows decreasing efficacy rather than factors associated with PHS versus responding treated areas

Methods: - What are the effects of using the PZQ dose poles versus BWT on the assessing the efficacy? Don’t you see that the PZQ pole was underestimating the quantity/dosage? Hence, low cure rates and ERR?

Results: What is the effects of poor coverage on the prevalence and intensity plus PZQ efficacy? Treatment coverage versus compliance to treatment? What is the effect of herd treatment on the results?

The increase in prevalence or egg intensity following each MDA treatment, does this indicate persistence? Shedding schistosome eggs always continues for several days following treatment. It is probably at 6-weeks evaluation, participants were still shedding eggs. Any comments from authors

Authors examined children for 6-weeks post-treatment, however, the WHO recommends day 14 or day 21 after treatment. The fecundity of the female worm or maturity of the adult worm is always 6-7 weeks. It is probable that authors are reporting re-infections results which give low PZQ efficacy. Any comments from authors?

Result section: - is too long and needs to be shortened

Discussion: -

Paragraphs 1 and 2:- summarize the methods which have already been described in details in the method section- no additional value.

No need to repeat results in the discussion section

Overall- the section is too long- need to be shortened and focused

Conclusion: -0 give the summary of the result not the methods and objectives
