# Supplementary material for: Identifying hotspots of S. haematobium infection following praziquantel treatment during multiple annual mass drug administration campaigns in Zimbabwe
Source: PLoS Negl Trop Dis. 2025 Sep 24;19(9):e0013546. doi: 10.1371/journal.pntd.0013546 (PMC12520393; doi:10.1371/journal.pntd.0013546)
Supplement: Attachment — Submitted filename: revREBUTTAL Hotspots paper 2025 PLOS_reviewer comments.docx [file pntd.0013546.s013.docx]

**Responses to Reviewers’ Comments**

**Ref.: Ms. No. PNTD-D-25-00047
Identifying hotspots of S. haematobium infection and decreased praziquantel efficacy during multiple mass drug administration in Zimbabwe.**

**COMMENT:** The title is misleading- there is no data in the abstract which shows decreasing efficacy rather than factors associated with PHS versus responding treated areas

**RESPONSE**:

We agree that the title may suggest a definitive decline in praziquantel (PZQ) efficacy, which may not be fully supported by the data presented. Our intention was to highlight concerns about potential suboptimal responses observed in previously high-schistosomiasis (PHS) transmission areas compared to historically responding areas. To address this, we propose revising the title to more accurately reflect the focus of our analysis:
**Identifying Hotspots of *Schistosoma haematobium* Infection Following Praziquantel Treatment During Multiple Annual Mass Drug Administration Campaigns in Zimbabwe**

**COMMENT:**

Methods: - What are the effects of using the PZQ dose poles versus BWT on the assessing the efficacy? Don’t you see that the PZQ pole was underestimating the quantity/dosage? Hence, low cure rates and ERR?

**RESPONSE**:

We appreciate the reviewer’s comment regarding the potential influence of dosing methods specifically the use of the WHO praziquantel (PZQ) dose pole versus body weight-based dosing (BWT) on treatment efficacy outcomes, including cure rates and egg reduction rates (ERRs). Indeed, the use of the PZQ dose pole has been shown in some studies to result in under-dosing, particularly in populations with lower body mass indices, such as children in sub-Saharan Africa. Under-dosing can lead to suboptimal therapeutic levels of praziquantel, which in turn may contribute to lower observed cure rates and ERRs. This has been highlighted in several studies indicating that the standard dose pole, although designed for programmatic simplicity, may not always deliver the optimal 40 mg/kg dose in all children, especially in younger or stunted populations. In our study, the praziquantel was administered using the WHO dose pole, in line with the standard national mass drug administration (MDA) protocols. We acknowledge that this method might have introduced variability in the actual dose received by individual children, potentially affecting treatment efficacy outcomes. However, as this was a field-based operational study aligned with national program implementation, our primary objective was to evaluate egg clearance patterns under real-world programmatic conditions rather than to test efficacy under ideal dosing circumstances. We agree that future studies incorporating both dose pole and BWT comparisons with possibly using digital scales in field settings would be valuable in delineating the true impact of dosing discrepancies on efficacy metrics. This would further inform policy decisions regarding the appropriateness of the dose pole across diverse populations, especially where children are investigated.

**COMMENT:**

Results: What is the effects of poor coverage on the prevalence and intensity plus PZQ efficacy? Treatment coverage versus compliance to treatment? What is the effect of herd treatment on the results?

**RESPONSE**:

We thank the reviewer for raising these important points regarding treatment coverage, compliance, and the potential effects of herd treatment on prevalence, intensity, and praziquantel (PZQ) efficacy, especially according to the study design of multiple annual MDA. We have included a detailed explanation over this phenomenon. Poor treatment coverage in MDA programs can significantly compromise the effectiveness of schistosomiasis control efforts. When a substantial portion of the target population does not receive treatment, infected individuals continue to contribute to environmental contamination, sustaining transmission cycles. This undermines the ability to reduce both prevalence and infection intensity over time. In our study, suboptimal coverage may have limited the observed impact of MDA on egg clearance and reinfection rates, particularly in high-transmission settings. While PZQ may demonstrate good individual-level efficacy, the persistent transmission pressure due to untreated individuals can lead to rapid reinfection, thus as in our explanation, masking the drug efficacy at the population level. It is important to distinguish between **treatment coverage** (proportion of eligible individuals offered treatment) and **compliance** (proportion of individuals who actually ingest the medication). High nominal coverage may not equate to effective treatment if compliance is low. In our setting, direct observation of treatment was used where feasible, but it is possible that some individuals may have received tablets but not taken them correctly, leading to suboptimal drug exposure and lower cure rates. Future interventions should consider additional strategies to assess and improve both coverage and compliance, including follow-up verification and behavioral studies to understand reasons for non-compliance.

**The** Herd treatment, or community-wide high-coverage treatment, can generate indirect protection even for untreated individuals by reducing the overall parasite burden in the environment. This “herd effect” may contribute to lower reinfection rates over time if a critical threshold of the population is effectively treated. In our data, the impact of MDA on egg clearance patterns is likely influenced not only by individual drug efficacy but also by the cumulative treatment effect at the community level. In areas where coverage was relatively high, we observed improved clearance and reduced reinfection, consistent with the expected benefits of herd treatment. We agree that further analysis incorporating detailed coverage and compliance data will strengthen interpretations of PZQ efficacy, especially when evaluating community-level impact across multiple MDA rounds. We have left this aspect to another analysis considering the current manuscript is already loaded.

**COMMENT:** The increase in prevalence or egg intensity following each MDA treatment, does this indicate persistence? Shedding schistosome eggs always continues for several days following treatment. It is probably at 6-weeks evaluation, participants were still shedding eggs. Any comments from authors

**RESPONSE**:

We thank the reviewer for raising this important point regarding the timing of post-treatment evaluation and its implications for interpreting increased prevalence or egg intensity following MDA. We agree that residual egg shedding can occur in the days immediately following PZQ treatment, as dying adult worms may continue to expel eggs for a short period. However, **by 6 weeks post-treatment**, residual shedding is generally expected to have resolved. According to existing literature, egg output from treated and damaged worms typically declines significantly within the first 2 - 3 weeks after treatment, making **6 weeks a suitable time point for evaluating both treatment impact and possible reinfection** or persistent infections. In our study, we observed increases in prevalence and/or egg intensity in some settings following each MDA. While we acknowledge the possibility of persistent low-level shedding in a few individuals, the pattern observed across multiple rounds and in larger cohorts suggests that **this trend may reflect a combination of factors**, including:

- **Incomplete parasite clearance (persistence)** possibly due to suboptimal dosing or reduced drug efficacy,
- **Early reinfection** in high-transmission areas with continued exposure to infested water,
- **Variability in diagnostic sensitivity and daily egg output,** which can influence observed prevalence and intensity measures, particularly in low-intensity settings.

We have clarify this point in the revised manuscript and emphasized that **the 6-week time point was selected to reduce the likelihood of residual shedding being mistaken for treatment failure**, and to better reflect real-world post-MDA outcomes under programmatic conditions.

Thank you once again for the constructive comment.

**COMMENT:** Authors examined children for 6-weeks post-treatment, however, the WHO recommends day 14 or day 21 after treatment. The fecundity of the female worm or maturity of the adult worm is always 6-7 weeks. It is probable that authors are reporting re-infections results which give low PZQ efficacy. Any comments from authors?

**RESPONSE**:

This insightful comment regarding the title and interpretation of our findings.

**On timing of post-treatment follow-up:**
We acknowledge the WHO’s recommendation of conducting efficacy assessments at day 14 or 21 post-treatment to minimize the confounding effect of reinfection. Our choice of the 6-week (approximately day 42) follow-up was based on logistical feasibility within ongoing national program operations. We agree this timing increases the possibility of detecting re-infections or late-maturing worms, which may affect egg detection and apparent cure rates.

**On re-infection and worm fecundity:**
The reviewer correctly points out that by 6–7 weeks, juvenile worms could mature and begin egg production, which might confound true efficacy estimation. However, reinfection dynamics vary by exposure and seasonality, and while we cannot rule out re-infection completely, the comparative approach across differing transmission settings aimed to control for this by assuming equal reinfection pressure within the short study window. Nonetheless, we acknowledge this limitation and now clarify this in the discussion section, stating: “The 6-week post-treatment assessment period may reflect not only treatment failure but also possible early re-infection or delayed worm maturation. This should be interpreted with caution and compared with studies following WHO-recommended timepoints (14–21 days).”

**On the implications for PZQ efficacy:**
We appreciate the reviewer’s concern and agree that our data cannot definitively demonstrate reduced drug efficacy. Rather, we observed lower-than-expected egg clearance rates in certain areas, which may indicate suboptimal program impact or highlight areas requiring closer monitoring. We have revised the abstract and discussion to avoid overstating the conclusions and now frame the findings as potentially **“indicative of persistent transmission or suboptimal program impact”** rather than confirmed **“reduced efficacy.”**

We thank the reviewer again for the valuable feedback, which has improved the clarity and scientific rigor of our manuscript.

**COMMENT:** Result section: - is too long and needs to be shortened

**RESPONSE**:

Thank you. We have trimmed the long explanation. We just hope that the outcome is clear enough.

**COMMENT:** Discussion: -

Paragraphs 1 and 2:- summarize the methods which have already been described in details in the method section- no additional value.

No need to repeat results in the discussion section

Overall- the section is too long- need to be shortened and focused

Conclusion: -0 give the summary of the result not the methods and objectives

**RESPONSE**:

Our sincere apologies for getting over elaborative to the point of repeating the results. We have handled the specific sections and overall the whole manuscript to avoid repetitions and over explanation.

**COMMENT:** Please take particular care in English review by an English native speaker, inserting line numbers to facilitate review, and making the text more concise. Even if PLoS NTD does not provide strict wording limits, 10 pages of discussion are exceedingly long.
**RESPONSE:**

This is a valuable comment. We have thoroughly exercise caution on the use of English and we have improved on the presentation and as explained above, we have trimmed the sections that were too elaborative.

**COMMENT:**

5) In the online submission form, you indicated that "The statistical data on the parasitology and treatment (MDA) used to support the findings of this study are available from the corresponding author upon request. Some files are supplied in supplementary data". All PLOS journals now require all data underlying the findings described in their manuscript to be freely available to other researchers, either
- In a public repository
- Within the manuscript itself
- Uploaded as supplementary information.
This policy applies to all data except where public deposition would breach compliance with the protocol approved by your research ethics board. If your data cannot be made publicly available for ethical or legal reasons (e.g., public availability would compromise patient privacy), please explain your reasons by return email and your exemption request will be escalated to the editor for approval. Your exemption request will be handled independently and will not hold up the peer review process, but will need to be resolved should your manuscript be accepted for publication. One of the Editorial team will then be in touch if there are any issues.

**RESPONSE:**
These files have been made available as in the initial submission with clear specification of where the files are located.

**KEY REVIEW CRITERIA REQUIRED FOR ACCEPTANCE?**

**COMMENT:** Reviewer #3: - There is no information what type of study this is, was prospective or retrospective? Also how were participants, districts, villages, schools recruited? Was sample size calculated?
- What was the procedure? Were urine samples taken in the morning? Three days in a row? Was a child considered positive if only all 3 samples were positive? When children treated in relation to sampling and when was post-sampling done after treatment?
-the parasitology methods need to be described (coproscopy and urine filtration)
- It seems from the text that all children were treated, even the children who were negative?
- Is this data from all MDA in the country for these years, or a sub-selection?
- Transmission sites – this seems to be an addition as it isn’t reflected in the objective of identifying hotspots of S. haem–
- It is difficult to understand how time a location were analysed here, as not all locations were had MDA every year, how is this taken into consideration in the analysis?

**RESPONSE**:

We would like to acknowledge the observation by the reviewer of this aspect of the presentation. All information was available but lacked clear presentation in stages. We have taken this opportunity to address this concern especially presenting the sections clearly one after another to make the information clear.

**RESULTS:**

**COMMENT:** Reviewer #3: - There is methods in this section, should be only be results (e.g. the first paragraph of the results section)
- Figures – the circles are too small to see the information presented in them
- Use of the word sentinel sites, what is this?
- I am confused about use of village and district? What about schools? - should be described in methods
- There are a lot of results here not described in the objectives or methods, but the main results, prevalence and efficacy are in the supplemental tables.

**RESPONSE**:

- We have removed the inclusion of methods description in the results section which really streamlined the content and made the section shorter.
- We have made markings in the figures to be visible.
- The words and explanation of sentinel sites and clear description of the village, districts and the school has been provided at the appropriate section.
- We have aligned the results to the objectives and methods used. The supplementary tables may contain some additional results not possible to present in the main results section of the manuscript.

**CONCLUSIONS:**

**COMMENT:** Reviewer #1: - The conclusion needs revision to reflect the results and not the methods used
- Authors need to add in limitations- 6-weeks post-MDA evaluation need to be acknowledged
- Yes, the discussion is clear on the public health importance of the results
- The public health relevance of the topic is clear- the hotspot issues and the control of schistosomiasis

**RESPONSE**:

- We are grateful to the reviewers for bringing this issue on the presentation of the conclusion. We have corrected the conclusion to give precisely the major outcome, that has assisted in removing excess information.
- The limitation of 6-weeks post MDA is again highlighted especially with reference to the areas where transmission is heavy making it possible for immature worms not susceptible to PZQ treatment becoming mature by the evaluation time after 6 weeks and contributing to likely unclear attributes to persistent infection but not PZQ inefficacy.
- Thank you for the comments and the contribution of the work on the persistent transmission and need to strategize control efforts.

**COMMENT:** Reviewer #3: I think the main conclusion is that regular MDA was effective in reducing prevalence, with few hotspots and the drug was effective, yet this doesn't seem to be written anywhere. I would add a conclusion as it relates to the objectives.

**RESPONSE**:

Thank you for this comment. We did not realize that it was not coming out clearly. We have amended and including this is the conclusion.

**COMMENT:** Reviewer #3: S2A – no infection detected and no data are almost the same colour
- significant digits – decimal points should be consistent in table
- tables aren’t correctly numbered S2C then S4
- there is an overwhelming amount of data in the supplementary tables, maybe there is another way to present this data more concisely?

**RESPONSE**:

Thank you for the observation.

- The supplementary plots were addressed.
- We would like to acknowledge that the study utilizing MDA data from 5 consecutive years collected at multiple sentinel sites is massive and only possible for additional presentation as supplementary tables. We have tried to make it accessible in a clearer systematic way.

**SUMMARY AND GENERAL COMMENTS:**

**COMMENT:** Reviewer #1: Overall, the work is of high quality and the analysis is very clear though too long to read this work, it needs readjustment. The topic is of relevance as the schistosomiasis community currently think of how to tackle hotspot areas which maintain transmission.

**RESPONSE**:

We take this opportunity to thank the review for acknowledging the quality of the manuscript and being open at pointing out at the length of the manuscript. We have addressed the concerns and maintained the high quality of the data presented.

**COMMENT:** Reviewer #2: Overall comments:
However, there are few minor corrections which need to be made before publication as follows:
Study design and inclusion criteria:
I was wandering whether MDA1, MD2, MD3 etc refers to treatment arm or the number of annual MDA rounds implemented during the life of the mass drug administration (MDA) programme in Zimbabwe. For example MDA1 refers to annual MDA for year 1, MDA2 refers to annual MDA for year 2, MDA3 refers to annual MDA for year 3 etc. Please revisit this.
**RESPONSE**:

We have taken this comment to redress the issue of clarity on the time points of the assessment pre- and post-MDA. The MDA were conducted annually thereby referred to as MDA1 as of the first year, then MDA2 as of the following second year.

**COMMENT:** Reviewer #3: Thank you for the opportunity to review this very important and interesting manuscript on the persistent schistosomiasis hotspots in Zimbabwe. This data is very useful in for better understanding hotspots dynamics over time.
**RESPONSE**:

We are really honored by this comment. Thank you.

**COMMENT:** Reviewer #3: In general:
- The manuscript needs an English review as some areas are quite unclear
o The use of many uncommon abbreviations creates confusion for the reader
- Continuous line numbers needs to be inserted to the manuscript
- It maybe useful to have more subheadings that follow the objectives in each section: e.g. Methods: Sample and setting, Sample collection and parasitology, Data collection, Prevalence and infection intensity, treatment efficacy, predictors of hotspots; Results: prevalence and infection intensity, treatment efficacy, predictors of hotspots etc. For more information the authors may want to use the following checklist <https://www.equator-network.org/>
- The manuscript should be reviewed for clarity and conciseness
o There is a quite a bit of repeating text and methods in the results and discussion (e.g. inclusion criteria in the methods and then again in the figure caption)
o The manuscript is quite long
o It is difficult to follow the objectives, through to the methods, results and discussion

**RESPONSE**:

We appreciate this constructive comment. The submitted version of the manuscript has been thoroughly revised taking into consideration the concerns expressed by the reviewer:

- Simple and clear use of English.
- Defined the abbreviations and terms used.
- Manuscript version and indicating the numbering.
- Specific objectives outlined separately to make the work clear.
- Repetitive sections have been removed.
- As explained above methods removed from the results sections and not repeating results in the discussion.
- Trimmed or shortened the manuscript to reasonable level.
- Clarified the specific sections of the manuscript.

**RESPONSE TO COMMENTS ON MAPS:**

Both maps were generated using QGIS, Version [3.22.2]. Appropriate format of the maps have been used.
