# Supplementary material for: Identifying hotspots of S. haematobium infection following praziquantel treatment during multiple annual mass drug administration campaigns in Zimbabwe
Source: PLoS Negl Trop Dis. 2025 Sep 24;19(9):e0013546. doi: 10.1371/journal.pntd.0013546 (PMC12520393; doi:10.1371/journal.pntd.0013546)
Supplement: Attachment — Submitted filename: REBUTTAL Hotspots paper Aug2025 .docx [file pntd.0013546.s014.docx]

**Responses to Reviewers’ Comments**

**Ref.: Ms. No. PNTD-D-25-00047
Identifying hotspots of S. haematobium infection and decreased praziquantel efficacy during multiple mass drug administration in Zimbabwe.**

We would like to convey our utmost gratitude to the comments raised by the reviewers that has contributed to reshaping and making the manuscript concise. The comments from the reviewers have been very constructive while it has been difficulty to cut out some of the sections considered a bit longer. We exercised extreme care making the other sections very short while the results section ended up being a bit longer. We trimmed it further but the section is very important as we had to bring out the supporting evidence of the defined persistent hotspots. It is from the observation over the 6 rounds of MDAs and the infection characteristics that brings out the essence of the manuscript. However, the section has been trimmed further, and simplified to make it easier to the reader to follow.

Below are point-by-point responses to the comments as raised by the reviewers:

**Reviewer’s comments: Reviewer #3: Abstract**
-the abstract is quite long

**Responses:**

The abstract had been trimmed further to less than 300 words.

**Reviewer’s comments:**
-decreasing efficacy – the further they are from water the more likely they are to have decreasing efficacy? This seems odd...

**Responses:**

This referred to low infection. The statement has been made clear.

**Reviewer’s comments:**

Intro
Line 91-107 – I would recommend removing this paragraph. More or less, it states what is said in the previous paragraph, I think it is enough to just say that there are documented cases of persistent hotspots is Zanzibar and Kenya and that some studies have linked this issue to PZQ effectiveness (Senegal).

**Responses:**

We decided to keep this section as it highlight substantively the existence of other similar attempts in these named countries.

**Reviewer’s comments:**
-Line 112-114 – what are the definitions of PPHS and EPHS exactly? Persistent hotspot of schistosomiasis prevalence simply means a hotspot, or?
**Responses:**

The terms were explained clearly and after checking we find that the presentation suffices and is clear.

**METHODS
Reviewer’s comments:**
Iine 135-136 – inclusion criteria for assessments? Or inclusion criteria for participants to participate were A) to have provided 3 urine samples both pre and post MDA? And B, I’m not sure what this means. Only treated children were included in the study? Or is it inclusion criteria for each arm of the study?

**Responses:**

The description of the populations considered in the different sections of the analysis have been made clearer.

**Reviewer’s comments:**
Line 168-171- this is unclear, how are persistent hotspots with prevalences of schistosomiasis determined by change in WHO prevalence risk category as meaningful decline in prevalence risk category? I would strongly suggest rephrasing.

**Responses:**

The definition of hotspots is taken into consideration following the WHO classification. However, The section has been revised.

**Reviewer’s comments:**
Line 176 – best to say that this is a second approach for PPHS

**Responses:**

Comment considered and approach clarified as in the preceding sections for the PPHS and EPHS.

**Reviewer’s comments:**
Line 191- this aspect is still not reflected in the objectives in the introduction (ID transmission sites)

**Responses:**

The objectives seem to be clear and considered in all the relevant sections.

**RESULTS**

**Reviewer’s comments:**
Section 1 prevalence and egg count
- There is so much information and no visual representation, as a reader it is very difficult to follow all of this information and it is a lot of text (this section is almost 4 pages). Two graphs would be very helpful for the reader and could eliminate a lot of the text as the results are 9.5 pages. One graph with prevalence by district on y axis and time on x, plotting pre and post each MDA, the other graph the same but with Egg count. Also, the y axis could indicate the WHO categories of prevalence.

**Responses:**

This has been handled by summarizing with reference to the graphs presented. A lot of effort was made to limit the number of graphs and the considered graphs account for the key data that contributes to the definition and explanation of the PPHS and EPHS.

**Reviewer’s comments:**
- There isn’t a description of what was compared, in the first section, the results go through preMDA1 to post MDA1. Then in the MDA2 section, there is comparison of both pre and post MDA1 and Pre MDA 2 , but this isn’t mentioned in the methods.

**Responses:**

In an attempt to compare the variations in the occurrence of the infection post MDA to pre MDA this was additionally compared to the other time points. However, this has been revised to stick to simple comparisons leaving the reader to deduce from further reading instead of writing everything down.

**Reviewer’s comments:**
-please revise the sub-headings to something like Change in prevalence and egg count pre and post each MDA, just MDA1 is not very helpful

**Responses:**

Subheadings revised and elaborated

**Reviewer’s comments:**

- Units should be present right after the number, not the CI e.g. 1.14 eggs/10ml (CI…)

**Responses:**

We are thankful to the reviewers for this observation. Even after revising the section several times we never picked this mistake. Thank you. Corrected throughout the results section.

**Reviewer’s comments:**

- Timelines are often missing in the first paragraph and in the methods- changing the sub-headings could help
Line 257-263 - this should be in the methods

**Responses:**

This part of the description is left in the results section to make it clear for the following statement providing results and referring to the S3 Table.

**Reviewer’s comments:**

Line 270-271 –Where are the numbers, in a table perhaps? If so should refer to table here.

**Responses:**

Reference made to the Tables.

**Reviewer’s comments:**

Line 273-280 – this isn’t clear, the paper discusses significant decrease in prevalence and then speaks of infection intensity in this and another district as an exception to having a statistically significant reduction in prevalence? Also, Line 273-274 says a change of prevalence from 66% to 24% wasn’t significant? Why is that, was the sample size small? Seems like a large difference.

**Responses:**

This has been clarified and referred to the time point that was at post-MDA1

**Reviewer’s comments:**

Lastly, decrease infection from pre-MDA1 until when?

**Responses:**

up to post-MDA1

**Reviewer’s comments:**

Lines 351-354 – the reader needs both numbers here, both the pre-MDA1 and preMDA6 prevalences and egg counts

**Responses:**

The information is provided and reads well now.

**Reviewer’s comments:**
Lines 363-366 - this is methods should be in the methods, also the second sentence isn’t clear, inclusion criteria of the calculations?

**Responses:**

Comments addressed by removing the description of the methods and process in the results section.

**Reviewer’s comments:**

Line367-368 – not sure what this means, can probably delete this text altogether, also seems to be methods, does it mean that the same children were not followed across all MDA?
Lines 367-369 - can be deleted

**Responses:**

Statements clarified and redundant information removed. Same children were follow-up.

**Reviewer’s comments:**

Lines 413-427 are methods, please move there, minus the reference to the tables

**Responses:**

Thank you. Comment considered referring to the table.

**Reviewer’s comments:**

Line 380 – Mean egg counts expressed 95% CI – this text is out of place, please delete

**Responses:**

Removed

**Reviewer’s comments:**

Line 381-382 – this sentence needs to be rephrased, paired t test don’t change, it would be no significant difference between ….
Line 386-397 – should be in the methods

**Responses:**

Recommendation considered

**Reviewer’s comments:**

Line 386-387 – suggest changing to “post-MDA infection status for every village…"
Line 390 – not clear, should be rephrased

**Responses:**

Comment addressed

**Reviewer’s comments:**

Line 404-405 – have the authors considered removing some districts from the analysis for some time points? For example, I wonder if MDAs with a sample size of 1 or 5 individuals should be included in analysis?

**Responses:**

The data was collected from selected sentinel sites including timed periods at a larger number of sites. This sampling approach contributed to the vibrancy of the manuscript to depict the hotspots. However, the reference to MDAs with 1 or 5 infected individuals has been left out since it is difficult to include in the analysis.

**Reviewer’s comments:**
Line 413-427 and lines 454-463 and line 486 and lines 512-519 and lines 527-537 and 541-545– this is all methods and should be moved from the results to the methods

**Responses:**

Thank you for the comments. Appropriate information relevant to methods was moved to the section while repetitive information was deleted.

**DISCUSSION
Reviewer’s comments:**

Lines 618-621 – this is unclear, I suggest rephrasing

**Responses:**

Comment considered and statement rephrased

**Reviewer’s comments:**

Lines 640-642 – this is a very bold statement, I don’t think the results support this claim
- In regards to the 6 weeks and the limitations, PZQ only kills adults worms, so juveniles in the host during treatment would not be affected and could be releasing eggs at 6 weeks, when the post-treatment sampling was done.

**Responses:**

Since the data is not complete, the statement was revised to reflect uncertainty of the situation and the diagnosis conducted. The final wording was revised.

**FIGURES
Reviewer’s comments:**

Figure 1 is not mentioned in the text, also the caption has a description of methods (inclusion criteria) , which shouldn’t be there.
Fig 1 should be revised and redone, it is confusing and doesn’t present the data clearly
-doesn’t have appropriate column headings (MDA isn’t a study design, also n= is not prevalence, how many children is n, all together or in each region?)
-what are the brackets from pre and post for? What do they mean?

**Responses:**

The Figure 1 is mentioned in the text. However, we have revised and made it clear.

**Reviewer’s comments:**

Figure 2
- The dots on the map, have not been addressed. It is better to indicate the site with a symbol, for example a circle and then next to that add another circle with the infection intensities that are bigger so that the reader can see them

**Responses:**

The Figure 2 is a map that is showing shading. We remain clear that shading and the presentation suffices.

**TABLES
Reviewer’s comments:**

Tables should be ordered in the order they appear in the text. The first table mentioned is Table 1 followed by Supptable 4. Therefore Supp table 4 should be supp table 1.
Table S4 – please show the unit of prevalence in table i.e. Prevalence in % (n)
-again, as before significant digits, all data has to be presented to the same decimal place (i.e. if you report one decimal point, 45.6, all numbers for this measure in the table should be to one decimal place)

**Responses:**

The presentation and reference of the Tables has been addressed.
