# Supplementary material for: Identifying hotspots of S. haematobium infection following praziquantel treatment during multiple annual mass drug administration campaigns in Zimbabwe
Source: PLoS Negl Trop Dis. 2025 Sep 24;19(9):e0013546. doi: 10.1371/journal.pntd.0013546 (PMC12520393; doi:10.1371/journal.pntd.0013546)
Supplement: Peer review history

# Peer review: Decision Letter 0

**DOI:** 10.1371/journal.pntd.0013546.r001

**Authors:** Francesca Tamarozzi

18 Mar 2025

PNTD-D-25-00047

Identifying hotspots of S. haematobium infection and decreased praziquantel efficacy during multiple mass drug administration in Zimbabwe.

PLOS Neglected Tropical Diseases

Dear Dr. Mduluza,

Thank you for submitting your manuscript to PLOS Neglected Tropical Diseases. After careful consideration, we feel that it has merit but does not fully meet PLOS Neglected Tropical Diseases's publication criteria as it currently stands. Therefore, we invite you to submit a revised version of the manuscript that addresses the points raised during the review process. Please take particular care in English review by an English native speaker, inserting line numbers to facilitate review, and making the text more concise. Even if PLoS NTD does not provide strict wording limits, 10 pages of discussion are exceedingly long.

Please submit your revised manuscript within 60 days May 17 2025 11:59PM. If you will need more time than this to complete your revisions, please reply to this message or contact the journal office at plosntds@plos.org. When you're ready to submit your revision, log on to https://www.editorialmanager.com/pntd/ and select the 'Submissions Needing Revision' folder to locate your manuscript file.

Please include the following items when submitting your revised manuscript:

* A rebuttal letter that responds to each point raised by the editor and reviewer(s). You should upload this letter as a separate file labeled 'Response to Reviewers'. This file does not need to include responses to any formatting updates and technical items listed in the 'Journal Requirements' section below.

* A marked-up copy of your manuscript that highlights changes made to the original version. You should upload this as a separate file labeled 'Revised Manuscript with Track Changes'.

* An unmarked version of your revised paper without tracked changes. You should upload this as a separate file labeled 'Manuscript'.

If you would like to make changes to your financial disclosure, competing interests statement, or data availability statement, please make these updates within the submission form at the time of resubmission. Guidelines for resubmitting your figure files are available below the reviewer comments at the end of this letter.

We look forward to receiving your revised manuscript.

Kind regards,

Francesca Tamarozzi

Section Editor

PLOS Neglected Tropical Diseases

Francesca Tamarozzi

Section Editor

PLOS Neglected Tropical Diseases

Shaden Kamhawi

co-Editor-in-Chief

PLOS Neglected Tropical Diseases

orcid.org/0000-0003-4304-636XX

Paul Brindley

co-Editor-in-Chief

PLOS Neglected Tropical Diseases

orcid.org/0000-0003-1765-0002

**Journal Requirements:**

1) Please ensure that the CRediT author contributions listed for every co-author are completed accurately and in full.

At this stage, the following Authors/Authors require contributions: Takafira Mduluza. Please ensure that the full contributions of each author are acknowledged in the "Add/Edit/Remove Authors" section of our submission form.

The list of CRediT author contributions may be found here: https://journals.plos.org/plosntds/s/authorship#loc-author-contributions

2) We ask that a manuscript source file is provided at Revision. Please upload your manuscript file as a .doc, .docx, .rtf or .tex. If you are providing a .tex file, please upload it under the item type u2018LaTeX Source Fileu2019 and leave your .pdf version as the item type u2018Manuscriptu2019.

3) We have noticed that you have uploaded Supporting Information files, but you have not included a list of legends. Please add a full list of legends for your Supporting Information files after the references list.

4) Some material included in your submission may be copyrighted. According to PLOSu2019s copyright policy, authors who use figures or other material (e.g., graphics, clipart, maps) from another author or copyright holder must demonstrate or obtain permission to publish this material under the Creative Commons Attribution 4.0 International (CC BY 4.0) License used by PLOS journals. Please closely review the details of PLOSu2019s copyright requirements here: PLOS Licenses and Copyright. If you need to request permissions from a copyright holder, you may use PLOS's Copyright Content Permission form.

Please respond directly to this email and provide any known details concerning your material's license terms and permissions required for reuse, even if you have not yet obtained copyright permissions or are unsure of your material's copyright compatibility. Once you have responded and addressed all other outstanding technical requirements, you may resubmit your manuscript within Editorial Manager. 

Potential Copyright Issues:

- Figures 2, 3, S1A, S1B, S2A, and S2B. Please provide a direct link to the base layer of the map (i.e., the country or region border shape) and ensure this is also included in the figure legend; and (b) provide a link to the terms of use / license information for the base layer image or shapefile. We cannot publish proprietary or copyrighted maps (e.g. Google Maps, Mapquest) and the terms of use for your map base layer must be compatible with our CC BY 4.0 license.

Note: if you created the map in a software program like R or ArcGIS, please locate and indicate the source of the basemap shapefile onto which data has been plotted.

If your map was obtained from a copyrighted source please amend the figure so that the base map used is from an openly available source. Alternatively, please provide explicit written permission from the copyright holder granting you the right to publish the material under our CC BY 4.0 license.

If you are unsure whether you can use a map or not, please do reach out and we will be able to help you. The following websites are good examples of where you can source open access or public domain maps:

* U.S. Geological Survey (USGS) - All maps are in the public domain. (http://www.usgs.gov)

* PlaniGlobe - All maps are published under a Creative Commons license so please cite u201cPlaniGlobe, http://www.planiglobe.com, CC BY 2.0u201d in the image credit after the caption. (http://www.planiglobe.com/?lang=enl)

* Natural Earth - All maps are public domain. (http://www.naturalearthdata.com/about/terms-of-use/).

5) In the online submission form, you indicated that "The statistical data on the parasitology and treatment (MDA) used to support the findings of this study are available from the corresponding author upon request. Some files are supplied in supplementary data". All PLOS journals now require all data underlying the findings described in their manuscript to be freely available to other researchers, either

- In a public repository

- Within the manuscript itself

- Uploaded as supplementary information.

This policy applies to all data except where public deposition would breach compliance with the protocol approved by your research ethics board. If your data cannot be made publicly available for ethical or legal reasons (e.g., public availability would compromise patient privacy), please explain your reasons by return email and your exemption request will be escalated to the editor for approval. Your exemption request will be handled independently and will not hold up the peer review process, but will need to be resolved should your manuscript be accepted for publication. One of the Editorial team will then be in touch if there are any issues.

6) Please ensure that the funders and grant numbers match between the Financial Disclosure field and the Funding Information tab in your submission form. Note that the funders must be provided in the same order in both places as well.

- State the initials, alongside each funding source, of each author to receive each grant. For example: "This work was supported by the National Institutes of Health (####### to AM; ###### to CJ) and the National Science Foundation (###### to AM)."

- State what role the funders took in the study. If the funders had no role in your study, please state: "The funders had no role in study design, data collection and analysis, decision to publish, or preparation of the manuscript.".

If you did not receive any funding for this study, please simply state: u201cThe authors received no specific funding for this work.u201d

**Reviewers' Comments:**

Reviewer's Responses to Questions

**Key Review Criteria Required for Acceptance?**

As you describe the new analyses required for acceptance, please consider the following:

**Methods:**

-Are the objectives of the study clearly articulated with a clear testable hypothesis stated?

-Is the study design appropriate to address the stated objectives?

-Is the population clearly described and appropriate for the hypothesis being tested?

-Is the sample size sufficient to ensure adequate power to address the hypothesis being tested?

-Were correct statistical analysis used to support conclusions?

-Are there concerns about ethical or regulatory requirements being met?

Reviewer #1: - The objectives are very clear and with a clear hypothesis

- The study design is appropriate and covers the objective

- The population is School children, appropriate population and was involved in intervention studies

- The sample size calculation is clear

- Statistical analysis is clear

- No ethical issues, the study assess secondary data

Reviewer #2: -Are the objectives of the study clearly articulated with a clear testable hypothesis stated? Yes

-Is the study design appropriate to address the stated objectives? Yes

-Is the population clearly described and appropriate for the hypothesis being tested? Yes

-Is the sample size sufficient to ensure adequate power to address the hypothesis being tested? Yes

-Were correct statistical analysis used to support conclusions? Yes

-Are there concerns about ethical or regulatory requirements being met? No

Reviewer #3: - There is no information what type of study this is, was prospective or retrospective? Also how were participants, districts, villages, schools recruited? Was sample size calculated?

- What was the procedure? Were urine samples taken in the morning? Three days in a row? Was a child considered positive if only all 3 samples were positive? When children treated in relation to sampling and when was post-sampling done after treatment?

-the parasitology methods need to be described (coproscopy and urine filtration)

- It seems from the text that all children were treated, even the children who were negative?

- Is this data from all MDA in the country for these years, or a sub-selection?

- Transmission sites – this seems to be an addition as it isn’t reflected in the objective of identifying hotspots of S. haem–

- It is difficult to understand how time a location were analysed here, as not all locations were had MDA every year, how is this taken into consideration in the analysis?

**********

**Results:**

-Does the analysis presented match the analysis plan?

-Are the results clearly and completely presented?

-Are the figures (Tables, Images) of sufficient quality for clarity?

Reviewer #1: - Yes, the analysis present matches the analysis plan

- The results are clear except that the section is too long

- Yes, tables are of high quality

Reviewer #2: -Does the analysis presented match the analysis plan? Yes

-Are the results clearly and completely presented? Yes

-Are the figures (Tables, Images) of sufficient quality for clarity? Yes

Reviewer #3: - There is methods in this section, should be only be results (e.g. the first paragraph of the results section)

- Figures – the circles are too small to see the information presented in them

- Use of the word sentinel sites, what is this?

- I am confused about use of village and district? What about schools? - should be described in methods

- There are a lot of results here not described in the objectives or methods, but the main results, prevalence and efficacy are in the supplemental tables

**********

**Conclusions:**

-Are the conclusions supported by the data presented?

-Are the limitations of analysis clearly described?

-Do the authors discuss how these data can be helpful to advance our understanding of the topic under study?

-Is public health relevance addressed?

Reviewer #1: - The conclusion needs revision to reflect the results and not the methods used

- Authors need to add in limitations- 6-weeks post-MDA evaluation need to be acknowledged

- Yes, the discussion is clear on the public health importance of the results

- The public health relevance of the topic is clear- the hotspot issues and the control of schistosomiasis

Reviewer #2: -Are the conclusions supported by the data presented? Yes

-Are the limitations of analysis clearly described? Yes

-Do the authors discuss how these data can be helpful to advance our understanding of the topic under study? Yes

-Is public health relevance addressed? Yes

Reviewer #3: I think the main conclusion is that regular MDA was effective in reducing prevalence, with few hotspots and the drug was effective, yet this doesn't seem to be written anywhere. I would add a conclusion as it relates to the objectives.

**********

**Editorial and Data Presentation Modifications?**

Use this section for editorial suggestions as well as relatively minor modifications of existing data that would enhance clarity. If the only modifications needed are minor and/or editorial, you may wish to recommend “Minor Revision” or “Accept”.

Reviewer #1: None

Reviewer #2: Yes, see Summary

Reviewer #3: S2A – no infection detected and no data are almost the same color

- significant digits – decimal points should be consistent in table

- tables aren’t correctly numbered S2C then S4

- there is an overwhelming amount of data in the supplementary tables, maybe there is another way to present this data more concisely?

**********

**Summary and General Comments:**

Use this section to provide overall comments, discuss strengths/weaknesses of the study, novelty, significance, general execution and scholarship. You may also include additional comments for the author, including concerns about dual publication, research ethics, or publication ethics. If requesting major revision, please articulate the new experiments that are needed.

Reviewer #1: Overall, the work is of high quality and the analysis is very clear though too long to read this work, it needs readjustment. The topic is of relevance as the schistosomiasis community currently think of how to tackle hotspot areas which maintain transmission

Reviewer #2: Overall comments:

The research article titled “Identifying hotspots of S. haematobium infection and decreased praziquantel efficacy during multiple mass drug administration in Zimbabwe” by Takafira Mduluza et al 2025 addresses an important and very interesting research topic relating to the control and elimination of schistosomiasis in school children in Zimbabwe and Africa. This article examines in great depth and details the impact of repeated rounds of mass drug administration using praziquantel in school children in Zimbabwe and describes the development of Schistosoma haematobium transmission hotspots as it has been observed in many other African countries. The study has a sound design and methodology and the findings are credible. The authors concludes by recommending additional interventions to complement preventive chemotherapy such as water, sanitation and hygiene (WASH) and snail control as a strategy for effective and sustainable control and elimination of schistosomiasis in hot spot settings. There is no doubt about the public health relevance of the findings presented and hence I strongly recommend the article for publication and wider dissemination. However, there are few minor corrections which need to be made before publication as follows:

Study design and inclusion criteria:

I was wandering whether MDA1, MD2, MD3 etc refers to treatment arm or the number of annual MDA rounds implemented during the life of the mass drug administration (MDA) programme in Zimbabwe. For example MDA1 refers to annual MDA for year 1, MDA2 refers to annual MDA for year 2, MDA3 refers to annual MDA for year 3 etc. Please revisit this.

Results:

I was impressed by the way the figures (Figure 1 to figure 9) are presented in the manuscript text because this facilitates logical flow and easy comprehension by the reader. However, the same figures re-appear again in a separate section after the references and again as supporting information. I am not sure whether this is a journal requirement or an error but it can be checked again.

Reviewer #3: Thank you for the opportunity to review this very important and interesting manuscript on the persistent schistosomiasis hotspots in Zimbabwe. This data is very useful in for better understanding hotspots dynamics over time.

In general:

- The manuscript needs an English review as some areas are quite unclear

o The use of many uncommon abbreviations creates confusion for the reader

- Continuous line numbers needs to be inserted to the manuscript

- It maybe useful to have more subheadings that follow the objectives in each section: e.g. Methods: Sample and setting, Sample collection and parasitology, Data collection, Prevalence and infection intensity, treatment efficacy, predictors of hotspots; Results: prevalence and infection intensity, treatment efficacy, predicotirs of hotspots etc. For more information the authors may want to use the following checklist https://www.equator-network.org/

- The manuscript should be reviewed for clarity and conciseness

o There is a quite a bit of repeating text and methods in the results and discussion (e.g. inclusion criteria in the methods and then again in the figure caption)

o The manuscript is quite long

o It is difficult to follow the objectives, through to the methods, results and discussion

**********

PLOS authors have the option to publish the peer review history of their article (what does this mean? ). If published, this will include your full peer review and any attached files.

If you choose “no”, your identity will remain anonymous but your review may still be made public.

**Do you want your identity to be public for this peer review?** For information about this choice, including consent withdrawal, please see our Privacy Policy .

Reviewer #1: No

Reviewer #2: No

Reviewer #3: No

[NOTE: If reviewer comments were submitted as an attachment file, they will be attached to this email and accessible via the submission site. Please log into your account, locate the manuscript record, and check for the action link "View Attachments". If this link does not appear, there are no attachment files.]

**Figure resubmission:**

While revising your submission, please upload your figure files to the Preflight Analysis and Conversion Engine (PACE) digital diagnostic tool, https://pacev2.apexcovantage.com/. PACE helps ensure that figures meet PLOS requirements. To use PACE, you must first register as a user. Registration is free. Then, login and navigate to the UPLOAD tab, where you will find detailed instructions on how to use the tool. If you encounter any issues or have any questions when using PACE, please email PLOS at figures@plos.org. Please note that Supporting Information files do not need this step. If there are other versions of figure files still present in your submission file inventory at resubmission, please replace them with the PACE-processed versions.

**Reproducibility:**

To enhance the reproducibility of your results, we recommend that authors of applicable studies deposit laboratory protocols in protocols.io, where a protocol can be assigned its own identifier (DOI) such that it can be cited independently in the future. Additionally, PLOS ONE offers an option to publish peer-reviewed clinical study protocols. Read more information on sharing protocols at https://plos.org/protocols?utm_medium=editorial-email&utm_source=authorletters&utm_campaign=protocols

Attachment Submitted filename: PLOS_review.docx

# Author comment: Author response to Decision Letter 1

**DOI:** 10.1371/journal.pntd.0013546.r002

10 Jun 2025

Attachment Submitted filename: revREBUTTAL Hotspots paper 2025 PLOS_reviewer comments.docx

# Peer review: Decision Letter 1

**DOI:** 10.1371/journal.pntd.0013546.r003

**Authors:** Francesca Tamarozzi

15 Jul 2025

PNTD-D-25-00047R1Identifying Hotspots of S. haematobium Infection Following Praziquantel Treatment During Multiple Annual Mass Drug Administration Campaigns in ZimbabwePLOS Neglected Tropical Diseases Dear Dr. Mduluza, Thank you for submitting your manuscript to PLOS Neglected Tropical Diseases. After careful consideration, we feel that it has merit but does not fully meet PLOS Neglected Tropical Diseases's publication criteria as it currently stands. Therefore, we invite you to submit a revised version of the manuscript that addresses the points raised during the review process.In particular, plase address concerns regarding the MDA samples size, which in some instance appear to be included with only 1 or 5 sample size. One revewer noted that many methods are still in the results section, which makes it difficult to discern what was exactly done. Please do divide clarly the methods (in the methods section) from the results (in the Results section). Please also revise figure 2 to make the dots bigger and clearer, revise Engligh (a revision by a native Enalish speaker is required to make the text clear), and try shorther overall the text, since this is very long. Despite PLoS NTD does not provide a maximum number of words, having a concide and clear text dhould be the aim.Please submit your revised manuscript within 30 days Aug 14 2025 11:59PM. If you will need more time than this to complete your revisions, please reply to this message or contact the journal office at plosntds@plos.org. When you're ready to submit your revision, log on to https://www.editorialmanager.com/pntd/ and select the 'Submissions Needing Revision' folder to locate your manuscript file. Please include the following items when submitting your revised manuscript:* A rebuttal letter that responds to each point raised by the editor and reviewer(s). You should upload this letter as a separate file labeled 'Response to Reviewers '. This file does not need to include responses to any formatting updates and technical items listed in the 'Journal Requirements' section below.* A marked-up copy of your manuscript that highlights changes made to the original version. You should upload this as a separate file labeled 'Revised Manuscript with Track Changes '.* An unmarked version of your revised paper without tracked changes. You should upload this as a separate file labeled 'Manuscript '. If you would like to make changes to your financial disclosure, competing interests statement, or data availability statement, please make these updates within the submission form at the time of resubmission. Guidelines for resubmitting your figure files are available below the reviewer comments at the end of this letter. We look forward to receiving your revised manuscript. Kind regards, Francesca TamarozziSection EditorPLOS Neglected Tropical Diseases Francesca TamarozziSection EditorPLOS Neglected Tropical Diseases

Shaden Kamhawi

co-Editor-in-Chief

PLOS Neglected Tropical Diseases

orcid.org/0000-0003-4304-636XX

Paul Brindley

co-Editor-in-Chief

PLOS Neglected Tropical Diseases

orcid.org/0000-0003-1765-0002

 **Journal Requirements:** Please ensure that the funders and grant numbers match between the Financial Disclosure field and the Funding Information tab in your submission form. Note that the funders must be provided in the same order in both places as well.

- State the initials, alongside each funding source, of each author to receive each grant. For example: "This work was supported by the National Institutes of Health (####### to AM; ###### to CJ) and the National Science Foundation (###### to AM)."

- State what role the funders took in the study. If the funders had no role in your study, please state: "The funders had no role in study design, data collection and analysis, decision to publish, or preparation of the manuscript.".

If you did not receive any funding for this study, please simply state: u201cThe authors received no specific funding for this work.u201d

  **Reviewers' comments:** Reviewer's Responses to Questions

**Key Review Criteria Required for Acceptance?**

As you describe the new analyses required for acceptance, please consider the following:

**Methods:**

-Are the objectives of the study clearly articulated with a clear testable hypothesis stated?

-Is the study design appropriate to address the stated objectives?

-Is the population clearly described and appropriate for the hypothesis being tested?

-Is the sample size sufficient to ensure adequate power to address the hypothesis being tested?

-Were correct statistical analysis used to support conclusions?

-Are there concerns about ethical or regulatory requirements being met?

Reviewer #2: The authors have adequately worked on my previous comments and hence I approve the manuscript for publication.

Reviewer #3: - the study design and exact objectives are still a bit confusing in the introduction and methods, but are very clear in the results and conclusion

**********

**Results**

-Does the analysis presented match the analysis plan?

-Are the results clearly and completely presented?

-Are the figures (Tables, Images) of sufficient quality for clarity?

Reviewer #2: Results are now well presented.

Reviewer #3: Not exactly, could still be improved i.e. move all methods to the methods section and clearly state what was done

**********

**Conclusions**

-Are the conclusions supported by the data presented?

-Are the limitations of analysis clearly described?

-Do the authors discuss how these data can be helpful to advance our understanding of the topic under study?

-Is public health relevance addressed?

Reviewer #2: The conclusion is sound and well informed by the findings presented.

Reviewer #3: - limitations should be improved - The short section on limitations doesn’t include how the limitations affect the results in terms of bias.

**********

**Editorial and Data Presentation Modifications?**

Use this section for editorial suggestions as well as relatively minor modifications of existing data that would enhance clarity. If the only modifications needed are minor and/or editorial, you may wish to recommend “Minor Revision” or “Accept”.

Reviewer #2: None

Reviewer #3: Abstract

-the abstract is quite long

-decreasing efficacy – the further they are from water the more likely they are to have decreasing efficacy? This seems odd...

Intro

Line 91-107 – I would recommend removing this paragraph. More or less, it states what is said in the previous paragraph, I think it is enough to just say that there are documented cases of persistent hotspots is Zanzibar and Kenya and that some studies have linked this issue to PZQ effectiveness (Senegal).

-Line 112-114 – what are the definitions of PPHS and EPHS exactly? Persistent hotspot of schistosomiasis prevalence simply means a hotspot, or?

Methods

Iine 135-136 – inclusion criteria for assessments? Or inclusion criteria for participants to participate were A) to have provided 3 urine samples both pre and post MDA? And B, I’m not sure what this means. Only treated children were included in the study? Or is it inclusion criteria for each arm of the study?

Line 168-171- this is unclear, how are persistent hotspots with prevalences of schistosomiasis determined by change in WHO prevalence risk category as meaningful decline in prevalence risk category? I would strongly suggest rephrasing.

Line 176 – best to say that this is a second approach for PPHS

Line 191- this aspect is still not reflected in the objectives in the introduction (ID transmission sites)

Results

Section 1 prevalence and egg count

- There is so much information and no visual representation, as a reader it is very difficult to follow all of this information and it is a lot of text (this section is almost 4 pages). Two graphs would be very helpful for the reader and could eliminate a lot of the text as the results are 9.5 pages. One graph with prevalence by district on y axis and time on x, plotting pre and post each MDA, the other graph the same but with Egg count. Also, the y axis could indicate the WHO categories of prevalence.

- There isn’t a description of what was compared, in the first section, the results go through preMDA1 to post MDA1. Then in the MDA2 section, there is comparison of both pre and post MDA1 and Pre MDA 2 , but this isn’t mentioned in the methods.

-please revise the sub-headings to something like Change in prevalence and egg count pre and post each MDA, just MDA1 is not very helpful

- Units should be present right after the number, not the CI e.g. 1.14 eggs/10ml (CI…)

- Timelines are often missing in the first paragraph and in the methods- changing the sub-headings could help

Line 257-263 - this should be in the methods

Line 270-271 –Where are the numbers, in a table perhaps? If so should refer to table here.

Line 273-280 – this isn’t clear, the paper discusses significant decrease in prevalence and then speaks of infection intensity in this and another district as an exception to having a statistically significant reduction in prevalence? Also, Line 273-274 says a change of prevalence from 66% to 24% wasn’t significant? Why is that, was the sample size small? Seems like a large difference. Lastly, decrease infection from pre-MDA1 until when?

Lines 351-354 – the reader needs both numbers here, both the pre-MDA1 and preMDA6 prevalences and egg counts

Efficacy

Lines 363-366 - this is methods should be in the methods, also the second sentence isn’t clear, inclusion criteria of the calculations?

Line367-368 – not sure what this means, can probably delete this text altogether, also seems to be methods, does it mean that the same children were not followed across all MDA?

Lines 367-369 - can be deleted

Lines 413-427 are methods, please move there, minus the reference to the tables

Line 380 – Mean egg counts expressed 95% CI – this text is out of place, please delete

Line 381-382 – this sentence needs to be rephrased, paired t test don’t change, it would be no significant difference between ….

Line 386-397 – should be in the methods

Line 386-387 – suggest changing to “post-MDA infection status for every village…"

Line 390 – not clear, should be rephrased

Line 404-405 – have the authors considered removing some districts from the analysis for some time points? For example, I wonder if MDAs with a sample size of 1 or 5 individuals should be included in analysis?

Line 413-427 and lines 454-463 and line 486 and lines 512-519 and lines 527-537 and 541-545– this is all methods and should be moved from the results to the methods

discussion

Lines 618-621 – this is unclear, I suggest rephrasing

Lines 640-642 – this is a very bold statement, I don’t think the results support this claim

- In regards to the 6 weeks and the limitations, PZQ only kills adults worms, so juveniles in the host during treatment would not be affected and could be releasing eggs at 6 weeks, when the post-treatment sampling was done.

Figures

Figure 1 is not mentioned in the text, also the caption has a description of methods (inclusion criteria) , which shouldn’t be there.

Fig 1 should be revised and redone, it is confusing and doesn’t present the data clearly

-doesn’t have appropriate column headings (MDA isn’t a study design, also n= is not prevalence, how many children is n, all together or in each region?)

-what are the brackets from pre and post for? What do they mean?

Figure 2

- The dots on the map, have not been addressed. It is better to indicate the site with a symbol, for example a circle and then next to that add another circle with the infection intensities that are bigger so that the reader can see them

Tables

Tables should be ordered in the order they appear in the text. The first table mentioned is Table 1 followed by Supptable 4. Therefore Supp table 4 should be supp table 1.

Table S4 – please show the unit of prevalence in table i.e. Prevalence in % (n)

-again, as before significant digits, all data has to be presented to the same decimal place (i.e. if you report one decimal point, 45.6, all numbers for this measure in the table should be to one decimal place)

**********

**Summary and General Comments**

Use this section to provide overall comments, discuss strengths/weaknesses of the study, novelty, significance, general execution and scholarship. You may also include additional comments for the author, including concerns about dual publication, research ethics, or publication ethics. If requesting major revision, please articulate the new experiments that are needed.

Reviewer #2: The current study examines in great depth and details the impact of repeated rounds of mass drug administration using praziquantel in school children in Zimbabwe and describes the development of Schistosoma haematobium transmission hotspots as it has been observed in many other African countries. The study has a sound design and methodology and the findings are credible. There is no doubt about the scientific merit and the public health relevance of the findings presented and hence I strongly recommend the article for publication and wider dissemination.

Reviewer #3: The authors have greatly improved the manuscript, but more work is needed. There remains issues around clarity and conciseness as well as methods in the results.

**********

PLOS authors have the option to publish the peer review history of their article (what does this mean? ). If published, this will include your full peer review and any attached files.

If you choose “no”, your identity will remain anonymous but your review may still be made public.

**Do you want your identity to be public for this peer review?** For information about this choice, including consent withdrawal, please see our Privacy Policy .

Reviewer #2: No

Reviewer #3: No

 [NOTE: If reviewer comments were submitted as an attachment file, they will be attached to this email and accessible via the submission site. Please log into your account, locate the manuscript record, and check for the action link "View Attachments". If this link does not appear, there are no attachment files.] **Figure resubmission:** While revising your submission, please upload your figure files to the Preflight Analysis and Conversion Engine (PACE) digital diagnostic tool, https://pacev2.apexcovantage.com/. PACE helps ensure that figures meet PLOS requirements. To use PACE, you must first register as a user. Registration is free. Then, login and navigate to the UPLOAD tab, where you will find detailed instructions on how to use the tool. If you encounter any issues or have any questions when using PACE, please email PLOS at figures@plos.org. Please note that Supporting Information files do not need this step. If there are other versions of figure files still present in your submission file inventory at resubmission, please replace them with the PACE-processed versions. **Reproducibility:** To enhance the reproducibility of your results, we recommend that authors of applicable studies deposit laboratory protocols in protocols.io, where a protocol can be assigned its own identifier (DOI) such that it can be cited independently in the future. Additionally, PLOS ONE offers an option to publish peer-reviewed clinical study protocols. Read more information on sharing protocols at https://plos.org/protocols?utm_medium=editorial-email&utm_source=authorletters&utm_campaign=protocols

# Author comment: Author response to Decision Letter 2

**DOI:** 10.1371/journal.pntd.0013546.r004

17 Aug 2025

Attachment Submitted filename: REBUTTAL Hotspots paper Aug2025 .docx

# Peer review: Decision Letter 2

**DOI:** 10.1371/journal.pntd.0013546.r005

**Authors:** Francesca Tamarozzi

9 Sep 2025

Dear Professor Mduluza,

We are pleased to inform you that your manuscript 'Identifying Hotspots of S. haematobium Infection Following Praziquantel Treatment During Multiple Annual Mass Drug Administration Campaigns in Zimbabwe' has been provisionally accepted for publication in PLOS Neglected Tropical Diseases.

Before your manuscript can be formally accepted you will need to complete some formatting changes, which you will receive in a follow up email. A member of our team will be in touch with a set of requests.

Please note that your manuscript will not be scheduled for publication until you have made the required changes, so a swift response is appreciated.

IMPORTANT: The editorial review process is now complete. PLOS will only permit corrections to spelling, formatting or significant scientific errors from this point onwards. Requests for major changes, or any which affect the scientific understanding of your work, will cause delays to the publication date of your manuscript.

Should you, your institution's press office or the journal office choose to press release your paper, you will automatically be opted out of early publication. We ask that you notify us now if you or your institution is planning to press release the article. All press must be co-ordinated with PLOS.

Thank you again for supporting Open Access publishing; we are looking forward to publishing your work in PLOS Neglected Tropical Diseases.

Best regards,

Francesca Tamarozzi

Section Editor

PLOS Neglected Tropical Diseases

Francesca Tamarozzi

Section Editor

PLOS Neglected Tropical Diseases

Shaden Kamhawi

co-Editor-in-Chief

PLOS Neglected Tropical Diseases

orcid.org/0000-0003-4304-636XX

Paul Brindley

co-Editor-in-Chief

PLOS Neglected Tropical Diseases

orcid.org/0000-0003-1765-0002

***********************************************************

Reviewer #3:

Reviewer's Responses to Questions

**Key Review Criteria Required for Acceptance?**

As you describe the new analyses required for acceptance, please consider the following:

**Methods**

-Are the objectives of the study clearly articulated with a clear testable hypothesis stated?

-Is the study design appropriate to address the stated objectives?

-Is the population clearly described and appropriate for the hypothesis being tested?

-Is the sample size sufficient to ensure adequate power to address the hypothesis being tested?

-Were correct statistical analysis used to support conclusions?

-Are there concerns about ethical or regulatory requirements being met?

Reviewer #3: (No Response)

**********

**Results**

-Does the analysis presented match the analysis plan?

-Are the results clearly and completely presented?

-Are the figures (Tables, Images) of sufficient quality for clarity?

Reviewer #3: (No Response)

**********

**Conclusions**

-Are the conclusions supported by the data presented?

-Are the limitations of analysis clearly described?

-Do the authors discuss how these data can be helpful to advance our understanding of the topic under study?

-Is public health relevance addressed?

Reviewer #3: (No Response)

**********

**Editorial and Data Presentation Modifications?**

<br/>

Use this section for editorial suggestions as well as relatively minor modifications of existing data that would enhance clarity. If the only modifications needed are minor and/or editorial, you may wish to recommend “Minor Revision” or “Accept”.

Reviewer #3: - Tables 1 and 2 don't have table captions, while table 1 is mostly intuitive, table 2 should be explained in a table caption

- figure 1 still has the top row, which is confusing as it looks like column headings. It isn't clear to me what that top row means

-figure 2 site markers which also show infection intensity remain small and difficult to see

**********

**Summary and General Comments**

Use this section to provide overall comments, discuss strengths/weaknesses of the study, novelty, significance, general execution and scholarship. You may also include additional comments for the author, including concerns about dual publication, research ethics, or publication ethics. If requesting major revision, please articulate the new experiments that are needed.

Reviewer #3: The manuscript has improved considerably in terms of clarity.

**********

PLOS authors have the option to publish the peer review history of their article (what does this mean? ). If published, this will include your full peer review and any attached files.

If you choose “no”, your identity will remain anonymous but your review may still be made public.

**Do you want your identity to be public for this peer review?** For information about this choice, including consent withdrawal, please see our Privacy Policy .

Reviewer #3: No

# Editor report: Acceptance letter

**DOI:** 10.1371/journal.pntd.0013546.r006

**Authors:** Francesca Tamarozzi

Dear Professor Mduluza,

We are delighted to inform you that your manuscript, " 

Identifying Hotspots of S. haematobium Infection Following Praziquantel Treatment During Multiple Annual Mass Drug Administration Campaigns in Zimbabwe

" has been formally accepted for publication in PLOS Neglected Tropical Diseases.

We have now passed your article onto the PLOS Production Department who will complete the rest of the publication process. All authors will receive a confirmation email upon publication.

The corresponding author will soon be receiving a typeset proof for review, to ensure errors have not been introduced during production. Please review the PDF proof of your manuscript carefully, as this is the last chance to correct any scientific or type-setting errors. Please note that major changes, or those which affect the scientific understanding of the work, will likely cause delays to the publication date of your manuscript. Note: Proofs for Front Matter articles (Editorial, Viewpoint, Symposium, Review, etc...) are generated on a different schedule and may not be made available as quickly.

Soon after your final files are uploaded, the early version of your manuscript will be published online unless you opted out of this process. The date of the early version will be your article's publication date. The final article will be published to the same URL, and all versions of the paper will be accessible to readers.

You will receive an invoice from PLOS for your publication fee after your manuscript has reached the completed accept phase. If you receive an email requesting payment before acceptance or for any other service, this may be a phishing scheme. Learn how to identify phishing emails and protect your accounts at https://explore.plos.org/phishing.

Thank you again for supporting open-access publishing; we are looking forward to publishing your work in PLOS Neglected Tropical Diseases.

Best regards,

Shaden Kamhawi

co-Editor-in-Chief

PLOS Neglected Tropical Diseases

Paul Brindley

co-Editor-in-Chief

PLOS Neglected Tropical Diseases
